# Direct additive-free *N*-formylation and *N*-acylation of anilines and synthesis of urea derivatives using green, efficient, and reusable deep eutectic solvent ([ChCl][ZnCl_2_]_2_)

**DOI:** 10.1038/s41598-024-57608-8

**Published:** 2024-03-26

**Authors:** Fatemeh Abbasi, Ali Reza Sardarian

**Affiliations:** Chemistry Department, College of Sciences, Shiraz University, Shiraz, 71946-84795 Iran

**Keywords:** *N*-Formylation, *N*-Acylation, Deep eutectic solvent, Unsymmetrical ureas, Acetic acid, Formamide, Phenyl isocyanate, Catalysis, Green chemistry, Organic chemistry, Chemical synthesis

## Abstract

In the current report, we introduce a simple, mild efficient and green protocol for *N*-formylation and *N*-acetylation of anilines using formamide, formic acid, and acetic acid as inexpensive, nontoxic, and easily available starting materials just with heating along stirring in [ChCl][ZnCl_2_]_2_ as a durable, reusable deep eutectic solvent (DES), which acts as a dual catalyst and solvent system to produce a wide range of formanilides and acetanilides. Also, a variety of unsymmetrical urea derivatives were synthesized by the reaction of phenyl isocyanate with a range of amine compounds using this benign DES in high to excellent yields. [ChCl][ZnCl_2_]_2_ showed good recycling and reusability up to four runs without considerable loss of its catalytic activity.

## Introduction

**Figure 1:**
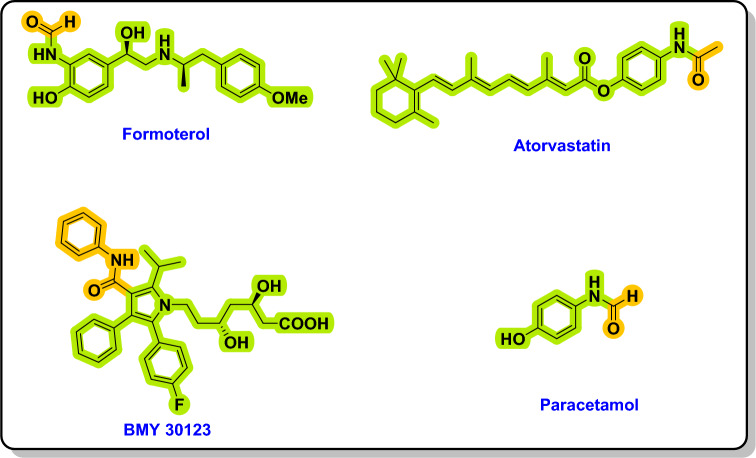
Amide frameworks in bioactive substances.

Amides are an extremely significant class of compounds with multiple uses in both the commercial and academic communities^1^, which are used in drugs (Fig. 1)^2,3^, dyes, natural products, and a wide range of polymers^4–6^. *N*-aryl carboxamides are an important amide class that is found in numerous medications^7^. For example, a long-acting β_2_-agonist used in the treatment of asthma and chronic obstructive pulmonary disease is formoterol, atorvastatin is used as a medicine to treat dyslipidemia and prevent cardiovascular diseases^8,9^ and paracetamol is widely used as a non-narcotic pain reliever and fever medicine^10,11^. A significant problem in organic chemistry is developing a practical and effective synthetic method for amide bond formation given the considerable importance of amide in biological systems and medicinal chemistry. As a result, numerous synthetic methods to generate amide bonds have been suggested.

There are a variety of synthetic processes that can be used to obtain amides, including the reaction of carboxylic acid derivatives (other than amides) with amines or ammonia^12–14^, the reaction of amines with aldehydes or alcohols^15–17^, or formamide^18,19^ and the hydration of nitriles^20^. Direct amidation, which involves the use of aromatic or aliphatic amines with carboxylic acid in the presence of stoichiometric amounts of activating reagents, is the common method and most used technique for forming amide bonds^21,22^. Huge costs, high temperature, considerable amount of chemical waste generation, and potential environmental issues have led to the development of several alternative synthetic methods^23^, among which transamidation reaction is a common nucleophilic acyl substitution method in synthetic organic chemistry between a carboxamide and an amine^24–26^. Since uncatalyzed transamidation requires extremely high temperatures, numerous techniques have been developed to resolve this matter by utilizing catalysts or activating reagents^13,27^. Although the direct transamidation method appears to be relatively uncommon because of the low electrophilic character of the carbonyl amide group, it is possible to activate this functional group with suitable catalytic systems and promote the generation of new carboxamide derivatives^28^. There have been some innovative transamidation examples that utilize both homogeneous and heterogeneous catalysts like CeO_2_^29^, Ni(quin)_2_^30^, Pd(OAc)_2_^11^, Fe(NO_3_)_3_·9H_2_O^13^, Pd/NHC(*N*-Heterocyclic Carbene) complexes^31^, AlCl_3_^32^, Sc(OTf)_3_^33^, Cu(OAc)_2_^34^, Zirconocene dichloride (Cp_2_ZrCl_2_)^35^, hypervalent iodobenzene diacetate^36^. In addition, Et_3_N^37,38^, L-proline^39^, chitosan^40^, ionic liquid^41^, and boric acid^42^. Although the existing methodologies have their advantages, all of the reported protocols suffer from some disadvantages including costly and specialized transition metal catalysts, consuming stoichiometric amounts of the catalysts, prolonged reaction times, harsh reaction conditions, and use of dangerous organic solvents. Therefore, access to more efficient and environmentally friendly transamidation technologies is needed to solve the above-mentioned problems.

**Figure 2:**
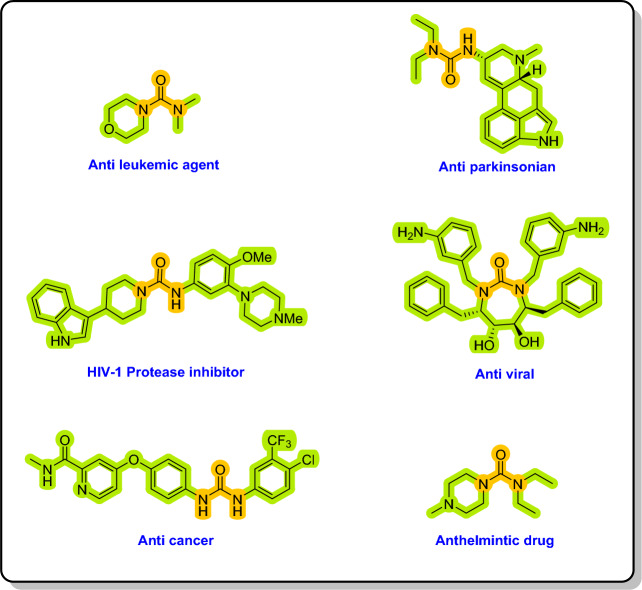
Biologically active unsymmetrical urea derivatives.

Ureas and their derivatives are extremely significant nitrogen-containing carbonyl compounds that are abundantly present in both natural and manufactured substances^43^. In addition, many biologically active substances bear urea units in their structures such as anti-mycobacterial^44,45^, anti-fungal^46^, anti-tumor^47,48^, antagonists of natural receptors^49^, and enzyme inhibitors^50^. They have been deeply studied in many fields including infectious diseases such as malaria^51^ and tuberculosis^52,53^, immunology^54^, oncology^55^. Urea and its derivatives belonging to a recognized class of molecules are widely used in agrochemistry^56^, material science^57^, organic syntheses^58,59^ and medicinal chemistry^60,61^. Many urea derivatives have biological properties, some of which are shown in Fig. 2^62,63^.

Due to their significance in numerous sectors, the synthesis of symmetrical and asymmetrical urea derivatives has drawn a lot of attention. Several synthetic processes have been reported for the creation of urea structures^64,65^. Many transition metal-catalyzed processes including metals like Pd^66,67^, Mn^68^, W^69^, Au^70^, Ni^71^, and Ru^72^ have been used to synthesize urea. Conventional approaches, such as the interaction of amines with commercially or in situ-produced isocyanates, continue to be one of the most straightforward and accessible ways to obtain these molecules^73,74^. Although different methods have been used for the synthesis of urea structures, they are not without flaws. Low yields of the products, using expensive and complicated catalysts or reagents, long reaction periods, and several-step isolation procedures limit their use in practical applications. In light of their numerous crucial applications, they have gained a lot of attention in the development of innovative, effective, selective, and environmentally friendly protocols for the synthesis of ureas^75^. Therefore, it would be advantageous to develop an effective process for the synthesis of ureas without the use of hazardous organic solvents, hazardous or corrosive reagents, or expensive catalysts.

**Figure 3:**
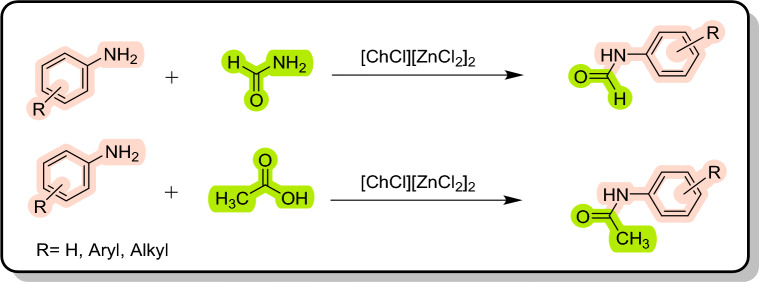
Formylation and acylation of anilines in [ChCl][ZnCl_2_]_2_ as a dual solvent/catalyst.

**Figure 4:**
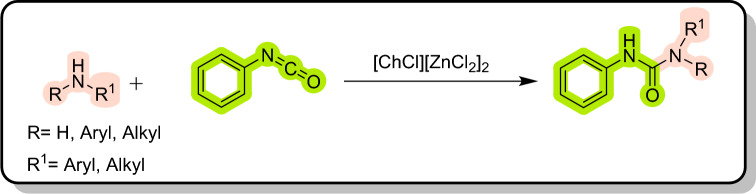
Synthesis of urea derivatives in [ChCl][ZnCl_2_]_2_ as a dual solvent/catalyst.

Regarding to special features of deep eutectic solvents (DESs) as a class of ionic liquids^76^ such as low vapor pressure, biodegradable and environmentally friendly systems, easy atom economic preparation, and excellent chemical and thermal stability^77^, they have received a lot of attention over the last decade. Thus, within this context and our ongoing research in the application of DESs in organic chemistry^78–80^, we plan to employ [ChCl][ZnCl_2_]_2_ DES as a green solvent/catalyst system for the first time in the synthesis of carboxamides, such as formanilides, acetanilides (Fig. 3), and also urea derivatives (Fig. 4).

## Experimental section

### General

All of the chemicals were purchased from chemical companies and used with no more purification. The thin layer chromatography (TLC) plates were made by Merck Silica gel 60 F254. The ^1^H and ^13^C NMR spectra were recorded using a Bruker-400 MHz NMR spectrometer apparatus at ambient temperature (ppm), and the signals are expressed at a downfield of TMS (δ 0.00) as an internal standard in parts per million for each spectrum. For ^1^H NMR, the following parameters are displayed: chemical shift (ppm, scale), multiplicity (s = singlet, d = doublet, t = triplet, q = quartet, and m = multiplet resonances), coupling constant (Hz), and integration data. Data for ^13^C NMR’s chemical shift (ppm, scale), multiplicity, and coupling constant are presented (Hz). Fourier transform infrared (FT-IR) spectra of the compounds were recorded at room temperature using a Shimadzu FT-IR 8300 spectrophotometer between 400 and 4000 cm^−1^. Thermofinnigan's Flash EA-1112 CHNS rapid elemental analyzer was employed to determine the elemental composition of products. Melting points were measured using an Electrothermal 9100 instrument.

### Preparation of [ChCl][ZnCl_2_]_2_ as deep eutectic solvent^81–83^

Choline chloride (ChCl) and zinc chloride (ZnCl_2_) were used as the hydrogen bond donors and acceptors respectively in the production of this DES. Zinc(II) chloride (2 mmol, 0.272 g) and choline chloride (1 mmol, 0.139 g) were mixed, and the resulting combination was heated in an oil bath at 100 °C until it became a transparent, homogenous, and colourless liquid. It was then allowed to cool at room temperature and used without further purification.

### General procedure for the preparation of formanilides from formamide (C1-13 in Table 2)

Into a 5 mL round bottom flask containing 6 mmol (3 mL) of DES ([ChCl][ZnCl_2_]_2_), amine derivatives (1 mmol) and formamide (1 mmol) were added and the mixture was vigorously stirred for 3.5 h at 80 °C. TLC was used to track the reaction's progress (ethyl acetate/*n*-hexane, 1:5). Upon completion of the reaction, the reaction mixture was cooled down to the ambient temperature. The reaction mixture was then diluted with water (10 mL) and extracted using ethyl acetate (2 × 5 mL). The organic layer was dried over anhydrous MgSO_4_, filtered, and concentrated with a rotary evaporator to generate the corresponding crude product. The pure product was provided by recrystallizing the crude product in ethanol/*n*-hexane. The product's identity and purity were all validated by FT-IR, ^1^H NMR, ^13^C NMR, and CHNS analyses.

The remaining aqueous solution was then heated at 70 °C for 30 min under a reduced vacuum to provide the dry DES, which can be utilized in the next run without further activation.

### General procedure for the preparation of acetanilide and formanilide from formic and acetic acid (C17-26 in Table 4)

Amine derivatives (1 mmol), acetic acid (1 mmol, 0.06 mL), or formic acid (1 mmol, 0.04 mL) were placed into a 5 mL round bottom flask containing 6 mmol (3 mL) of the DES ([ChCl][ZnCl_2_]_2_) and heated at 70 °C under solvent-free magnetic stirring for 3 h. The progress of the reaction was monitored by TLC (ethyl acetate/*n*-hexane (1:5)). The reaction mixture was cooled down to room temperature after the reaction's completion. Next, the reaction mixture was diluted with 10 mL water and extracted with ethyl acetate (2 × 5 mL). To produce the corresponding crude product, the ethyl acetate layer was dried over anhydrous MgSO_4_, filtered, and concentrated using a rotary evaporator. By recrystallizing in ethanol and *n*-hexane, the pure product was obtained. FT-IR, ^1^H NMR, ^13^C NMR, and CHNS analysis techniques were used to determine the product's purity and identification. The dry DES was obtained after heating the extract aqueous layer at 70 °C for 30 min under a reduced vacuum.

### General procedure for the preparation of urea derivatives (C28–38 in Table 7)

Amine derivatives (1 mmol) and phenyl isocyanate (1 mmol) were mixed and stirred vigorously for a specified time at 50 °C in a 5 mL round bottom flask containing 6 mmol (3 mL) of the DES ([ChCl][ZnCl_2_]_2_). The reaction's progress was monitored using TLC (ethyl acetate/*n*-hexane (1:5)). Then, the reaction mixture was cooled to reach room temperature. Afterward, the reaction mixture was diluted with 10 mL water and extracted with ethyl acetate (2 × 5 mL). The merged organic layer was dried over anhydrous MgSO_4_, filtered, and evaporated using a rotary evaporator. Finally, the acquired crude was refined by recrystallization with ethanol/*n*-hexane. The identification and purity of the product have been verified by FT-IR, ^1^H, ^13^C NMR, and CHNS analyses. The extracted aqueous layer was heated for 30 min at 70 °C under reduced pressure to afford the dried DES, which can be directly employed in the next reaction.

## Results and discussion

### Optimization of the reaction factors for the synthesis of formanilides

To optimize the reaction conditions, formamide (**A**) and aniline (**B**) were chosen as model substrates and the effect of the molar ratio of reagents, various amounts of DES, reaction time, and temperature were studied. The findings are reported in Table 1. First, the catalytic activity of a wide range of readily accessible choline chloride-based DESs was investigated in the model reaction (Table 1, entries 1–10). According to the findings in Table 1 (entries 6–10), DESs prepared from choline chloride and metal chlorides exhibited better efficiency as solvent/catalyst systems and the best result was produced when the model reaction was performed in [ChCl][ZnCl_2_]_2_ [ChCl][ZnCl_2_]_2_ (Table 1, entry 7). The reaction was done in the absence of to demonstrate the key role of DES. After 24 h, the desired product was found in small amounts (Table 1, entry 11), These results show that [ChCl][ZnCl_2_]_2_ is essential for the reaction to proceed and the best results were obtained when the molar ratio of ChCl:ZnCl_2_ was 1:2 (Table 1, entries 7, 15 and 16). The model reaction was then performed using ZnCl_2_, and a lower yield of the amide product was achieved (Table 1, entry 12). The effect of [ChCl][ZnCl_2_]_2_ as a solvent/catalyst system in various amounts was then studied (Table 1, entries 7,13 and 14) and using 6 mmol the DES produced the highest yield of the expected product. It should also be mentioned that the effects of different molar ratios of aniline and formamide in the model reaction were investigated, and it revealed that although all examined various molar ratios were effective but large amounts of raw materials remained intact when the molar ratio less or higher than 1:1 was applied and made no significant impact on the amount of desired product (Table 1, entries 7, 17 and 18). Additionally, it was observed that the transamidation reaction efficiency was sensitive to the reaction temperature and the product yield reduced noticeably by decreasing temperature and reached its highest yield at 80 °C (Table 1, entries 7, 19–21). The investigation of the reaction time was also considered next (Table 1, entries 7, and 22–24). Finally, it was found that the highest yield of the amide was produced at 80 °C in 3.5 h by the reaction of aniline (1 mmol) and formamide (1 mmol) in the presence of 3 mL (6 mmol) of [ChCl][ZnCl_2_]_2_ (Table 1, entry 7).

**Table 1:** Optimization of reaction parameters for one-pot synthesis of *N*-phenyl formamides.


Entry	Molar ratio A:B	DES	DES (mmol)	Molar mass of DES (g mol^−1^)^a^	Temperature (°C)	Time (h)	Yield (%)^b^
1	1:1	[ChCl][Urea]_2_	6	86.58	80	3.5	29
2	1:1	[ChCl][Thiourea]_2_	6	97.29	80	3.5	38
3	1:1	[ChCl][Glycerol]_2_	6	107.93	80	3.5	49
4	1:1	[ChCl][TEA]_2_	6	146	80	3.5	42
5	1:1	[ChCl][BA]_2_	6	127.95	80	3.5	31
6	1:1	[ChCl][FeCl_3_]_2_	6	132.94	80	3.5	72
**7**	**1:1**	**[ChCl][ZnCl**_**2**_**]**_**2**_	**6**	**137.41**	**80**	**3.5**	**91**
8	1:1	[ChCl][SnCl_2_]_2_	6	154.67	80	3.5	83
9	1:1	[ChCl][NiCl_2_]_2_	6	172.94	80	3.5	56
10	1:1	[ChCl][CuCl_2_]_2_	6	136.17	80	3.5	67
11	1:1	–	–	–	80	3.5	23
12	1:1	ZnCl_2_	–	–	80	3.5	51
13	1:1	[ChCl][ZnCl_2_]_2_	4	137.41	80	3.5	86
14	1:1	[ChCl][ZnCl_2_]_2_	8	137.41	80	3.5	91
15	1:1	[ChCl][ZnCl_2_]	6	137.62	80	3.5	80
16	1:1	[ChCl]_2_[ZnCl_2_]	6	137.12	80	3.5	71
17	1:2	[ChCl][ZnCl_2_]_2_	6	137.41	80	3.5	86
18	2:1	[ChCl][ZnCl_2_]_2_	6	137.41	80	3.5	79
19	1:1	[ChCl][ZnCl_2_]_2_	6	137.41	70	3.5	73
20	1:1	[ChCl][ZnCl_2_]_2_	6	137.41	90	3.5	91
21	1:1	[ChCl][ZnCl_2_]_2_	6	137.41	100	3.5	87
22	1:1	[ChCl][ZnCl_2_]_2_	6	137.41	80	2	79
23	1:1	[ChCl][ZnCl_2_]_2_	6	137.41	80	5	91
24	1:1	[ChCl][ZnCl_2_]_2_	6	137.41	80	7	91

^a^HBD, Hydrogen bond donor; HBA, Hydrogen bond acceptor. The molecular mass (*M*_DES_) of DES is measured from Eq: ${M}_{{\text{DES}}}=\frac{{x}_{{\text{HBA}}}*{M}_{{\text{HBA}}}+{x}_{{\text{HBD}}}*{M}_{{\text{HBD}}}}{{x}_{{\text{HBA}}}+{x}_{{\text{HBD}}}}$, where M_DES_ is the molecular mass of DES in g.mol^−1^, χ _HBA_ and x_HBD_ are the mole ratio of the HBA and HBD respectively; M_HBD_ and M_HBA_ are the molecular mass of the HBD and HBA in g mol^−1^.

^b^Isolated yield.

Under optimal conditions, aniline derivatives with both electron-donating and electron-withdrawing substituents underwent efficiently this conversion. However, anilines with electron-withdrawing groups had slightly lower yields than those of anilines with electron-donating groups (Table 2, entries C1–13).

**Table 2:** Synthesis of formanilides in [ChCl][ZnCl_2_]_2_ as a solvent/catalyst system.


Substrate	Substrate	Product	Time (h)	Yield^a^ (%)	M.p. °C (Lit)
			4	88	105 (103–105)^84^
			3.5	72	126 (121–122)^85^
			3.5	89	51 (50–52)^86^
			3.5	85	76 (73–77)^87^
			4	83	156–157 (155–157)^88^
			4	68	138 (135–137)^88^
			4.15	66	177 (175–177)^89^
			4.45	72	179
			3.5	91	53 (48–51)^90^
			4	74	79 (78.8–79.8)^91^
			3.5	81	55 (51–53)^92^
			4	68	135 (129–132)^92^
			4	87	186 (182–187)^93^
			8	< 5	–
			10	NR^b^	–
			10	NR^b^	–

Reaction conditions: Aniline derivatives (1.0 mmol), formamide (1.0 mmol) and 3 mL of [ChCl][ZnCl_2_]_2_.

^a^Isolated yield.

^b^No reaction.

It should be noted that instead of primary aromatic amines, secondary aromatic amines did not undergo *N*-formylation well and provided the related product in less than 5%. (Table 2, entry C14). Also, both types of aliphatic amines such as benzylamine and morpholine did not take part in the reaction with formamide (Table 2, entries C15–16).

For evaluating the efficiency of [ChCl][ZnCl_2_]_2_ in scaling up the present *N*-formylation reaction, an experiment was performed using 10 mmol of aniline and 10 mmol of formamide in 30 mL of the DES at 80 °C. After 3.5 h, the related formanilide was isolated from the reaction mixture in 90% yield.

**Figure 5:**
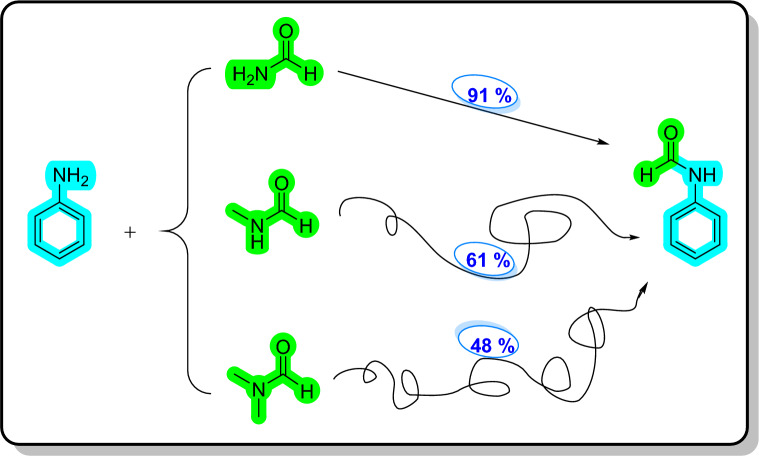
Comparison of the reactivity of formamide, *N*-methylformamide and *N,N*-dimethylformamide toward aniline as substrates.

We also applied *N*-methylformamide and *N*,*N*-dimethylformamide as substrates instead of formamide, which displayed low to moderate efficiency (Fig. 5).

In continuation, it was scheduled to provide formanilides and acetanilides from the reaction of anilines with formic and acetic acid in the presence of DES.

### Optimization of reaction parameters of carboxylic acids with anilines

At first, aniline was treated individually with benzoic acid, acetic acid and formic acid but the expected reaction with benzoic acid did not occur at all. The best reaction efficiencies were obtained when formic acid and acetic acid were used. Therefore, we selected the reaction of acetic acid with aniline as the model to optimize the reaction parameters. The desired reaction was carried out in different conditions, during which a series of parameters including the molar ratio of reagents, types of DES, reaction time, and ultimately temperature were investigated (Table 3). First, in the model reaction, the catalytic activity of a variety of choline chloride-based DESs was examined. The data collected in Table 3 (Entries 1–6) disclosed that the DESs synthesized from choline chloride and metal halides afforded higher yields of acetanilide (**C**) (Entries 4–6) and the highest efficiency was achieved when the reaction was carried out in 6mmol of ChCl/ZnCl_2_ at 80 °C after 3.5 h (Entry 4). The reaction was carried out without using any DES to assess its performance. A significant reduction in the efficiency of the reaction occurred, which demonstrates the requirement of the reaction to the DES for its completion (Table 3, entry 7).

**Table 3:** Optimization of reaction parameters for one-pot synthesis of *N*-acyl aniline.


Entry	Molar ratio A:B	DES	DES (mmol)	Molar mass of DES (g mol^−1^)^a^	Temperature (°C)	Time (h)	Yield (%)^b^
1	1:1	[ChCl][Urea]_2_	6	86.58	80	3.5	29
2	1:1	[ChCl][Glycerol]_2_	6	107.93	80	3.5	49
3	1:1	[ChCl][BA]_2_	6	127.95	80	3.5	31
4	1:1	[ChCl][ZnCl_2_]_2_	6	137.41	80	3.5	91
5	1:1	[ChCl][SnCl_2_]_2_	6	154.67	80	3.5	83
6	1:1	[ChCl][NiCl_2_]_2_	6	172.94	80	3.5	56
7	1:1	–	–	–	80	3.5	23
8	1:1	ZnCl_2_	–	–	80	3.5	51
9	1:1	[ChCl]	–	–	80	3.5	28
10	1:1	[ChCl][ZnCl_2_]_2_	4	137.41	80	3.5	86
11	1:1	[ChCl][ZnCl_2_]_2_	8	137.41	80	3.5	91
12	1:1	[ChCl][ZnCl_2_]	6	137.62	80	3.5	80
13	1:1	[ChCl]_2_[ZnCl_2_]	6	137.12	80	3.5	71
14	1:2	[ChCl][ZnCl_2_]_2_	6	137.41	80	3.5	86
15	2:1	[ChCl][ZnCl_2_]_2_	6	137.41	80	3.5	79
16	1:1	[ChCl][ZnCl_2_]_2_	6	137.41	rt	3.5	73
17	1:1	[ChCl][ZnCl_2_]_2_	6	137.41	60	3.5	73
18	1:1	[ChCl][ZnCl_2_]_2_	6	137.41	90	3.5	91
19	1:1	[ChCl][ZnCl_2_]_2_	6	137.41	80	2	79
20	1:1	[ChCl][ZnCl_2_]_2_	6	137.41	80	5	91
21	1:1	[ChCl][ZnCl_2_]_2_	6	137.41	80	7	91

^a^HBD, Hydrogen bond donor; HBA, Hydrogen bond acceptor. The molecular mass (*M*_DES_) of DES is measured from Eq: ${M}_{{\text{DES}}}=\frac{{x}_{{\text{HBA}}}*{M}_{{\text{HBA}}}+{x}_{{\text{HBD}}}*{M}_{{\text{HBD}}}}{{x}_{{\text{HBA}}}+{x}_{{\text{HBD}}}}$, where M_DES_ is the molecular mass of DES in g mol^−1^, χ_HBA_ and x_HBD_ are the mole ratio of the HBA and HBD respectively; M_HBD_ and M_HBA_ are the molecular mass of the HBD and HBA in g mol^−1^.

^b^Isolated yield.

The desired product was also formed when the model reaction was run in the presence of ZnCl_2_ or ChCl alone, however, the reaction efficiency was lower than when DES was applied (Table 3, entries 8 and 9). The model reaction was then examined, in addition to 6mmol, in 4 and 8 mmol of DES, in which the lower yield of the related product was produced (Table 3, entries 10 and 11). On the other hand, Table 3, entries 4, 12, and 13 showed that the greatest results were obtained when the molar ratio of zinc chloride to choline chloride was two to one. Additionally, several molar ratios of acetic acid to aniline were examined in the model reaction and it was found that the best outcome is obtained when a molar ratio is 1:1 (Table 3, entries 14 and 15). We also took notice that the model's reaction was effectively temperature-dependent and as temperature decreased, the production efficiency of the required amide dramatically decreased and the highest result was achieved at 80 °C (Table 3, entries 16–18). Finally, the reaction time was considered and clarified that the reaction afforded the largest amount of the related product after 3.5 h (Table 3, entries 19–21). Consequently, it was discovered that the reaction of aniline (1 mmol) and acetic acid (1 mmol) in the presence of 3 mL (6 mmol) [ChCl][ZnCl_2_]_2_ produced the maximum yield of amide at 80 °C in 3.5 h (Table 3, entry 4).

After getting the optimized reaction parameters, a variety of anilines were treated with formic acid and acetic acid to produce the title products in moderate to high yields, which the related data were registered in Table 4. In general, the reaction of primary anilines was more efficient with formic acid than acetic acid and primary anilines with electron-withdrawing substituent provided lower yield of the expected amides than aniline. *N*-methylaniline, as a secondary aniline, did not undergo well the transamidation and supplied **C27** in less than 5% yield even in a longer time, 50 min. Also *o*-nitroaniline, which bears an electron-withdrawing substituent with some extent *ortho* steric hindrance, carried out the reaction and furnished the corresponding acetanilide (**C24**) in moderate yield.

**Table 4:** The reaction of aniline derivatives with carboxylic acids in [ChCl][ZnCl_2_]_2_ as a solvent/catalyst system.


Amine	Carboxylic acid	Product	Time (min)	Yield^a^ (%)	M.p. °C (Lit)
			30	87	53 (48–51)^90^
			30	81	116 (114–116)^94^
			30	67	171 (169.2–170.8)^95^
			30	81	186 (182–187)^93^
			30	74	69 (65.2–67)^95^
			30	83	105 (103–105)^84^
			30	77	184–186 (178–179)^96^
			50	47	93 (91–93)^97^
			50	56	154 (154–155)^98^
			50	64	208 (206–208)^99^
			50	< 5	–

Reaction conditions: Aniline derivatives (1.0 mmol), carboxylic acid derivatives (1.0 mmol) and 3 mL of [ChCl][ZnCl_2_]_2_ as a solvent/catalyst.

^a^Isolated yield.

To investigate the value of this work, the results obtained from the application of DES in *N*-formylation and *N*-acylation of anilines were compared with the outcome of the catalyzed *N*-formylation and *N*-acylation of amines by some of the reported methods in the literature (Table 5). Although each of the methods mentioned has its advantages but usually suffers from some problems, such as long reaction time and utilization rate of catalyst, and also some of them require high temperatures to be done. As you can see, the DES catalyst is one of the most efficient (Table 5, entry 6). Moreover, the synthesis of this catalyst is easy and cost-effective using biocompatible, biodegradable, and inexpensive available raw materials and it is also used as a reaction solvent. As a result, the catalyst system works very well, which can achieve the desired product in almost a short time and high to good efficiency.

**Table 5:** Comparison of different conditions for *N*-formylation and *N*-acylation of amines reaction.

Entry	Catalyst (g)	Reaction conditions	Time (h)	Yield (%)	References
1	Fe(OH)_3_@Fe_3_O_4_ NPs (0.030)	*p*-xylene, 140 °C	10	92	^18^
2	Fe_3_O_4_-CA (0.025)	Neat, 120 °C	8	89	^100^
3	Graphene Oxide (0.050)	Neat, 150 °C	24	76	^19^
4	Fe_3_O_4_-OSO_3_H (0.025)	Neat, 120 °C	2	96	^101^
5	Free catalyst	Neat, 150 °C, Under argon	24	92	^102^
6	[ChCl][ZnCl_2_]_2_ (0.82)	DES, 80 °C	3.5	91	This work

We also tested butyric acid, caproic acid, acetic anhydride, acetonitrile, and ethyl acetate as raw materials instead of acetic acid. Except for butyric acid and caproic acid, which did not produce the expected products (Table 6, entries 2 and 3), the others gave the desired product in low to good yields (Table 6, entries 1 and 4–6).

**Table 6:** The reaction of aniline with acylating agents in [ChCl][ZnCl_2_]_2_ as a solvent/catalyst system.

Entry	Amine	Acylation reagent	Product	Yield^a^ (%)
1				81
2				NR^b^
3				NR^b^
4				79
5				19
6				28

Reaction conditions: Aniline (1.0 mmol), acylating agents (1.0 mmol) and 3 mL of [ChCl][ZnCl_2_]_2_ as a solvent/catalyst.

^a^Isolated yield.

^b^No reaction.

In continuing the present research and finding the potential of [ChCl][ZnCl]_2_ as a dual solvent/catalyst system, the treatment of phenyl isocyanate with amines was explored to afford symmetrical and asymmetrical urea derivatives.

### Optimization of the reaction parameters of synthesis of urea derivatives

The reaction of phenyl isocyanate with aniline was picked up as the model reaction and its effectiveness was checked in four choline chloride based-DES (Table 7, entries 1–4). The results of this study demonstrated that the DES made from ChCl and ZnCl_2_ ([ChCl][ZnCl]_2_) exhibit the highest effectiveness (Table 7, entry 3). To highlight the role of DES, the reaction was repeated separately in the absence of DES and also ZnCl_2_. These experiments resulted in 80% and 86% respectively, which are less (Table 7, entries 5 and 6) than the effectiveness of the reaction in the DES. The outcomes of performing the reaction in the DESs made from the different ratios of ChCl and ZnCl_2_ (Table 7, entries 3, 9, and 10) showed that the best result is obtained when this ratio is 1:2, [ChCl][ZnCl_2_]_2_, (Table 7, entry 3). The exploring of the effect of the molar ratio of phenyl isocyanate to aniline in the model reaction (Table 7, entries 3, 11, and 12) makes clear that the best output was achieved when a molar ratio of 1:1 of phenyl isocyanate to aniline was utilized (Table 7, entry 3). The dependency of the reaction efficiency to temperature was checked and the highest efficiency was observed when the reaction was carried out at 60 °C (Table 7, entries 13 and 14). Lastly, the evaluation of reaction time showed that the model reaction is completed after 8 min (Table 7, entries 15–17). It was found that the optimized conditions, which are utilized in the preparation of *N*-phenylurea derivatives, are: phenyl isocyanate (1 mmol), aniline derivative (1 mmol), 3 mL(6 mmol) [ChCl][ZnCl_2_]_2_, 60 °C, 8 min (Table 7, entry 3) (“Supplementary Information”).

**Table 7:** Optimization of reaction parameters of one-pot synthesis of *N*-phenylurea derivatives.


Entry	Molar ratio A:B	DES	DES (mmol)	Molar mass of DES (g mol^−1^)^a^	Temperature (°C)	Time (min)	Yield (%)^b^
1	1:1	[ChCl][Urea]_2_	6	86.58	60	8	67
2	1:1	[ChCl][Glycerol]_2_	6	107.93	60	8	78
3	1:1	[ChCl][ZnCl_2_]_2_	6	137.41	60	8	93
4	1:1	[ChCl][SnCl_2_]_2_	6	154.67	60	8	91
5	1:1	–	–	–	60	8	80
6	1:1	ZnCl_2_	–	–	60	8	86
7	1:1	[ChCl][ZnCl_2_]_2_	4	137.41	60	8	91
8	1:1	[ChCl][ZnCl_2_]_2_	8	137.41	60	8	93
9	1:1	[ChCl][ZnCl_2_]	6	137.62	60	8	88
10	1:1	[ChCl]_2_[ZnCl_2_]	6	137.12	60	8	86
11	1:2	[ChCl][ZnCl_2_]_2_	6	137.41	60	8	85
12	2:1	[ChCl][ZnCl_2_]_2_	6	137.41	60	8	83
13	1:1	[ChCl][ZnCl_2_]_2_	6	137.41	40	8	88
14	1:1	[ChCl][ZnCl_2_]_2_	6	137.41	80	8	93
15	1:1	[ChCl][ZnCl_2_]_2_	6	137.41	60	5	83
16	1:1	[ChCl][ZnCl_2_]_2_	6	137.41	60	10	93
17	1:1	[ChCl][ZnCl_2_]_2_	6	137.41	60	15	93

^a^HBD: Hydrogen bond donor, HBA: Hydrogen bond acceptor. The molecular mass (*M*_DES_) of DES is measured from Eq: ${M}_{{\text{DES}}}=\frac{{x}_{{\text{HBA}}}*{M}_{{\text{HBA}}}+{x}_{{\text{HBD}}}*{M}_{{\text{HBD}}}}{{x}_{{\text{HBA}}}+{x}_{{\text{HBD}}}}$, where M_DES_ is the molecular mass of DES in g.mol^−1^, χ_HBA_ and x_HBD_. are the mole ratio of the HBA and HBD respectively; M_HBD_ and M_HBA_ are the molecular mass of the HBD and HBA in g mol^−1^.

^b^Isolated yield.

After optimizing the factors of reaction, the reaction of phenyl isocyanate with a variety of aromatic and aliphatic amines was assessed and the related information was summarized in Table 8. This work has shown that both primary and secondary aliphatic and aromatic amines can provide the favorable results (Table 8, entries C28–C38). It is also mentioned that aromatic amines with the electron-withdrawing group can also afford the desired products satisfactorily (Table 8, entries C29 and C30). It was observed that the efficiency of the desired product was extremely low and less than 10% when diphenylamine was utilized as a starting material. Additionally, investigations into aliphatic amines were done and the outcomes are presented in Table 8 (Entries C34–C38).

**Table 8:** Synthesis of unsymmetrical *N*-phenylurea derivatives in [ChCl][ZnCl_2_]_2_ as a solvent/catalyst system.


Substrate	Substrate	Product	Time (min)	Yield (%)^a^	M.p. °C (Lit)
			8	93	250 (250–251)^103^
			35	76	210 (199–202)^104^
			35	78	209 (204–206)^105^
			15	88	235 (233–237)^106^
			15	89	237 (230–235)^106^
			20	85	99–101 (93–94)^107^
			5	97	152 (146–147)^108^
			5	95	99–101 (100–101)^104^
			10	92	130 (129–131)^109^
			30	90	171–172 (168–171)^110^
			30	83	163 (156–158)^111^

Reaction conditions: Amine or amide derivatives (1.0 mmol), phenyl isocyanate (1.0 mmol) and 3 mL of [ChCl][ZnCl_2_]_2_ as a solvent/catalyst.

^a^Isolated yield.

**Figure 6:**
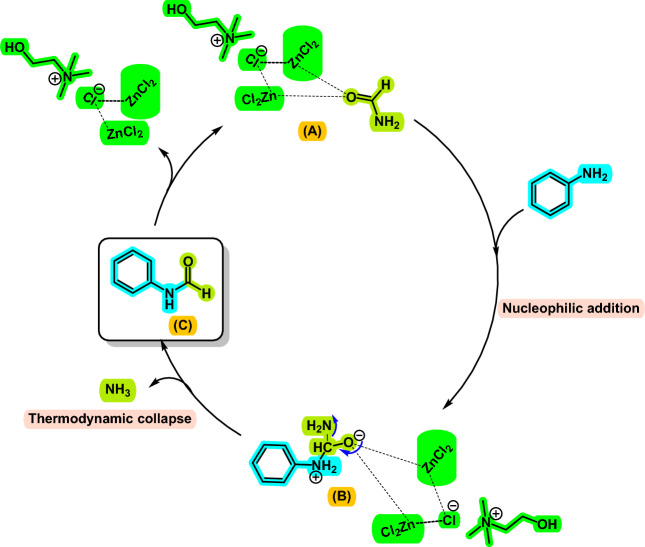
The proposed mechanism for transamidation of carboxamides by [ChCl][ZnCl_2_]_2_.

For interpretation of the function of [ChCl][ZnCl_2_]_2_ in the above-mentioned reactions, a plausible mechanism is illustrated in Fig. 6 for the preparation of formanilide through the transamidation reaction according to the literature and the obtained result. Initially, the intermediate **B** is formed through the nucleophilic addition of aniline on the activated carbonyl group of formamide. This intermediate undergoes proton exchange and then loses ammonia with the aid of DES to produce the desired product **C** and regenerates the DES.

### Recovery of DES ([ChCl][ZnCl_2_]_2_) as a solvent/catalyst system

**Figure 7:**
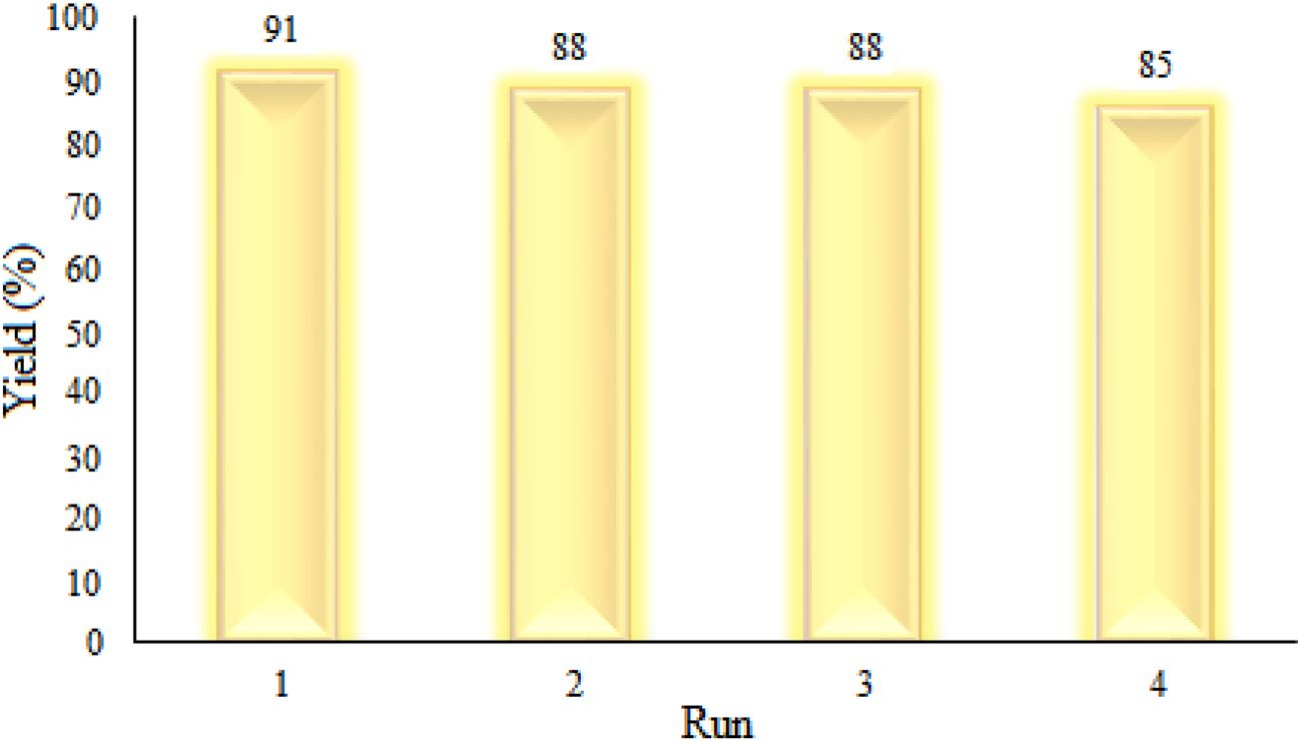
Evaluation of recyclability and reusability of [ChCl][ZnCl_2_]_2_ in the transamidation reaction formamide.

The scientific community has recently focused on the recyclability and reusability of catalysts, which are significant characteristics of catalysts^112,113^. Recycling and reusing catalysts have a positive economic and environmental impact, especially when applied to industrial processes^114^. Therefore, the recyclability of [ChCl][ZnCl_2_]_2_ as a solvent/catalyst system was checked in the transamidation reaction of formamide with aniline. Following the completion of the reaction, the reaction's crude was diluted with water (10 mL) and extracted with ethyl acetate (2 × 5 mL). Then, the aqueous layer was concentrated by evaporation of water at 70 °C under vacuum conditions for 40 min. The dried DES was reused in a subsequent run of the model reaction. As demonstrated in Fig. 7, there was a small decline in the catalytic activity of recycled [ChCl][ZnCl_2_]_2_ after four consecutive cycles of catalyst reuse.

## Conclusions

In summary, we have described a novel and innovative, environmentally friendly, unique, distinct, and efficient process for the one-pot *N*-formylation and *N*-acetylation of primary anilines. Remarkably, the current method includes noteworthy features such as (i) using formamide, formic acid and acetic acid (as a natural material), which are fairly inexpensive sources of carbonyl, (ii) application of the secure and environmentally benign solvent/catalyst system ([ChCl][ZnCl_2_]_2_), which is easily formed via an atom economical procedure, (iii) convenient and straightforward purification and separation process, (iv) good to excellent yield of products, and (v) having potential of scale up, (vi) reusability the DES employed at least for four times. This solvent/catalyst media is also capable to promote efficiently the reaction of phenyl isocyanate with amines to provide unsymmetrical ureas.

### Supplementary Information

Supplementary Information.

## Data Availability

Data is provided within the manuscript or supplementary information files.
