# Supplementary material for: Direct additive-free N-formylation and N-acylation of anilines and synthesis of urea derivatives using green, efficient, and reusable deep eutectic solvent ([ChCl][ZnCl2]2)
Source: Sci Rep. 2024 Mar 26;14:7206. doi: 10.1038/s41598-024-57608-8 (PMC10966014; doi:10.1038/s41598-024-57608-8)
Supplement: Supplementary file 1 — Supplementary Information. [file 41598_2024_57608_MOESM1_ESM.docx]

**Supplementary information**

**Direct Additive-Free N-Formylation and N-Acylation of Anilines and Synthesis of Urea Derivatives Using Green, Efficient, and Reusable Deep Eutectic Solvent ([ChCl][ZnCl_2_]_2_)**

**Fatemeh Abbasi ^1^ ● Ali Reza Sardarian^1^**

Scientific Reports

🖂 Ali Reza Sardarian

sardarian@shirazu.ac.ir

^1^ Department of Chemistry, College of Sciences, Shiraz University, 71946 84795 Shiraz, Iran

| **Table of Contents** | **Page** |
| --- | --- |
| **Fig. S1:** The FT-IR spectrum of (**C1**) (Table 2, entry 1) | 14 |
| **Fig. S2:** The ^1^H NMR spectrum of (**C1**) (Table 2, entry 1) | 14 |
| **Fig. S3:** The ^13^C NMR spectrum of (**C1**) (Table 2, entry 1) | 15 |
| **Fig. S4:** The FT-IR spectrum of (**C2**) (Table 2, entry 2) | 15 |
| **Fig. S5**: The ^1^H NMR spectrum of (**C2**) (Table 2, entry 2) | 16 |
| **Fig. S6:** The ^13^C NMR spectrum of (**C2**) (Table 2, entry 2) | 16 |
| **Fig. S7:** The FT-IR spectrum of (**C3**) (Table 2, entry 3) | 17 |
| **Fig. S8:** The ^1^H NMR spectrum of (**C3**) (Table 2, entry 3) | 17 |
| **Fig. S9:** The ^13^C NMR spectrum of (**C3**) (Table 2, entry 3) | 18 |
| **Fig. S10:** The FT-IR spectrum of (**C4**) (Table 2, entry 4) | 18 |
| **Fig. S11:** The ^1^H NMR spectrum of (**C4**) (Table 2, entry 4) | 19 |
| **Fig. S12:** The ^13^C NMR spectrum of (**C4**) (Table 2, entry 4) | 19 |
| **Fig. S13:** The FT-IR spectrum of (**C5**) (Table 2, entry 5) | 20 |
| **Fig. S14:** The ^1^H NMR spectrum of (**C5**) (Table 2, entry 5) | 20 |
| **Fig. S15:** The ^13^C NMR spectrum of (**C5**) (Table 2, entry 5) | 21 |
| **Fig. S16:** The FT-IR spectrum of (**C6**) (Table 2, entry 6) | 21 |
| **Fig. S17:** The ^1^H NMR spectrum of (**C6**) (Table 2, entry 6) | 22 |
| **Fig. S18:** The ^13^C NMR spectrum of (**C6**) (Table 2, entry 6) | 22 |
| **Fig. S19:** The FT-IR spectrum of (**C7**) (Table 2, entry 7) | 23 |
| **Fig. S20:** The ^1^H NMR spectrum of (**C7**) (Table 2, entry 7) | 23 |
| **Fig. S21:** The ^13^C NMR spectrum of (**C7**) (Table 2, entry 7) | 24 |
| **Fig. S22:** The FT-IR spectrum of (**C8**) (Table 2, entry 8) | 24 |
| **Fig. S23:** The ^1^H NMR spectrum of (**C8**) (Table 2, entry 8 ) | 25 |
| **Fig. S24:** The ^13^C NMR spectrum of (**C8**) (Table 2, entry 8) | 25 |
| **Fig. S25:** The FT-IR spectrum of (**C9**) (Table 2, entry 9) | 26 |
| **Fig. S26:** The ^1^H NMR spectrum of (**C9**) (Table 2, entry 9) | 26 |
| **Fig. S27:** The ^13^C NMR spectrum of (**C9**) (Table 2, entry 9) | 27 |
| **Fig. S28:** The FT-IR spectrum of (**C10**) (Table 2, entry 10) | 27 |
| **Fig. S29:** The ^1^H NMR spectrum of (**C10**) (Table 2, entry 10) | 28 |
| **Fig. S30:** The ^13^C NMR spectrum of (**C10**) (Table 2, entry 10) | 28 |
| **Fig. S31:** The FT-IR spectrum of (**C11**) (Table 2, entry 11) | 29 |
| **Fig. S32:** The ^1^H NMR spectrum of (**C11**) (Table 2, entry 11) | 29 |
| **Fig. S33:** The ^13^C NMR spectrum of (**C11**) (Table 2, entry 11) | 30 |
| **Fig. S34:** The FT-IR spectrum of (**C12**) (Table 2, entry 12) | 30 |
| **Fig. S35:** The ^1^H NMR spectrum of (**C12**) (Table 2, entry 12) | 31 |
| **Fig. S36:** The ^13^C NMR spectrum of (**C12**) (Table 2, entry 12) | 31 |
| **Fig. S37:** The FT-IR spectrum of (**C13**) (Table 2, entry 13) | 32 |
| **Fig. S38:** The ^1^H NMR spectrum of (**C13**) (Table 2, entry 13) | 32 |
| **Fig. S39:** The ^13^C NMR spectrum of (**C13**) (Table 2, entry 13) | 33 |
| **Fig. S40:** The FT-IR spectrum of (**C14**) (Table 4, entry 18) | 33 |
| **Fig. S41:** The ^1^H NMR spectrum of (**C14**) (Table 4, entry 18) | 34 |
| **Fig. S42:** The ^13^C NMR spectrum of (**C14**) (Table 4, entry 18) | 34 |
| **Fig. S43:** The FT-IR spectrum of (**C15**) (Table 4, entry 19) | 35 |
| **Fig. S44:** The ^1^H NMR spectrum of (**C15**) (Table 4, entry 19) | 35 |
| **Fig. S45:** The ^13^C NMR spectrum of (**C15**) (Table 4, entry 19) | 36 |
| **Fig. S46:** The FT-IR spectrum of (**C16**) (Table 4, entry 21) | 36 |
| **Fig. S47:** The ^1^H NMR spectrum of (**C16**) (Table 4, entry 21) | 37 |
| **Fig. S48:** The ^13^C NMR spectrum of (**C16**) (Table 4, entry 21) | 37 |
| **Fig. S49:** The FT-IR spectrum of (**C17**) (Table 4, entry 23) | 38 |
| **Fig. S50:** The ^1^H NMR spectrum of (**C17**) (Table 4, entry 23) | 38 |
| **Fig. S51:** The ^13^C NMR spectrum of (**C17**) (Table 4, entry 23) | 39 |
| **Fig. S52:** The FT-IR spectrum of (**C18**) (Table 4, entry 24) | 39 |
| **Fig. S53:** The ^1^H NMR spectrum of (**C18**) (Table 4, entry 24) | 40 |
| **Fig. S54:** The ^13^C NMR spectrum of (**C18**) (Table 4, entry 24) | 40 |
| **Fig. S55:** The FT-IR spectrum of (**C19**) (Table 4, entry 25) | 41 |
| **Fig. S56:** The ^1^H NMR spectrum of (**C19**) (Table 4, entry 25) | 41 |
| **Fig. S57:** The ^13^C NMR spectrum of (**C19**) (Table 4, entry 25) | 42 |
| **Fig. S58:** The FT-IR spectrum of (**C20**) (Table 4, entry 26) | 42 |
| **Fig. S59:** The ^1^H NMR spectrum of (**C20**) (Table 4, entry 26) | 43 |
| **Fig. S60:** The ^13^C NMR spectrum of (**C20**) (Table 4, entry 26) | 43 |
| **Fig. S61:** The FT-IR spectrum of (**C21**) (Table 7, entry 28) | 44 |
| **Fig. S62:** The ^1^H NMR spectrum of (**C21**) (Table 7, entry 28) | 44 |
| **Fig. S63:** The ^13^C NMR spectrum of (**C21**) (Table 7, entry 28) | 45 |
| **Fig. S64:** The FT-IR spectrum of (**C22**) (Table 7, entry 29) | 45 |
| **Fig. S65:** The ^1^H NMR spectrum of (**C22**) (Table 7, entry 29) | 46 |
| **Fig. S66:** The ^13^C NMR spectrum of (**C22**) (Table 7, entry 29) | 46 |
| **Fig. S67:** The FT-IR spectrum of (**C23**) (Table 7, entry 30) | 47 |
| **Fig. S68:** The ^1^H NMR spectrum of (**C23**) (Table 7, entry 30) | 47 |
| **Fig. S69:** The ^13^C NMR spectrum of (**C23**) (Table 7, entry 30) | 48 |
| **Fig. S70:** The FT-IR spectrum of (**C24**) (Table 7, entry 31) | 48 |
| **Fig. S71:** The ^1^H NMR spectrum of (**C24**) (Table 7, entry 31) | 49 |
| **Fig. S72:** The ^13^C NMR spectrum of (**C24**) (Table 7, entry 31) | 49 |
| **Fig. S73:** The FT-IR spectrum of (**C25**) (Table 7, entry 32) | 50 |
| **Fig. S74:** The ^1^H NMR spectrum of (**C25**) (Table 7, entry 32) | 50 |
| **Fig. S75:** The ^13^C NMR spectrum of (**C25**) (Table 7, entry 32) | 51 |
| **Fig. S76:** The FT-IR spectrum of (**C26**) (Table 7, entry 33) | 51 |
| **Fig. S77:** The ^1^H NMR spectrum of (**C26**) (Table 7, entry 33) | 52 |
| **Fig. S78:** The ^13^C NMR spectrum of (**C26**) (Table 7, entry 33) | 52 |
| **Fig. S79:** The FT-IR spectrum of (**C27**) (Table 7, entry 34) | 53 |
| **Fig. S80:** The ^1^H NMR spectrum of (**C27**) (Table 7, entry 34) | 53 |
| **Fig. S81:** The ^13^C NMR spectrum of (**C27**) (Table 7, entry 34) | 54 |
| **Fig. S82:** The FT-IR spectrum of (**C28**) (Table 7, entry 35) | 54 |
| **Fig. S83:** The ^1^H NMR spectrum of (**C28**) (Table 7, entry 35) | 55 |
| **Fig. S84:** The ^13^C NMR spectrum of (**C28**) (Table 7, entry 35) | 55 |
| **Fig. S85:** The FT-IR spectrum of (**C29**) (Table 7, entry 36) | 56 |
| **Fig. S86:** The ^1^H NMR spectrum of (**C29**) (Table 7, entry 36) | 56 |
| **Fig. S87:** The ^13^C NMR spectrum of (**C29**) (Table 7, entry 36) | 57 |
| **Fig. S88:** The FT-IR spectrum of (**C30**) (Table 7, entry 37) | 57 |
| **Fig. S89:** The ^1^H NMR spectrum of (**C30**) (Table 7, entry 37) | 58 |
| **Fig. S90:** The ^13^C NMR spectrum of (**C30**) (Table 7, entry 37) | 58 |
| **Fig. S91:** The FT-IR spectrum of (**C31**) (Table 7, entry 38) | 59 |
| **Fig. S92:** The ^1^H NMR spectrum of (**C31**) (Table 7, entry 38) | 59 |
| **Fig. S93:** The ^13^C NMR spectrum of (**C31**) (Table 7, entry 38) | 60 |
| **Fig. S94:** The FT-IR spectrum of DES and starting materials | 60 |

***N*-(4-Chlorophenyl) formamide** (**C1**)

White solid, yield: 88%, mp: 103-105 ^°^C (Lit., 104-105 ^°^C) [1], FT-IR (KBr) ῡ(cm^-1^): 3287, 3260, 3193, 3122, 3102, 3060, 2999, 2935, 2894, 2789, 2370, 1896, 1688, 1671, 1608, 1559, 1542, 1508, 1491, 1408, 1398, 1311, 1293, 1253, 1088, 1012, 871, 820, 783, 768, 611, 518, 420 cm^-1^. ^1^H NMR (400 MHz, DMSO-*d_6_*) δ (ppm): 7.37 (dd, J=2, 6.8 Hz, 2H), 7.62 (dd, J=2.4, 6.8 Hz, 2H), 8.30 (s, 1H), 10.34 (s, 1H). ^13^C NMR (100 MHz, DMSO-*d_6_*) δ (ppm): 118.9, 120.6, 127.1, 128.7, 129.1, 137.1, 159.6. Anal Calcd for: C_7_H_6_ClNO: C, 54.04; H, 3.89; N, 9.00%. Found: C, 54.38; H, 4.09; N, 8.87%.

***N*-(2-Cyanophenyl) formamide** (**C2**)

Yellow solid, yield: 72%, mp: 122-124 ^°^C (Lit., 121-122 ^°^C) [2], FT-IR (KBr) ῡ(cm^-1^): 3491, 3258, 3119, 2909, 2220, 1980, 1711, 1669, 1589, 1542, 1473, 1450, 1403, 1300, 1259, 1151, 864, 754, 709 cm^-1^. ^1^H NMR (400 MHz, DMSO-*d_6_*) δ (ppm): 7.31-7.37 (m, 1H), 7.68-7.73 (m, 1H), 7.81-7.86 (d, J=7.2, 1H), 7.92-7.97 (d, J=7.6, 1H), 8.37 (s, 1H), 10.39 (s, 1H). ^13^C NMR (100 MHz, DMSO-*d_6_*) δ (ppm): 104.4, 116.4, 123.4, 125.2, 133.3, 134.0, 139.3. 160.5. Anal Calcd for: C_8_H_6_N_2_O: C, 65.75; H, 4.14; N, 19.17%. Found: C, 65.83; H, 4.11; N, 18.96%.

***N*-(4-Ethylphenyl) formamide** (**C3**)

White solid, yield: 89%, mp: 44-46 ^°^C (Lit., 50-52 ^°^C) [3], FT-IR (KBr) ῡ(cm^-1^): 3257, 3120, 3044, 2964, 2931, 2873, 1686, 1611, 1353, 1521, 1412, 1294, 1123, 1074, 963, 833, 745, 601, 541, 477 cm^-1^ . ^1^H NMR (400 MHz, DMSO-*d_6_*) δ (ppm): 1.11-1.16 (t, J=7.6, 3H), 2.53 (q, 2H), 7.11-7.13 (m, 1H), 7.14-7.15 (m, 1H), 7.53 (dd, J=1.6, 6.8 Hz, 2H), 8.27 (s, 1H), 10.11 (s, 1H). ^13^C NMR (100 MHz, DMSO-*d_6_*) δ (ppm): 15.6, 27.6, 117.7, 119.1, 127.9, 128.5, 135.9, 138.9, 159.2. Anal Calcd for: C_9_H_11_NO: C, 72.46; H, 7.43; N, 9.39%. Found: C, 72.61; H, 7.51; N, 9.11%.

***N*-(4-Isopropylphenyl) formamide** (**C4**)

Brown oil, yield: 85%, [4], FT-IR (KBr) ῡ(cm^-1^): 3257, 3120, 3049, 2961, 2872, 1686, 1611, 1522, 1412, 1313, 1256, 1187, 1139, 1055, 1018, 832, 790, 599, 552, 454 cm^-1^. ^1^H NMR (400 MHz, DMSO-*d_6_*) δ (ppm): 1.16 (d, J= 8 Hz, 6H), 2.75-2.85 (hept, 1H), 7.14-7.18 (m, 2H), 7.54 (dd, J=1.6, 6.4 Hz, 2H), 8.28 (s, 1H), 10.12 (s, 1H). ^13^C NMR (100 MHz, DMSO-*d_6_*) δ (ppm): 23.8, 32.8, 117.7, 119.1, 126.4, 127.0, 136.0, 143.5, 159.2. Anal Calcd for: C_10_H_13_NO: C, 73.59; H, 8.03; N, 8.58%. Found: C, 73.81; H, 8.26; N, 8.42%.

***N*-(2,4-Dichlorophenyl) formamide** (**C5**)

Yellow solid, yield: 83%, mp: 154-156 ^°^C (Lit., 154-157 ^°^C) [5], FT-IR (KBr) ῡ(cm^-1^): 3464, 3243, 3160, 3089, 3027, 2898, 2777, 1882, 1697, 1665, 1601, 1585, 1426, 1400, 1383, 1334, 1298, 1159, 1140, 1105, 1051, 868, 832, 817, 750, 701, 541 cm^-1^. ^1^H NMR (400 MHz, DMSO-*d_6_*) δ (ppm): 7.40-7.44 (m, 1H), 7.65-7.69 (m, 1H), 8.13-8.17 (m, 1H), 8.36 (s, 1H), 9.99 (s, 1H). ^13^C NMR (100 MHz, DMSO-*d_6_*) δ (ppm): 124.0, 124.3, 127.6, 128.3, 128.8, 133.4, 160.4. Anal Calcd for: C_7_H_5_Cl_2_NO: C, 44.25; H, 2.65; N, 7.37%. Found: C, 43.97; H, 2.76; N, 7.21%.

***N*-(3-Nitrophenyl) formamide** (**C6**)

Yellow solid, yield: 68%, mp: 136-138 ^°^C (Lit., 134-136 ^°^C) [6], FT-IR (KBr) ῡ(cm^-1^): 3427, 3264, 3126, 3093, 2902, 2409, 2329, 1667, 1531, 1432, 1397, 1350, 1274, 1171, 1155, 1085, 1071, 938, 887, 846, 795, 786, 735, 667, 511 cm^-1^. ^1^H NMR (400 MHz, DMSO-*d_6_*) δ (ppm): 7.6-7.65 (m, 1H), 7.87-7.96 (m, 2H), 8.39 (s, 1H), 8.61-8.63 (m, 1H), 10.69 (s, 1H). ^13^C NMR (100 MHz, DMSO-*d_6_*) δ (ppm): 113.3, 118.1, 125.0, 130.3, 139.2, 147.9, 160.3. Anal Calcd for: C_7_H_6_N_2_O_3_: C, 50.61; H, 3.64; N, 16.86%. Found: C, 51.02; H, 3.73; N, 16.69%.

***N*-(2-Nitrophenyl) formamide** (**C7**)

Yellow solid, yield: 66%, mp: 173-175 ^°^C (Lit., 174-175 ^°^C) [7], FT-IR (KBr) ῡ(cm^-1^): 3431, 3286, 2926, 2852, 2379, 1689, 1610, 1508, 1409, 1336, 1309, 1272, 1223, 1153, 1074, 866, 779, 737, 523 cm^-1^. ^1^H NMR (400 MHz, DMSO-*d_6_*) δ (ppm): 7.34-7.39 (m, 1H), 7.71-7.76 (m, 1H), 8.03-8.10 (m, 2H), 8.40 (s, 1H), 10.58 (s, 1H). ^13^C NMR (100 MHz, DMSO-*d_6_*) δ (ppm): 123.9, 124.8, 125.2, 131.0, 134.6, 140.0, 160.7. Anal Calcd for: C_7_H_6_N_2_O_3_: C, 50.61; H, 3.64; N, 16.86%. Found: C, 49.97; H, 3.36; N, 16.74%.

***N*-(2-Methyl-5-nitrophenyl) formamide** (**C8**)

Yellow solid, yield: 72%, mp: 178-180 ^°^C, FT-IR (KBr) ῡ(cm^-1^): 3274, 3128, 2902, 2855, 2367, 1897, 1675, 1604, 1542, 1526, 1423, 1399, 1382, 1346, 1320, 1259, 1130, 1074 cm^-1^. ^1^H NMR (400 MHz, DMSO-*d_6_*) δ (ppm): 2.36 (s, 1H), 7.49-7.53 (d,J=8.4, 1H), 7.89-7.94 (m, 1H), 8.39-8.43 (m, 1H), 8.85 (s, 1H), 9.96 (s, 1H). ^13^C NMR (100 MHz, DMSO-*d_6_*) δ (ppm): 18.0, 115.7, 118.7, 131.3, 136.3, 136.5, 145.7, 160.5. Anal Calcd for: C_8_H_8_N_2_O_3_: C, 53.33; H, 4.48; N, 15.55%. Found: C, 53.70; H, 4.29; N, 15.48%.

***N*-Phenyl formamide** (**C9**)

White solid, yield: 91%, mp: 46-48 ^°^C (Lit., 47-49 ^°^C) [8], FT-IR (KBr) ῡ(cm^-1^): 3237, 3184, 3130, 3053, 3010, 2922, 2896, 1684, 1673, 1605, 1545, 1493, 1486, 1441, 1412, 1320, 1309, 1255, 1221, 1176, 1149, 1076, 1036, 898, 839, 750, 734, 691, 660, 518, 505, 468 cm^-1^. ^1^H NMR (400 MHz, DMSO-*d_6_*) δ (ppm): 7.04-7.10 (m, 1H), 7.19-7.24 (m, 1H), 7.28-7.33 (m, 2H), 7.63-7.66 (m, 1H), 8.32 (s, 1H), 10.21 (s, 1H). ^13^C NMR (100 MHz, DMSO-*d_6_*) δ (ppm): 117.5, 119.1, 123.6, 128.7, 129.3, 138.2, 159.5. Anal Calcd for: C_7_H_7_NO: C, 69.41; H, 5.82; N, 11.56%. Found: C, 68.91; H, 5.19; N, 11.39%.

***N*-(4-Cyanophenyl) formamide** (**C10**)

White solid, yield: 74%, mp: 183-185 ^°^C (Lit., 180-183 ^°^C) [9], FT-IR (KBr) ῡ(cm^-1^): 3329, 3129, 3076, 2961, 2930, 2361, 2224, 1709, 1609, 1522, 1496, 1419, 1308, 1283, 1217, 1183, 1136, 1071, 1002, 885, 837, 693, 565, 548, 466, 419 cm^-1^. ^1^H NMR (400 MHz, DMSO-*d_6_*) δ (ppm): 7.76 (dd, J=2, 6.8 Hz, 2H), 7.80 (dd, J=2, 6.8 Hz, 2H), 8.37 (s, 1H), 10.66 (s, 1H), 7.83-7.87 (m, 2H), 9.99 (s, 1H). ^13^C NMR (100 MHz, DMSO-*d_6_*) δ (ppm): 105.3, 117.0, 118.9, 119.2, 133.4, 133.7, 142.2, 160.4. Anal Calcd for: C_8_H_6_N_2_O: C, 65.75; H, 4.14; N, 19.17%. Found: C, 66.13; H, 4.29; N, 19.03%.

***N*-*o*-Tolyl formamide** (**C11**)

White solid, yield: 81%, mp: 56-58 ^°^C (Lit., 57-58 ^°^C) [1], FT-IR (KBr) ῡ(cm^-1^): 3438, 3205, 2907, 2361, 1681, 1466, 1396, 1283, 1042, 995, 882, 791, 754, 716, 644, 547, 482 cm^-1^. ^1^H NMR (400 MHz, CDCl_3_) δ (ppm): 2.23 (s, 3H), 7.02-7.08 (m, 1H), 7.15-7.24 (m, 3H), 8.32 (s, 1H), 9.58 (s, 1H). ^13^C NMR (100 MHz, CDCl_3_) δ (ppm): 17.7, 122.7, 124.5, 125.2, 126.0, 130.3, 135.6, 159.7. Anal Calcd for: C_8_H_9_NO: C, 71.09; H, 6.71; N, 10.36%. Found: C, 70.81; H, 6.86; N, 10.16%.

***N*-(Naphthalen-1-yl) formamide** (**C12**)

 White solid, yield: 68%, mp: 109-110 ^°^C (Lit., 109-110 ^°^C) [10] FT-IR (KBr) ῡ(cm^-1^): 3413, 3234, 2876, 2037, 1639, 1618, 1385, 1269, 1153, 790, 771, 623, 474 cm^-1^. ^1^H NMR (400 MHz, DMSO-*d_6_*) δ (ppm): 7.47-7.53 (m, 1H), 7.55-7.61 (m, 2H), 7.92-8.05 (m, 2H), 8.14-8.17 (m, 1H), 8.15 (s, 1H), 10.34 (s, 1H). ^13^C NMR (100 MHz, DMSO-*d_6_*) δ (ppm): 118.1, 119.3, 121.7, 122.4, 124.7, 125.4, 126.0, 128.2, 132.5, 133.6, 160.2. Anal Calcd for: C_11_H_9_NO: C, 77.17; H, 5.30; N, 8.18%. Found: C, 77.28; H, 5.21; N, 7.93%.

**N-*m*-Tolyl formamide** (**C13**)

 White solid, yield: 87%, mp: 160-162 ^°^C (Lit., 182-187 ^°^C) [9], FT-IR (KBr) ῡ(cm^-1^): 3476, 3414, 3240, 3078, 1681, 1615, 1596, 1550, 1491, 1455, 1403, 1298, 1202, 1169, 779, 691, 621 cm^-1^. ^1^H NMR (400 MHz, DMSO-*d_6_*) δ (ppm): 2.26 (s, 3H), 6.84-6.89 (m, 1H), 7.15-7.20 (m, 1H), 7.43-7.51 (m, 1H), 8.33 (S, 1H), 10.12 (s, 1H). ^13^C NMR (100 MHz, DMSO-*d_6_*) δ (ppm): 20.9, 118.0, 119.6, 124.2, 128.5, 138.1, 138.7, 159.4. Anal Calcd for: C_8_H_9_NO: C, 71.09; H, 6.71; N, 10.36%. Found: C, 70.87; H, 6.83; N, 10.19%.

 ***N*-Phenyl acetamide** (**C14**)

White solid, yield: 81%, mp: 115 ^°^C (Lit., 113-115 ^°^C), [11] FT-IR (KBr) ῡ(cm^-1^): 3441, 3294, 3261, 3136, 2926, 2854, 1658, 1600, 1437, 1369, 1323, 1264, 1041, 1014, 961, 907, 754, 694, 606 cm^-1^. ^1^H NMR (400 MHz, DMSO-*d_6_*) δ (ppm): 2.07 (s, 3H), 7.10-7.15 (m, 1H), 7.30-7.35 (m, 2H), 7.52-7.54 (m, 2H), 9.96 (s, 1H). ^13^C NMR (100 MHz, DMSO-*d_6_*) δ (ppm): 24.4, 120.1, 124.4, 128.9, 137.8, 168.8. Anal Calcd for: C_8_H_9_NO: C, 71.09; H, 6.71; N, 10.36%. Found: C, 71.38; H, 6.84; N, 10.19%.

*****N*-([1,1'-Biphenyl]-4-yl) acetamide** (**C15**)

White solid, yield: 67%, mp: 171 ^°^C (Lit., 169 ^°^C), [12] FT-IR (KBr) ῡ(cm^-1^): 3435, 3305, 3118, 2925, 2854, 2374, 1658, 1541, 1376, 1320, 1299, 1260, 1190, 1117, 967, 834, 761, 695, 599, 551, 494 cm^-1^. ^1^H NMR (400 MHz, DMSO-*d_6_*) δ (ppm): 2.11 (s, 3H), 7.33-7.38 (m, 1H), 7.45-7.49 (m, 2H), 7.63-7.65(m, 2H), 7.66-7.73(m, 4H), 10.09 (s, 1H). ^13^C NMR (100 MHz, DMSO-*d_6_*) δ (ppm): 24.0, 119.2, 126.1, 126.9, 128.8, 134.5, 138.7, 139.7, 168.2. Anal Calcd for: C_14_H_13_NO: C, 79.59; H, 6.20; N, 6.63%. Found: C, 79.48; H, 6.34; N, 6.48%.

***N*-(*m*-Tolyl) acetamide** (**C16**)

White solid, yield: 74%, mp: 66 ^°^C (Lit., 65.5-67.1 ^°^C), [13] FT-IR (KBr) ῡ(cm^-1^): 3292, 3146, 2919, 2360, 1664, 1615, 1571, 1491, 1407, 1367, 1262, 1168, 1038, 1006, 877, 784, 754, 693, 609 cm^-1^. ^1^H NMR (400 MHz, DMSO-*d_6_*) δ (ppm): 2.03 (s, 3H), 2.25 (s, 3H), 6.83-6.85 (m, 1H), 7.14-7.18 (m, 1H), 7.35-7.38(m, 1H), 7.42(s, 1H), 9.87 (s, 1H). ^13^C NMR (100 MHz, DMSO-*d_6_*) δ (ppm): 21.16, 23.9, 116.1, 119.4, 123.6, 128.4, 137.7, 139.2, 168.1. Anal Calcd for: C_9_H_11_NO: C, 72.46; H, 7.43; N, 9.39%. Found: C, 72.59; H, 7.61; N, 8.98%.

***N*-(4-Chlorophenyl) acetamide** (**C17**)

White solid, yield: 77%, mp: 184-186 ^°^C (Lit., 187-188 ^°^C) [14], FT-IR (KBr) ῡ(cm^-1^): 3461, 3305, 3193, 3128, 2926, 2852, 2369, 1901, 1666, 1609, 1492, 1391, 1371, 1314, 1260, 1170, 1090, 1009, 969, 750, 708, 607, 506, 450 cm^-1^. ^1^H NMR (400 MHz, DMSO-*d_6_*) δ (ppm): 2.05 (s, 3H), 7.32-7.35 (m, 2H), 7.59-7.63 (m, 2H), 10.12 (s, 1H). ^13^C NMR (100 MHz, DMSO-*d_6_*) δ (ppm): 23.9, 120.4, 126.4, 128.5, 138.2, 168.4. Anal Calcd for: C_8_H_8_ClNO: C, 56.65; H, 4.75; N, 8.26%. Found: C, 56.81; H, 4.93; N, 7.97%.

***N*-(2-Nitrophenyl) acetamide** (**C18**)

Yellow solid, yield: 47%, mp: 93 ^°^C (Lit., 91-93 ^°^C) [15], FT-IR (KBr) ῡ(cm^-1^): 3560, 3252, 3060, 2898, 2604, 2195, 2038, 1937, 1816, 1699, 1608, 1553, 1431, 1285, 1234, 1032, 885, 825, 749, 572 cm^-1^. ^1^H NMR (400 MHz, DMSO-*d_6_*) δ (ppm): 2.06 (s, 3H), 7.34-7.38 (m, 1H), 7.58-7.61 (m, 1H), 7.67-7.72 (m, 1H), 10.30 (s, 1H). ^13^C NMR (100 MHz, DMSO-*d_6_*) δ (ppm): 23.7, 125.2, 125.5, 125.7, 131.5, 134.3, 143.0, 168.9. Anal Calcd for: C_8_H_8_N_2_O_3_: C, 53.33; H, 4.48; N, 15.55%. Found: C, 53.48; H, 4.61; N, 15.41%.

***N*-(3-Nitrophenyl) acetamide** (**C19**)

Yellow solid, yield: 56%, mp: 154 ^°^C (Lit., 154-155 ^°^C) [16], FT-IR (KBr) ῡ(cm^-1^): 3484, 3253, 3205, 3205, 3074, 2863, 2574, 2158, 2058, 1957, 1874, 1711, 1637, 1332, 1253, 1068, 868, 568 cm^-1^. ^1^H NMR (400 MHz, DMSO-*d_6_*) δ (ppm): 2.09 (s, 3H), 7.54-7.59 (m, 1H), 7.85-7.88 (m, 2H), 8.60-8.61 (m, 1H), 10.40 (s, 1H). ^13^C NMR (100 MHz, DMSO-*d_6_*) δ (ppm): 24.4, 113.3, 117.9, 125.2, 130.4, 140.8, 148.3, 169.4. Anal Calcd for: C_8_H_8_N_2_O_3_: C, 53.33; H, 4.48; N, 15.55%. Found: C, 53.49; H, 4.57; N, 15.39%.

***N*-(4-Cyanophenyl) acetamide** (**C20**)

White solid, yield: 64%, mp: 208 ^°^C (Lit., 206-208 ^°^C) [17], FT-IR (KBr) ῡ(cm^-1^): 3516, 3432, 3380, 3080, 2996, 2765, 2486, 2155, 2061, 1982, 1787, 1677, 1598, 1472, 1246, 1004, 873, 684, 510 cm^-1^.^1^H NMR (400 MHz, DMSO-*d_6_*) δ (ppm): 2.10 (s, 3H), 7.73-7.75 (m, 2H), 7.79-7.81 (m, 2H), 10.71 (s, 1H). ^13^C NMR (100 MHz, DMSO-*d_6_*) δ (ppm): 24.6, 105.0, 119.3, 119.6, 138.6, 144.0, 169.7, 175.8. Anal Calcd for: C_9_H_8_N_2_O: C, 67.49; H, 5.03; N, 17.49%. Found: C, 67.39; H, 5.19; N, 17.31%.

**1,3-Diphenyl urea** (**C21**)

White solid, yield: 93%, mp: 250 ^°^C (Lit., 250-251 ^°^C) [18], FT-IR (KBr) ῡ(cm^-1^): 3270, 3131, 2239, 1940, 1871, 1648, 1594, 1554, 1496, 1448, 1315, 1232, 1107, 894, 753, 695 cm^-1^. ^1^H NMR (400 MHz, DMSO-*d_6_*) δ (ppm): 6.96-7.00 (m, 2H), 7.27-7.31 (m, 4H), 7.46-7.49 (m, 4H), 8.67 (s, 2H). ^13^C NMR (100 MHz, DMSO-*d_6_*) δ (ppm): 118.6, 122.2, 129.2, 140.1, 153.0. Anal Calcd for: C_13_H_12_N_2_O: C, 73.56; H, 5.70; N, 13.20%. Found: C, 72.11; H, 5.68; N, 13.01%.

**1-(4-Cyanophenyl)-3-phenyl urea** (**C22**)

White solid, yield: 76%, mp: 210 ^°^C (Lit., 199-202 ^°^C) [19], FT-IR (KBr) ῡ(cm^-1^): 3437, 3301, 3041, 2920, 2358, 2227, 1627, 1558, 1311, 1235, 1175, 1053, 901, 849, 792, 749, 667, 552 cm^-1^. ^1^H NMR (400 MHz, DMSO-*d_6_*) δ (ppm): 6.97-7.03 (m, 1H), 7.29-7.32 (m, 2H), 7.46-7.49 (m, 2H), 7.63-7.66 (m, 2H), 7.72-7.74 (m, 2H), 8.85 (s, 1H), 9.19 (s, 1H). ^13^C NMR (100 MHz, DMSO-*d_6_*) δ (ppm): 103.6, 118.4, 119.0, 119.7, 122.8, 129.3, 138.7, 139.6, 144.6, 152.5. Anal Calcd for: C_14_H_11_N_3_O: C, 70.87; H, 4.67; N, 17.71%. Found: C, 71.93; H, 5.33; N, 17.28%.

**1-(3-Nitrophenyl)-3-phenyl urea (C23)**

White solid, yield: 78%, mp: 209 ^°^C (Lit., 204-206 ^°^C) [20], FT-IR (KBr) ῡ(cm^-1^): 3315, 3178, 3090, 1640, 1595, 1556, 1524, 1447, 1350, 1314, 1273, 1236, 1051, 892, 745, 499 cm^-1^. ^1^H NMR (400 MHz, DMSO-*d_6_*) δ (ppm): 6.99-7.03 (m, 1H), 7.28-7.33 (m, 2H), 7.45-7.50 (m, 2H), 7.55-7.59 (m, 1H), 7.70-7.73 (m, 1H), 7.81-7.84 (m, 1H), 8.57-8.58 (m, 1H), 8.84 (s, 1H), 9.21 (s, 1H). ^13^C NMR (100 MHz, DMSO-*d_6_*) δ (ppm): 112.5, 116.7, 119.0, 122.7, 124.7, 129.2, 130.5, 139.6, 141.5, 148.6, 152.8. Anal Calcd for: C_36_H_11_N_3_O_3_: C, 81.05; H, 2.08; N, 7.88%. Found: C, 83.21; H, 2.41; N, 7.41%.

**1-Phenyl-3-(*o*-tolyl) urea** (**C24**)

 White solid, yield: 88%, mp: 236 ^°^C (Lit., 233-237 ^°^C) [21], FT-IR (KBr) ῡ(cm^-1^): 3304, 3037, 1634, 1597, 1556, 1498, 1458, 1445, 1295, 1242, 1188, 1049, 895, 748, 692, 496 cm^-1^. ^1^H NMR (400 MHz, DMSO-*d_6_*) δ (ppm): 2.25 (s, 3H), 6.93-6.99 (m, 2H), 7.13-7.19 (m, 2H), 7.27-7.31 (m, 2H), 7.46-7.49 (m, 2H), 7.84-7.87 (m, 1H), 7.92 (s, 1H), 9.02 (s, 1H). ^13^C NMR (100 MHz, DMSO-*d_6_*) δ (ppm): 18.3, 118.4, 121.4, 122.1, 123.0, 126.6, 127.9, 129.2, 130.6, 137.8, 140.3, 153.1. Anal Calcd for: C_14_H_14_N_2_O: C, 74.31; H, 6.24; N, 12.38%. Found: C, 76.01; H, 6.63; N, 11.98%.

**1-Phenyl-3-(*m*-tolyl) urea** (**C25**)

 White solid, yield: 89%, mp: 237 ^°^C (Lit., 230-235 ^°^C) [21], FT-IR (KBr) ῡ(cm^-1^): 3299, 3029, 2920, 1635, 1596, 1557, 1445, 1312, 1293, 1229, 911, 776, 735, 695, 498 cm^-1^. ^1^H NMR (400 MHz, DMSO-*d_6_*) δ (ppm): 2.28 (s, 3H), 6.78-6.80 (m, 1H), 6.95-6.99 (m, 1H), 7.14-7.18 (m, 1H), 7.23-7.32 (m, 4H), 7.45-7.48 (m, 2H), 8.59 (s, 1H), 8.65 (s, 1H). ^13^C NMR (100 MHz, DMSO-*d_6_*) δ (ppm): 21.7, 115.8, 118.6, 119.1, 122.2, 123.0, 129.0, 129.2, 138.4, 140.0, 140.2, 152.9. Anal Calcd for: C_14_H_14_N_2_O: C, 74.31; H, 6.24; N, 12.38%. Found: C, 75.62; H, 5.93; N, 12.09%.

**1-Methyl-1,3-diphenyl urea (C26**)

White solid, yield: 85%, mp: 99-101 ^°^C (Lit., 93-94 ^°^C) [22], FT-IR (KBr) ῡ(cm^-1^): 3351, 3040, 1645, 1596, 1526, 1501, 1446, 1303, 1241, 1157, 1123, 1027, 906, 870, 792, 749, 693 cm^-1^. ^1^H NMR (400 MHz, DMSO-*d_6_*) δ (ppm): 3.29 (s, 3H), 6.93-6.98 (m, 1H), 7.21-7.28 (m, 3H), 7.32-7.35 (m, 2H), 7.40-7.46 (m, 4H), 8.15 (s, 1H). ^13^C NMR (100 MHz, DMSO-*d_6_*) δ (ppm): 38.0, 120.3, 122.4, 126.1, 126.6, 128.7, 129.6, 140.5, 144.5, 155.1. Anal Calcd for: C_14_H_14_N_2_O: C, 74.31; H, 6.24; N, 12.38%. Found: C, 75.11; H, 6.36; N, 11.96%.

**1-Methyl-3-phenyl urea (C27)**

White solid, yield: 97%, mp: 152 ^°^C (Lit., 146-147 ^°^C) [23], FT-IR (KBr) ῡ(cm^-1^): 3360, 3314, 3042, 2946, 2805, 1646, 1592, 1574, 1500, 1442, 1417, 1169, 1078, 903, 758, 735, 697, 508 cm^-1^. ^1^H NMR (400 MHz, DMSO-*d_6_*) δ (ppm): 2.64-2.65 (d, J= 4.4 Hz, 2H), 5.98-6.01 (q, J= 4.4 Hz, 1H), 6.86-6.90 (m, 1H), 7.19-7.24 (m, 2H), 7.38-7.41 (m, 2H), 8.48 (s, 1H). ^13^C NMR (100 MHz, DMSO-*d_6_*) δ (ppm): 26.6, 118.0, 121.3, 129.0, 141.0, 156.3. Anal Calcd for: C_8_H_10_N_2_O: C, 63.98; H, 6.71; N, 18.65%. Found: C, 64.70; H, 6.86; N, 17.96%.

**1-Ethyl-3-phenyl urea (C28**)

White solid, yield: 95%, mp: 99-101 ^°^C (Lit., 100-101 ^°^C) [19], FT-IR (KBr) ῡ(cm^-1^): 3327, 3186, 3034, 2975, 2880, 1648, 1604, 1571, 1500, 1486, 1446, 1311, 1243, 1178, 1077, 929, 898, 840, 741, 693, 507 cm^-1^. ^1^H NMR (400 MHz, DMSO-*d_6_*) δ (ppm): 1.03-1.07 (t, J= 7.2 Hz, 3H), 3.07-3.14 (q, J= 6.4 Hz, 2H), 6.07-6.10 (t, J= 5.2 Hz, 1H), 6.86-6.90 (m, 1H), 7.19-7.23 (m, 2H), 7.38-7.40 (m, 2H), 8.39 (s, 1H). ^13^C NMR (100 MHz, DMSO-*d_6_*) δ (ppm): 15.9, 34.3, 118.0, 121.3, 129.0, 141.0, 155.5. Anal Calcd for: C_9_H_12_N_2_O: C, 65.83; H, 7.37; N, 17.06%. Found: C, 64.70; H, 7.86; N, 16.68%.

**1-Butyl-3-phenyl urea** (**C29**)

White solid, yield: 92%, mp: 130 ^°^C (Lit., 129-131 ^°^C)[24], FT-IR (KBr) ῡ(cm^-1^): 3351, 3039, 2925, 1645, 1596, 1526, 1446, 1353, 1303, 1241, 1157, 1123, 1027, 749, 693 cm^-1^. ^1^H NMR (400 MHz, DMSO-*d_6_*) δ (ppm): 0.88-0.91 (t, J= 7.2 Hz, 3H), 1.26-1.36 (dt, 2H), 1.38-1.45 (p, 2H), 3.05-3.11 (J= 6.4 Hz, q, 3H), 6.01 (s, 1H), 6.85-6.89 (m, 1H), 7.19-7.23 (m, 2H), 7.37-7.39 (m, 2H), 8.37 (s, 1H). ^13^C NMR (100 MHz, DMSO-*d_6_*) δ (ppm): 14.1, 20.0, 32.3, 39.1, 117.9, 121.3, 129.0, 141.0, 155.6. Anal Calcd for: C_34_H_16_N_2_O: C, 87.16; H, 3.44; N, 5.98%. Found: C, 86.09; H, 4.09; N, 5.11%.

**1-Benzyl-3-phenyl urea** (**C30**)

White solid, yield: 90%, mp: 171-172 ^°^C (Lit., 168-171 ^°^C) [25], FT-IR (KBr) ῡ(cm^-1^): 3330, 2876, 1954, 1625, 1552, 1310, 1233, 1080, 1050, 898, 848, 698, 499 cm^-1^. ^1^H NMR (400 MHz, DMSO-*d_6_*) δ (ppm): 4.30-4.31 (d, J= 6.0 Hz, 2H), 6.59-6.62 (t, J= 6.4 Hz, 1H), 6.88-6.92 (m, 1H), 7.20-7.27 (m, 3H), 7.29-7.36 (m, 4H), 7.39-7.42 (m, 2H), 8.56 (s, 1H). ^13^C NMR (100 MHz, DMSO-*d_6_*) δ (ppm): 43.1, 118.1, 121.5, 127.1, 127.5, 128.7, 129.1, 140.8, 140.9, 155.6. Anal Calcd for: C_37_H_14_N_2_O: C, 88.43; H, 2.81; N, 5.57%. Found: C, 89.71; H, 2.21; N, 5.01%.

***N*-Phenylmorpholine-4-carboxamide** (**C31**)

 White solid, yield: 83%, mp: 163 ^°^C (Lit., 156-158 ^°^C) [26], FT-IR (KBr) ῡ(cm^-1^): 3436, 3269, 2953, 2858, 2347, 1634, 1573, 1600, 1415, 1303, 1114, 992, 873, 746, 691, 578, 503 cm^-1^. ^1^H NMR (400 MHz, DMSO-*d_6_*) δ (ppm): 3.38-3.44 (t, 4H), 3.59-3.62 (t, 4H), 8.15 (s, 1H), 6.92-6.96 (m, 1H), 7.21-7.26 (m, 2H), 7.45-7.48 (m, 2H), 8.54 (s, 1H). ^13^C NMR (100 MHz, DMSO-*d_6_*) δ (ppm): 44.1, 65.9, 119.5, 121.7, 128.2, 140.3, 155.1. Anal Calcd for: C_11_H_14_N_2_O_2_: C, 64.06; H, 6.84; N, 13.58%. Found: C, 65.68; H, 6.38; N, 13.21%.


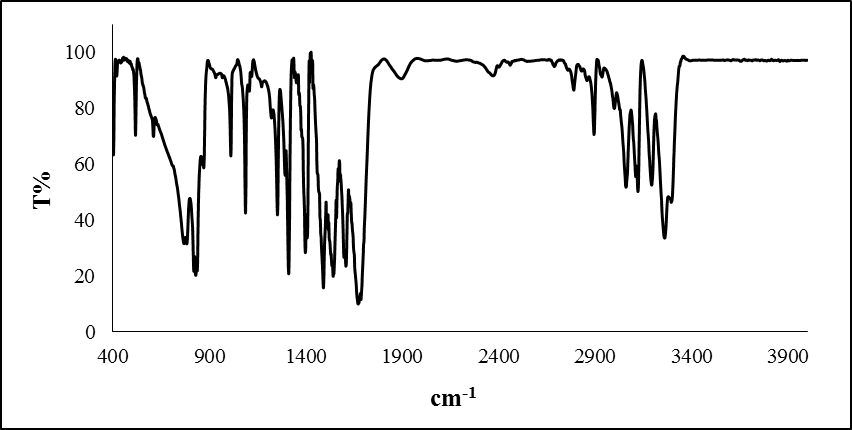


**Figure S1:** FT-IR spectrum of *N*-(4-chlorophenyl) formamide in KBr


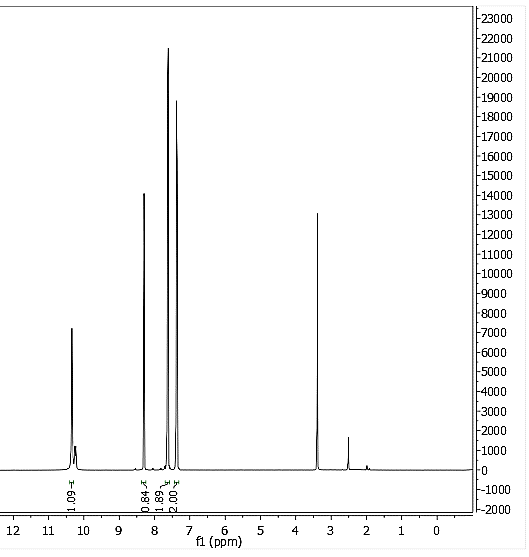


**Figure S2:** ^1^H-NMR spectrum (400MHz) of *N*-(4-chlorophenyl) formamide in DMSO-*d_6_*


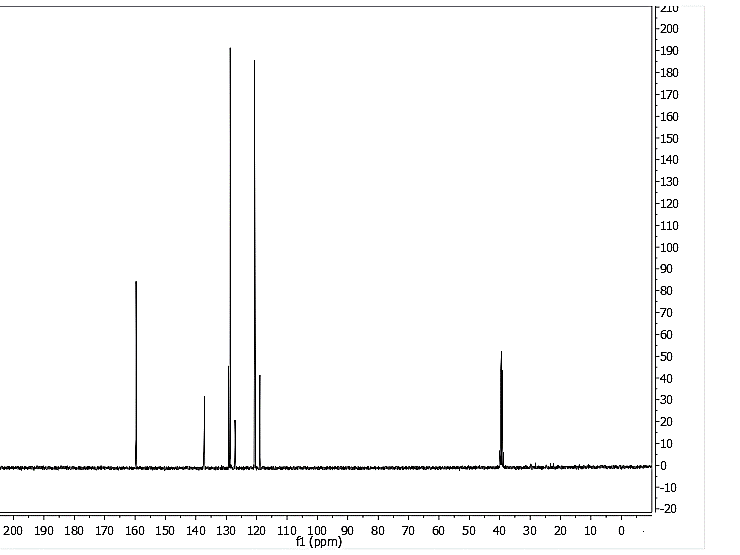


**Figure S3:** ^13^C-NMR spectrum (100 MHz) of *N*-(4-chlorophenyl) formamide in DMSO-*d_6_*

**
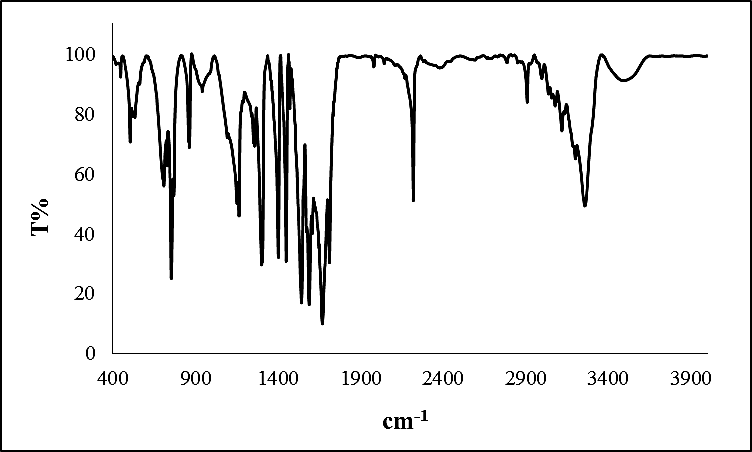
**

**Figure S4:** FT-IR spectrum of *N*-(2-cyanophenyl) formamide in KBr


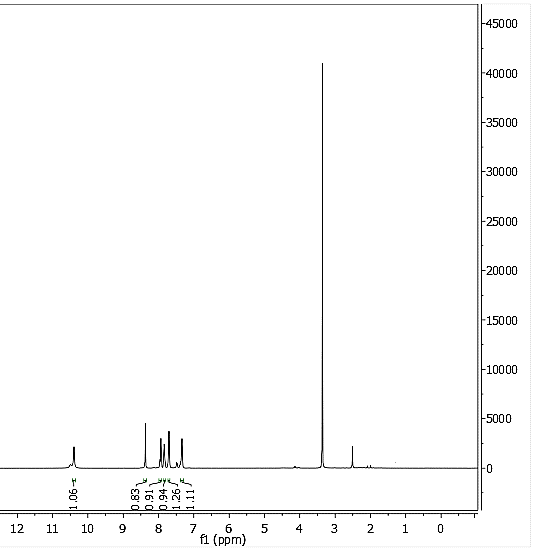


**Figure S5:** ^1^H-NMR spectrum (400MHz) of *N*-(2-cyanophenyl) formamide in DMSO-*d_6_*


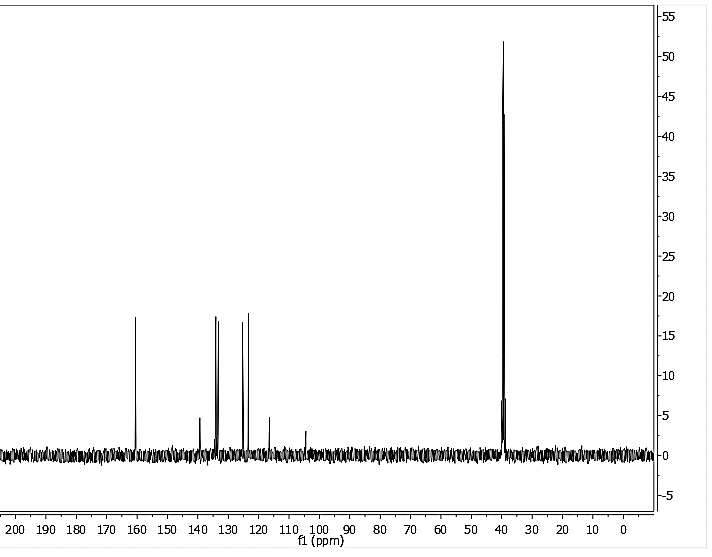


**Figure S6:** ^13^C-NMR spectrum (100 MHz) of *N*-(2-cyanophenyl) formamide in DMSO-*d_6_*


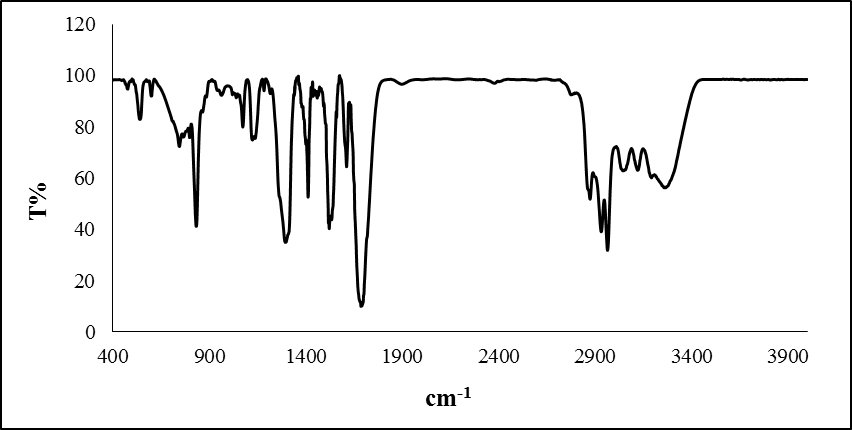


**Figure S7:** FT-IR spectrum of *N*-(4-ethylphenyl) formamide in KBr


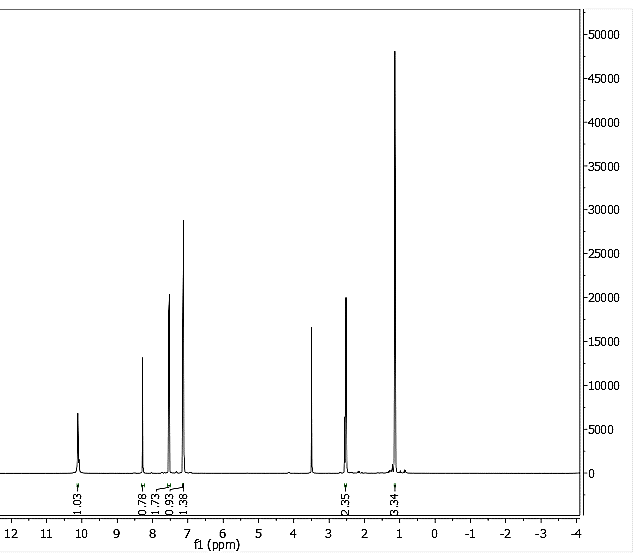


**Figure S8:** ^1^H-NMR spectrum (400MHz) of *N*-(4-ethylphenyl) formamide in DMSO-*d_6_*


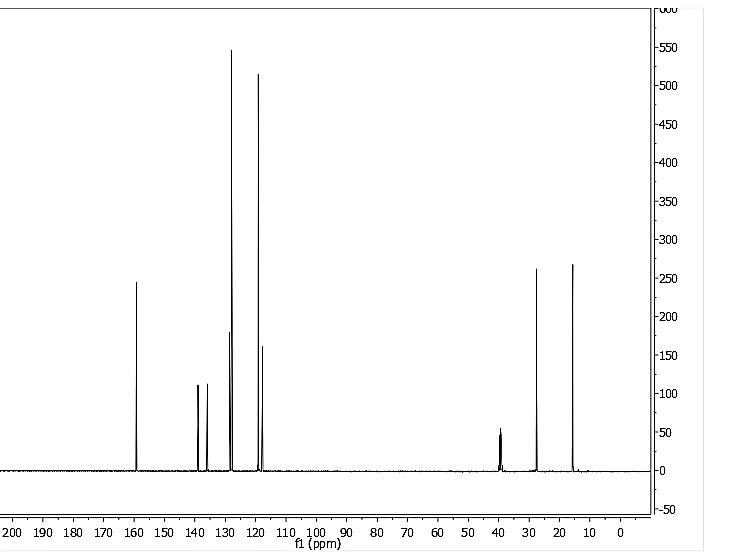


**Figure S9:** ^13^C-NMR spectrum (100 MHz) of *N*-(4-ethylphenyl) formamide in DMSO-*d_6_*


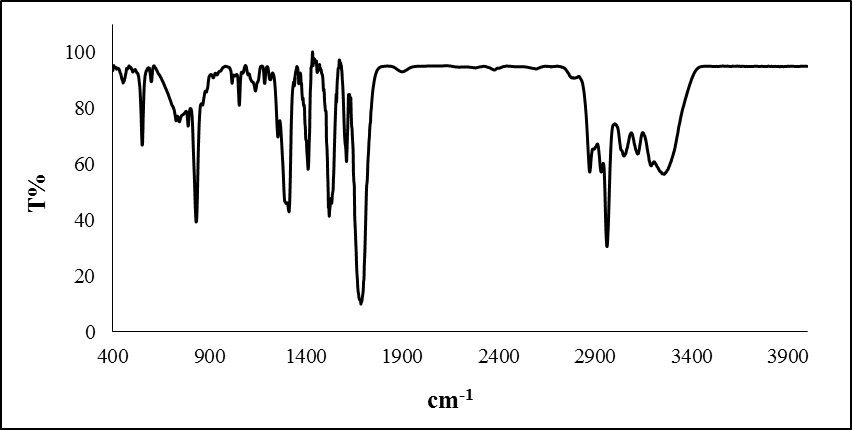


**Figure S10:** FT-IR spectrum of *N*-(4-isopropylphenyl) formamide in KBr


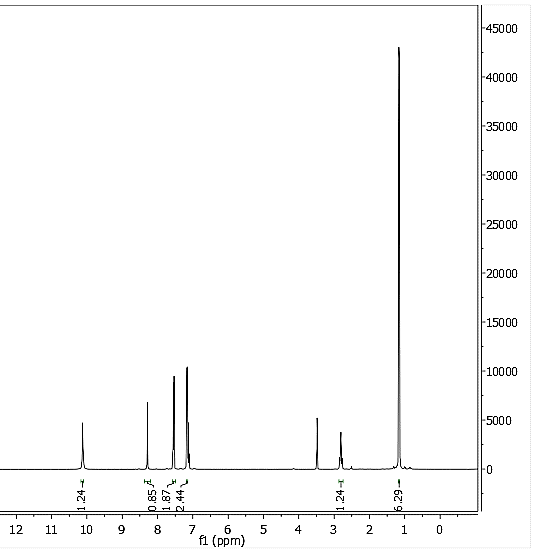


**Figure S11:** ^1^H-NMR spectrum (400MHz) of *N*-(4-isopropylphenyl) formamide in DMSO-*d_6_*


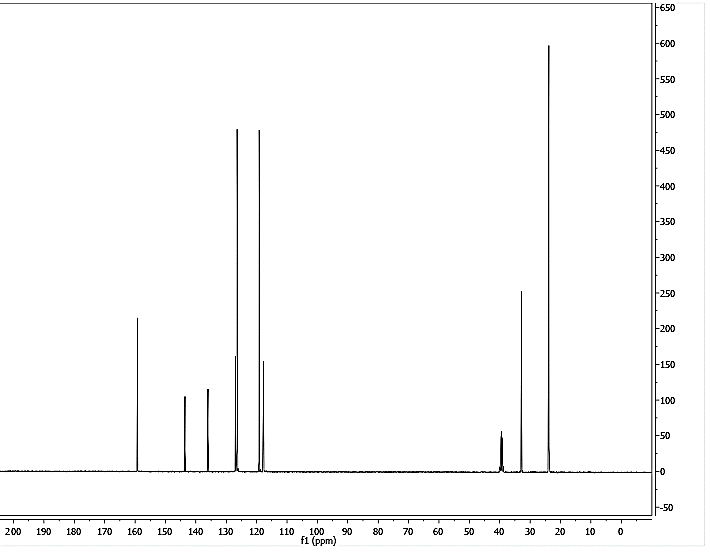


**Figure S12:** ^13^C-NMR spectrum (100 MHz) of *N*-(4-isopropylphenyl) formamide in DMSO-*d_6_*


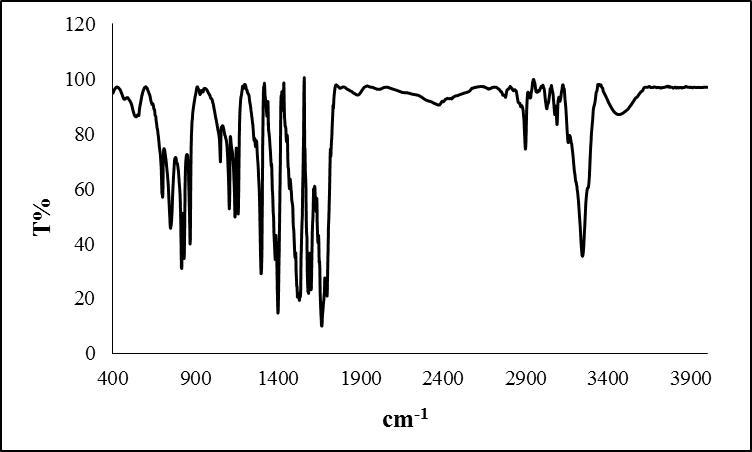


**Figure S13:** FT-IR spectrum of *N*-(2,4-dichlorophenyl)formamide in KBr


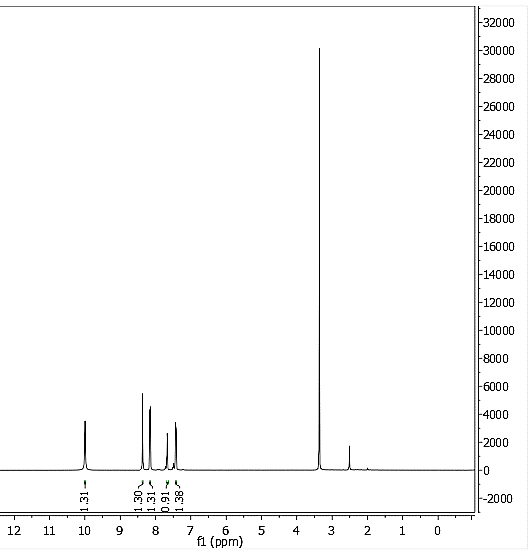


**Figure S14:** ^1^H-NMR spectrum (400MHz) of *N*-(2,4-dichlorophenyl) formamide in DMSO-*d_6_*


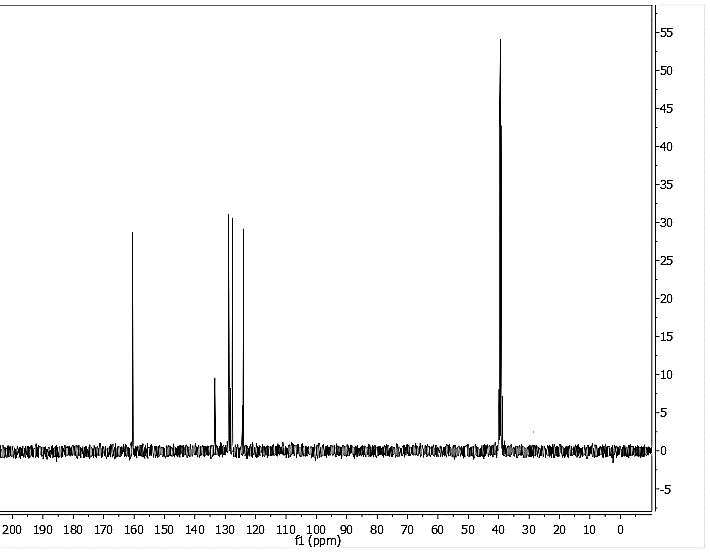


**Figure S15:** ^13^C-NMR spectrum (100 MHz) of *N*-(2,4-dichlorophenyl) formamide in DMSO-*d_6_*


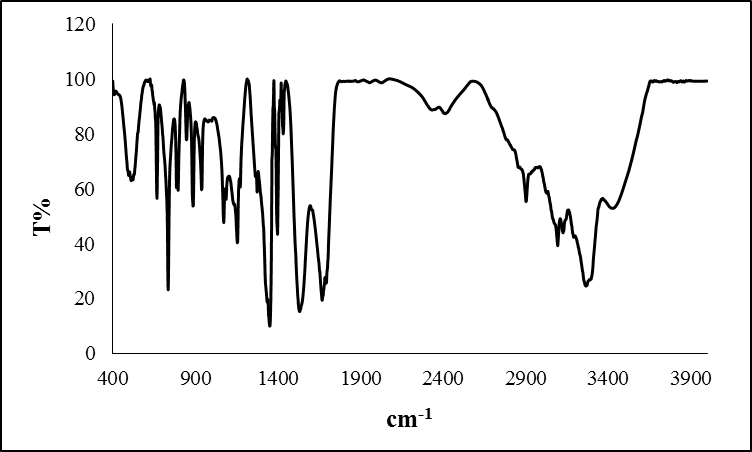


**Figure S16:** FT-IR spectrum of *N*-(3-nitrophenyl) formamide in KBr


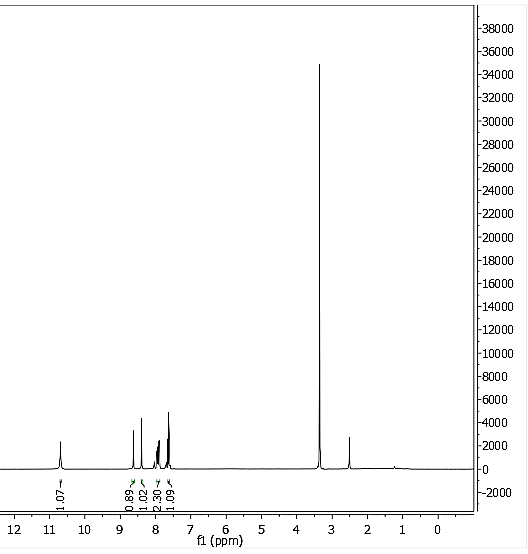


**Figure S17:** ^1^H-NMR spectrum (400MHz) of *N*-(3-nitrophenyl) formamide in DMSO-*d_6_*

**
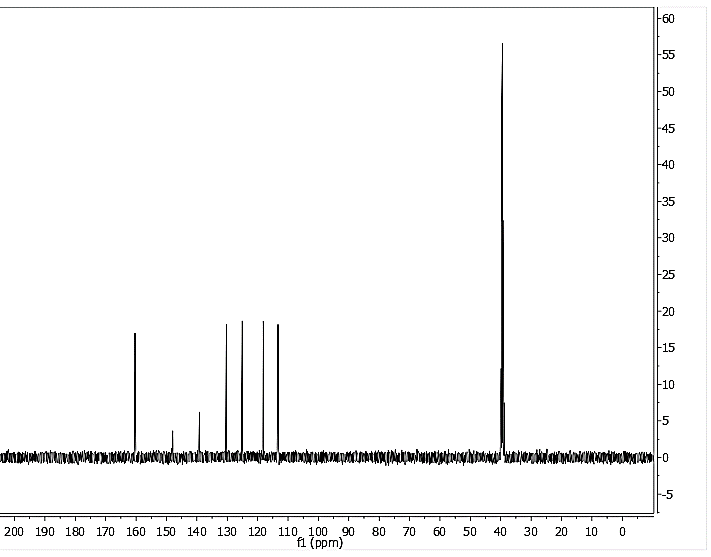
**

**Figure S18:** ^13^C-NMR spectrum (100 MHz) of *N*-(3-nitrophenyl) formamide in DMSO-*d_6_*


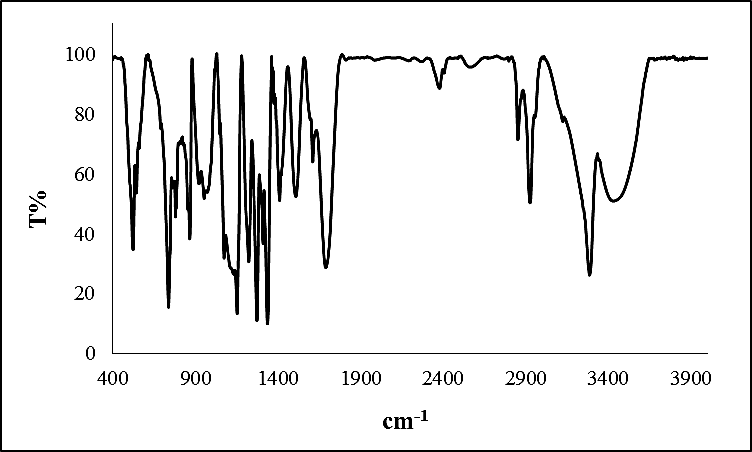


**Figure S19:** FT-IR spectrum of *N*-(2-nitrophenyl) formamide in KBr


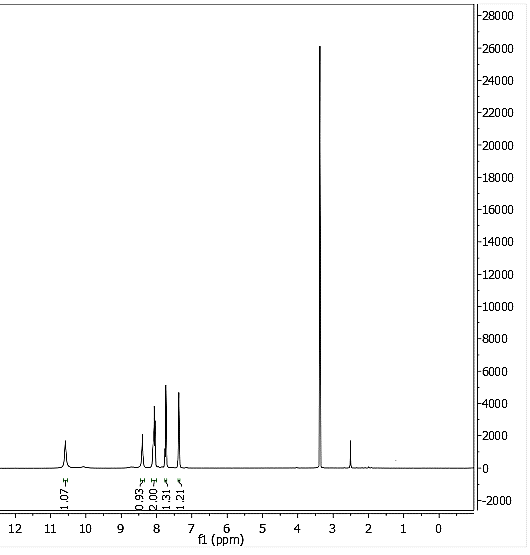


**Figure S20:** ^1^H-NMR spectrum (400MHz) of *N*-(2-nitrophenyl) formamide in DMSO-*d_6_*


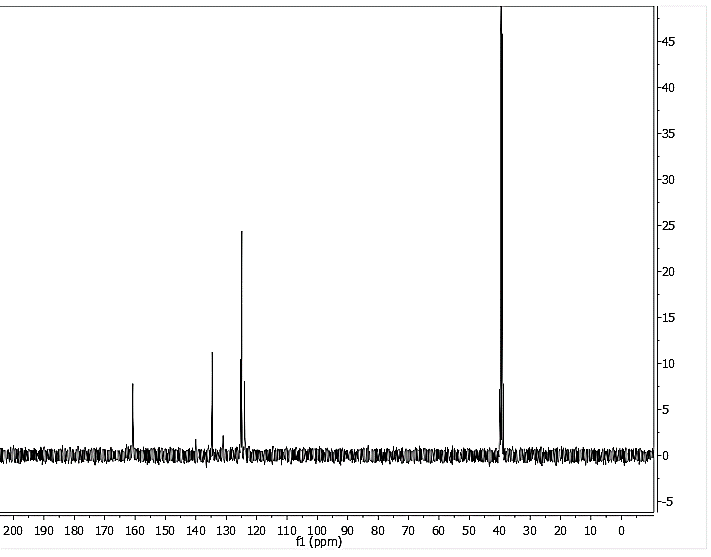


**Figure S21:** ^13^C-NMR spectrum (100 MHz) of *N*-(2-nitrophenyl) formamide in DMSO-*d_6_*


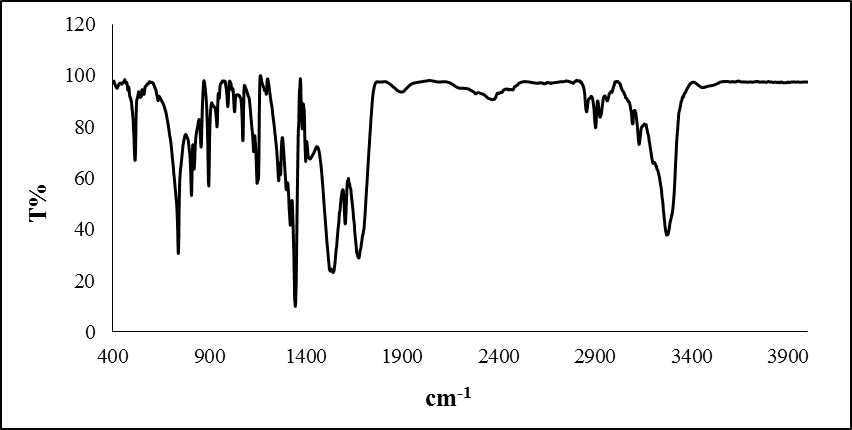


**Figure S22:** FT-IR spectrum of *N*-(2-methyl-5-nitrophenyl) formamide in KBr


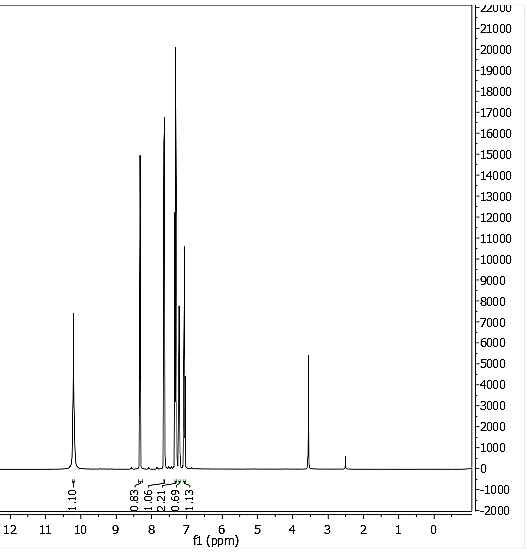


**Figure S23:** ^1^H-NMR spectrum (400MHz) of *N*-(2-methyl-5-nitrophenyl) formamide in DMSO-*d_6_*


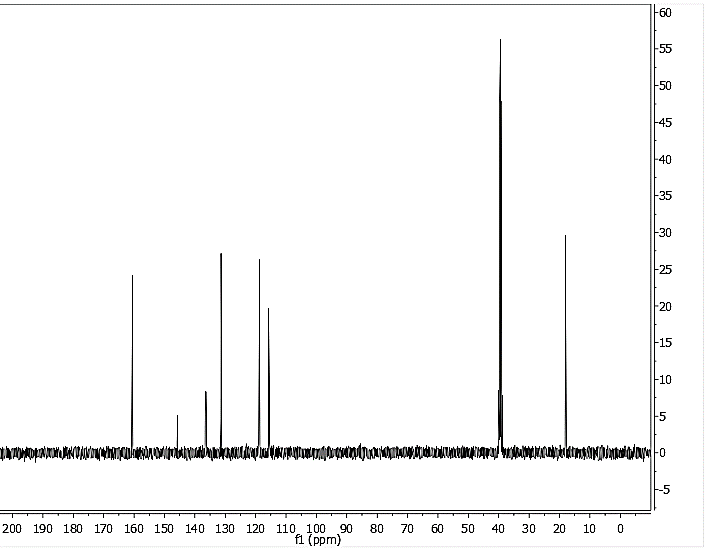


**Figure S24:** ^13^C-NMR spectrum (100 MHz) of *N*-(2-methyl-5-nitrophenyl) formamide in DMSO-*d_6_*


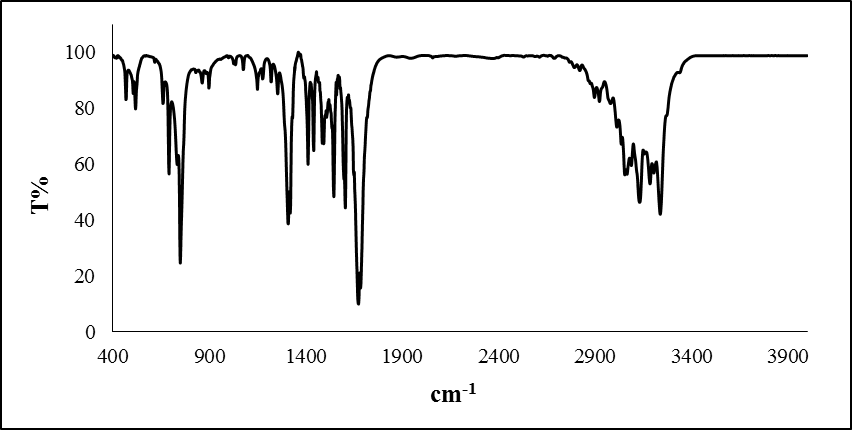


**Figure S25:** FT-IR spectrum of *N*-phenyl formamide in KBr


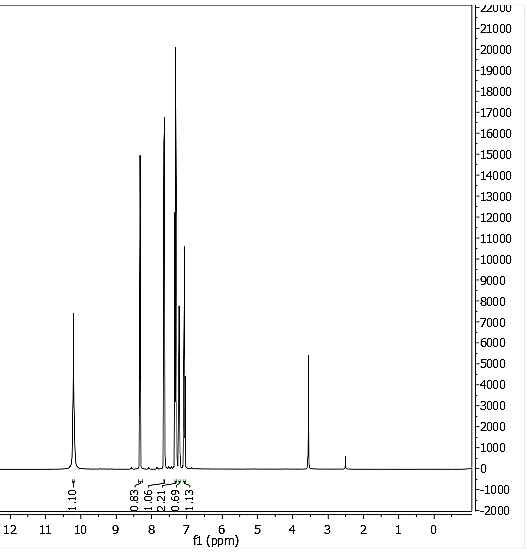


**Figure S26:** ^1^H-NMR spectrum (400MHz) of *N*-phenyl formamide in DMSO-*d_6_*


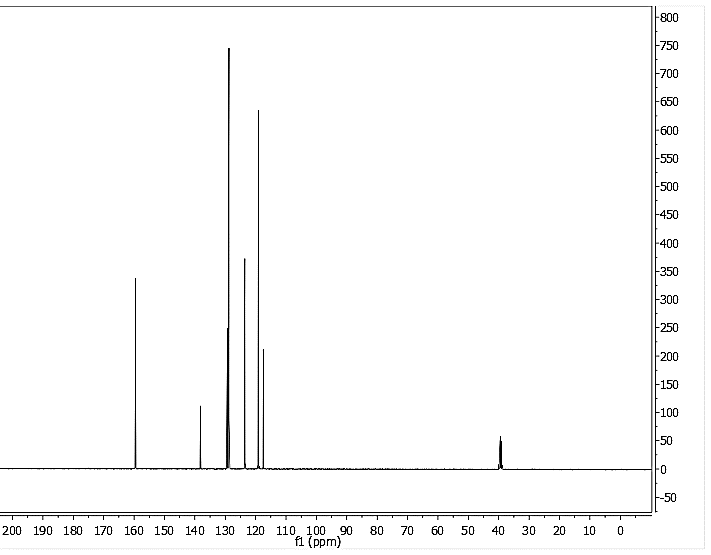


**Figure S27:** ^13^C-NMR spectrum (100 MHz) of *N*-phenyl formamide in DMSO-*d_6_*


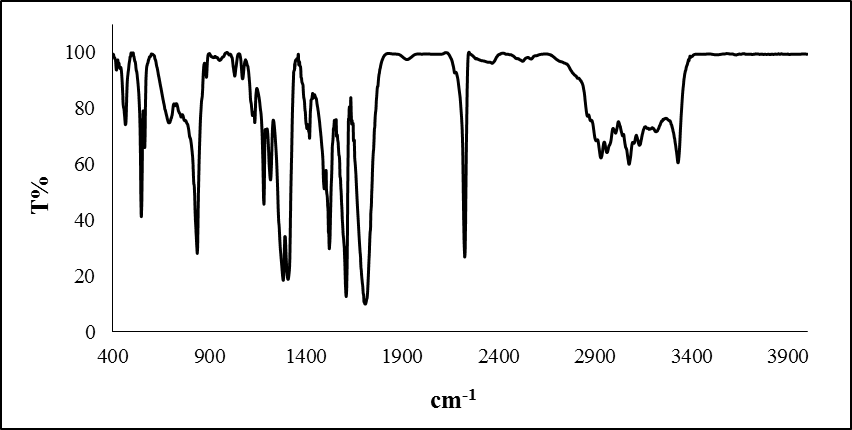


**Figure S28:** FT-IR spectrum of *N*-(4-cyanophenyl) formamide in KBr


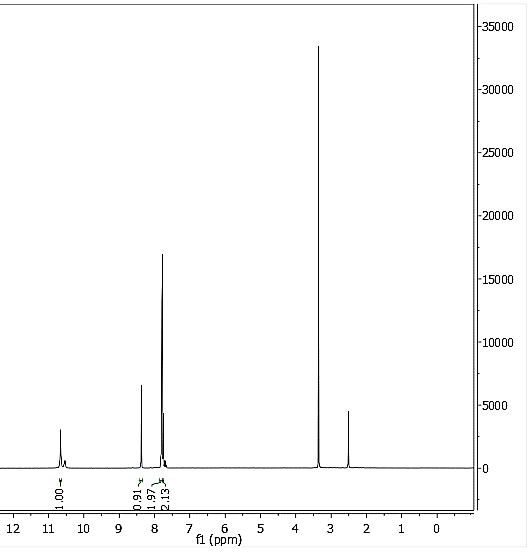


**Figure S29:** ^1^H-NMR spectrum (400MHz) of *N*-(4-cyanophenyl) formamide in DMSO-*d_6_*


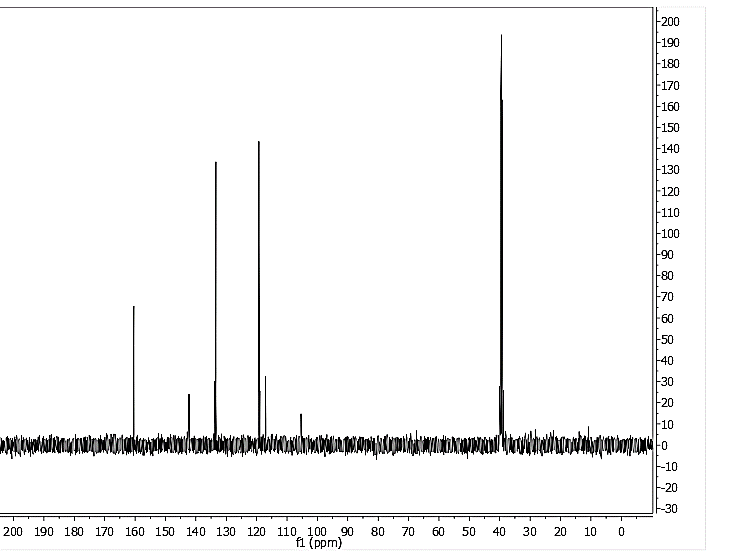


**Figure S30:** ^13^C-NMR spectrum (100 MHz) of *N*-(4-cyanophenyl) formamide in DMSO-*d_6_*


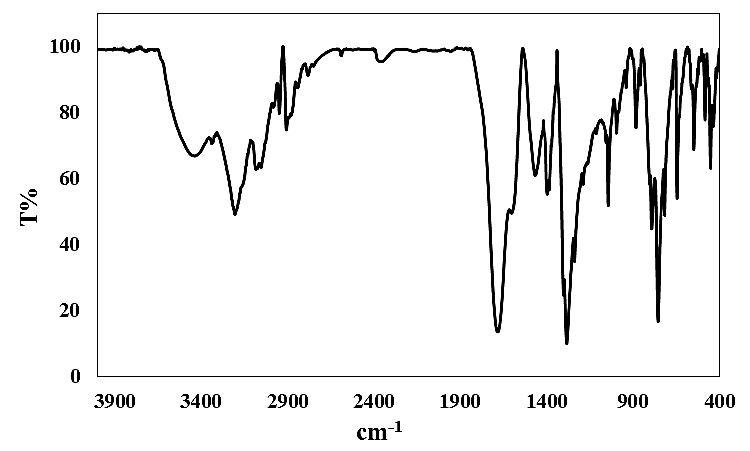


**Figure S31:** FT-IR spectrum of *N*-*o*-tolyl formamide in KBr


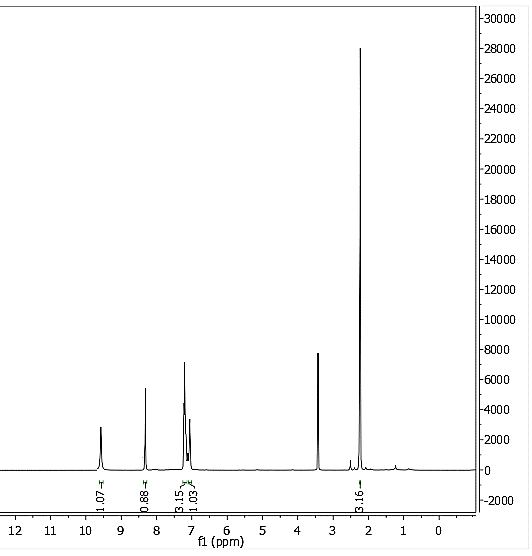


**Figure S32:** ^1^H-NMR spectrum (400MHz) of *N*-*o*-tolyl formamide in DMSO-*d_6_*


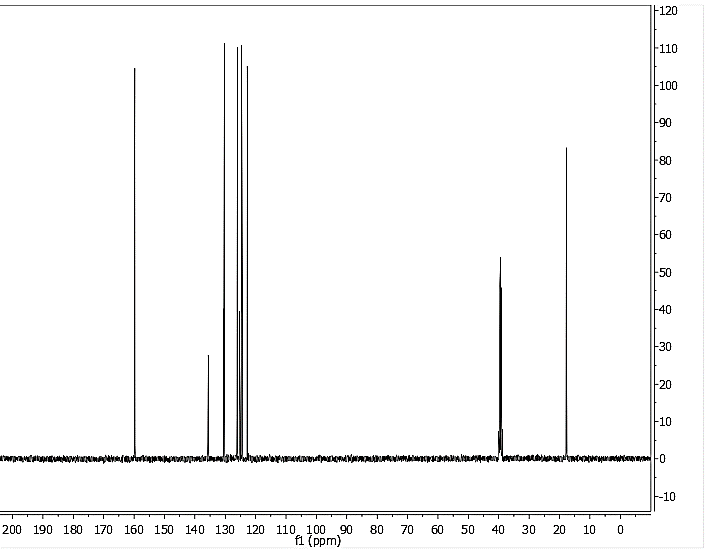


**Figure S33:** ^13^C-NMR spectrum (100 MHz) of *N*-*o*-tolyl formamide in DMSO-*d_6_*

**
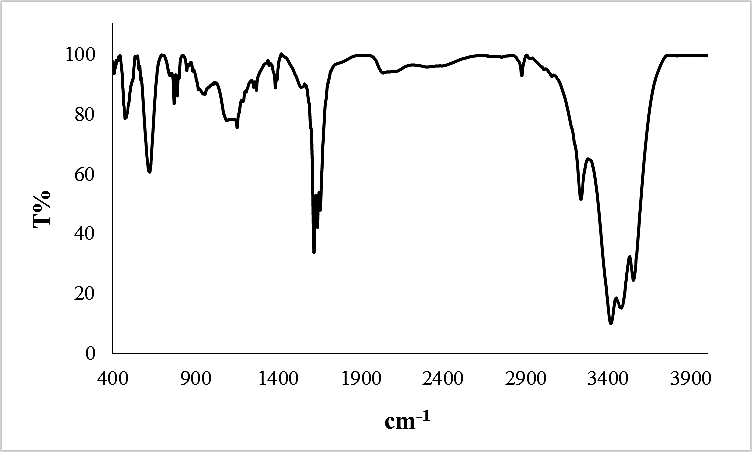
**

**Figure S34:** FT-IR spectrum of *N*-(naphthalen-1-yl) formamide in KBr


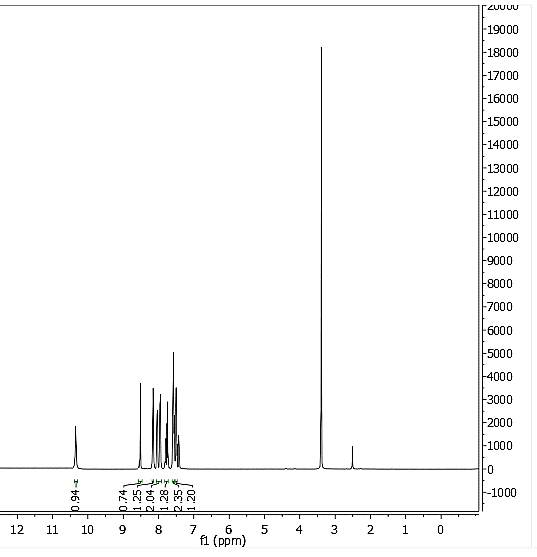


**Figure S35:** ^1^H-NMR spectrum (400MHz) of *N*-(naphthalen-1-yl) formamide in DMSO-*d_6_*


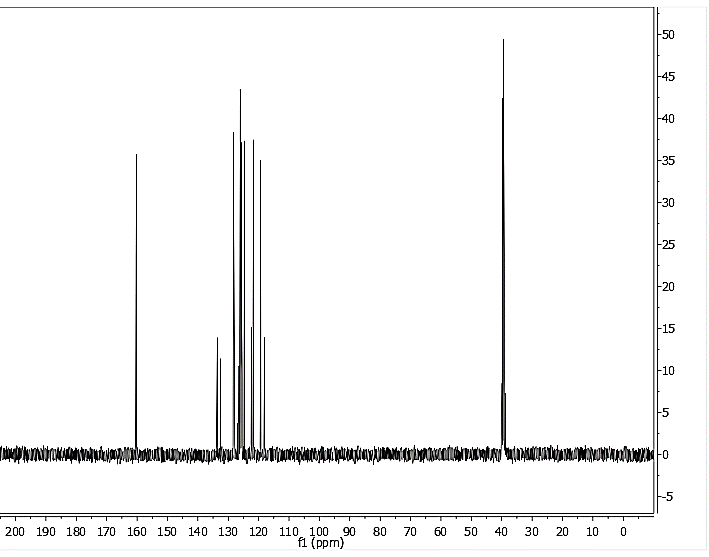


**Figure S36:** ^13^C-NMR spectrum (100 MHz) of *N*-(naphthalen-1-yl) formamide in DMSO-*d_6_*


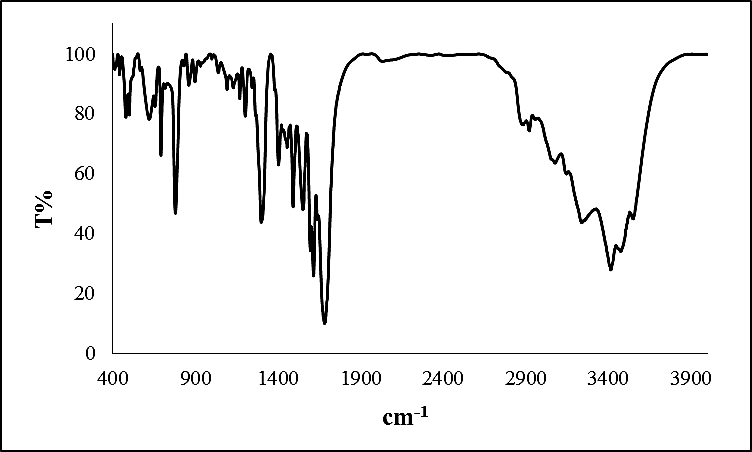


**Figure S37:** FT-IR spectrum of *N*-*m*-tolyl formamide in KBr


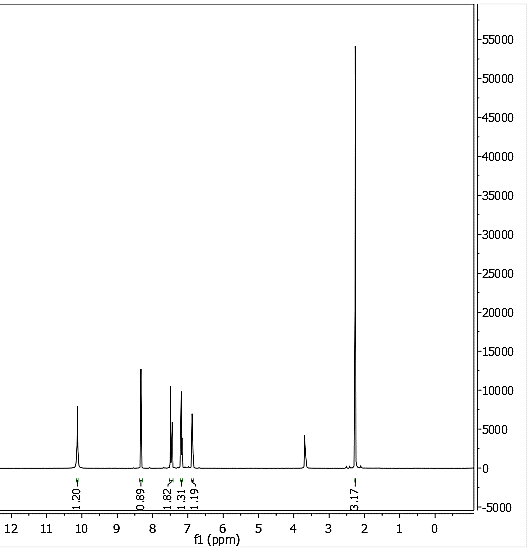


**Figure S38:** ^1^H-NMR spectrum (400MHz) of *N*-*m*-tolyl formamide in DMSO-*d_6_*

**
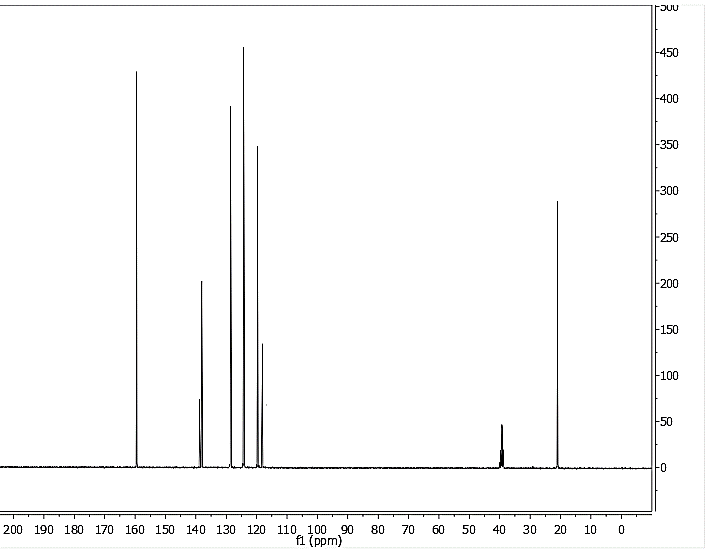
**

**Figure S39:** ^13^C-NMR spectrum (100 MHz) of *N*-*m*-tolyl formamide in DMSO-*d_6_*


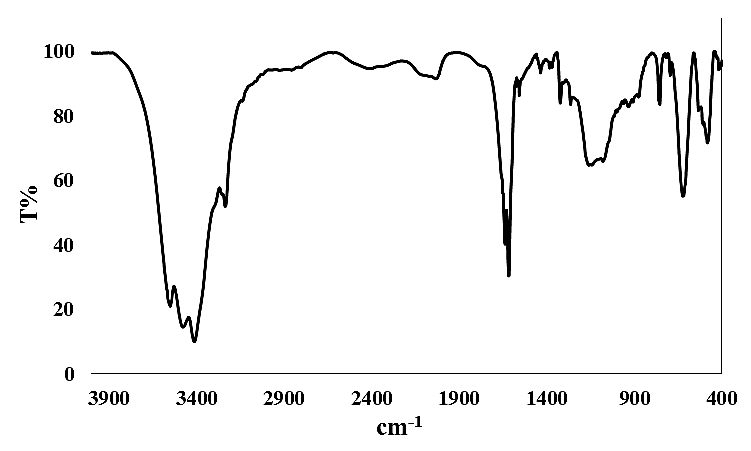


**Figure S40:** FT-IR spectrum of *N*-phenyl acetamide in KBr


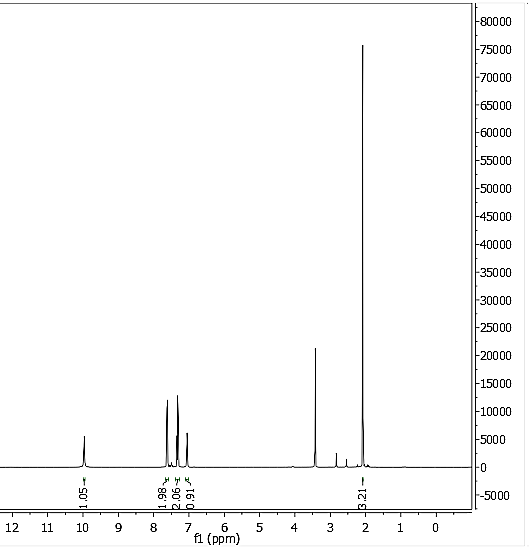


**Figure S41:** ^1^H-NMR spectrum (400MHz) of *N*-phenyl acetamide in DMSO-*d_6_*


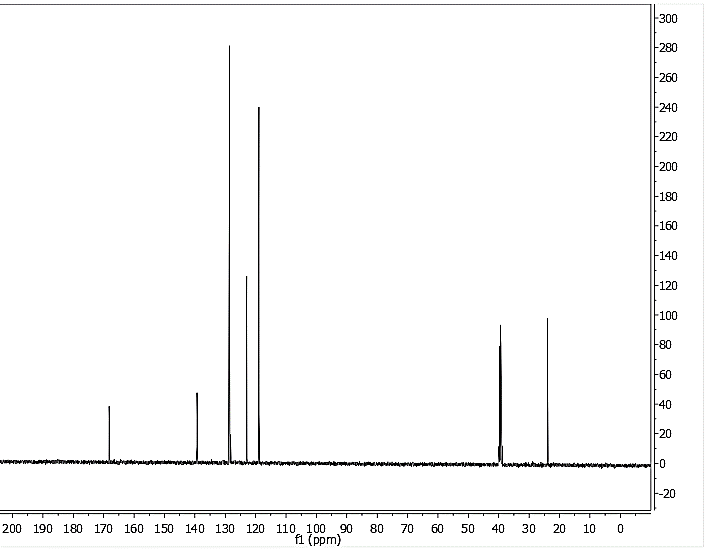


**Figure S42:** ^13^C-NMR spectrum (100 MHz) of *N*-phenyl acetamide in DMSO-*d_6_*


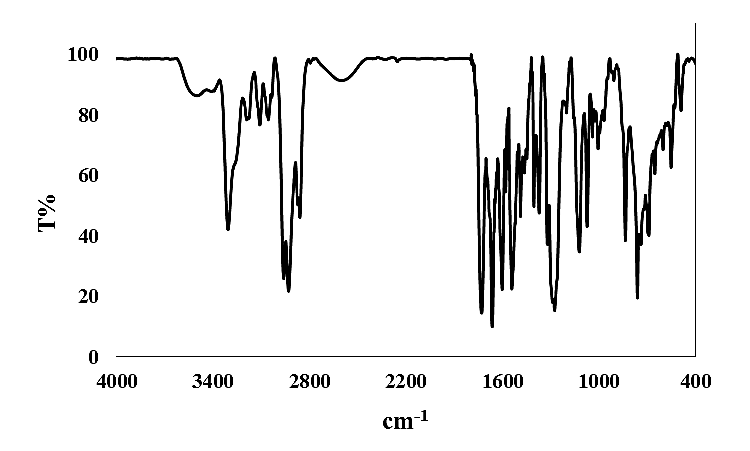


**Figure S43:** FT-IR spectrum of *N*-([1,1'-biphenyl]-4-yl) acetamide in KBr


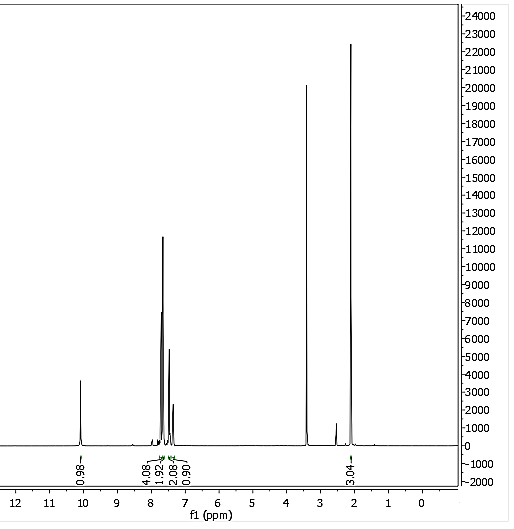


**Figure S44:** ^1^H-NMR spectrum (400MHz) of *N*-([1,1'-biphenyl]-4-yl) acetamide in DMSO-*d_6_*


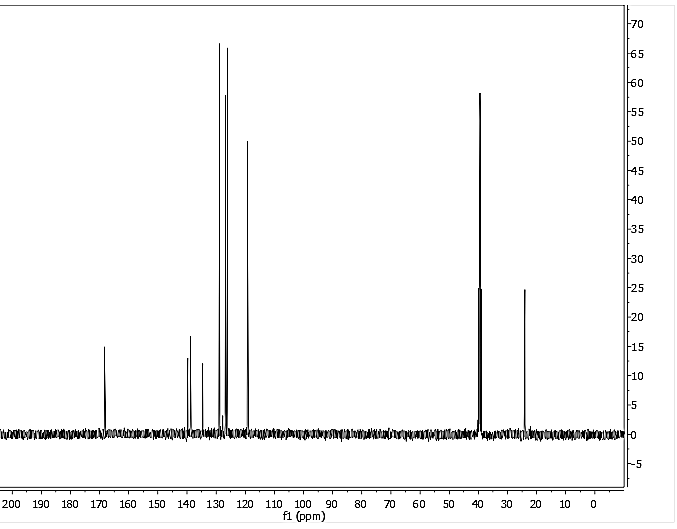


**Figure S45:** ^13^C-NMR spectrum (100 MHz) of *N*-([1,1'-biphenyl]-4-yl) acetamide in DMSO-*d_6_*


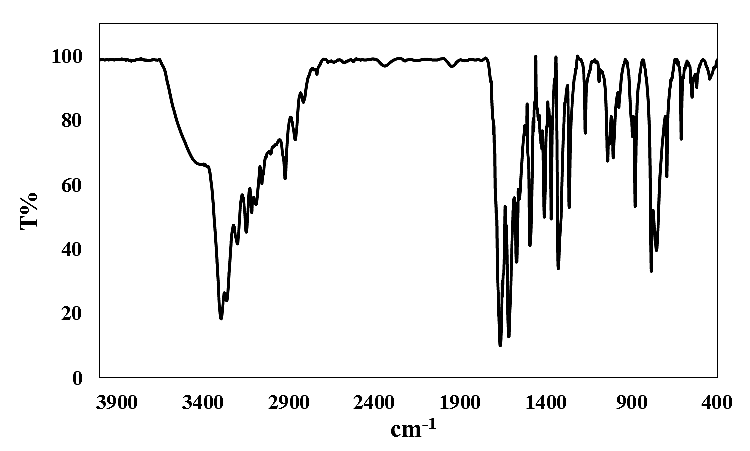


**Figure S46:** FT-IR spectrum of *N*-(*m*-tolyl) acetamide in KBr


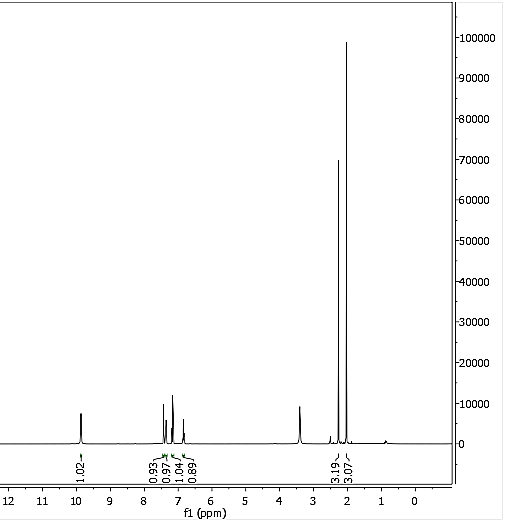


**Figure S47:** ^1^H-NMR spectrum (400MHz) of *N*-(*m*-tolyl) acetamide in DMSO-*d_6_*


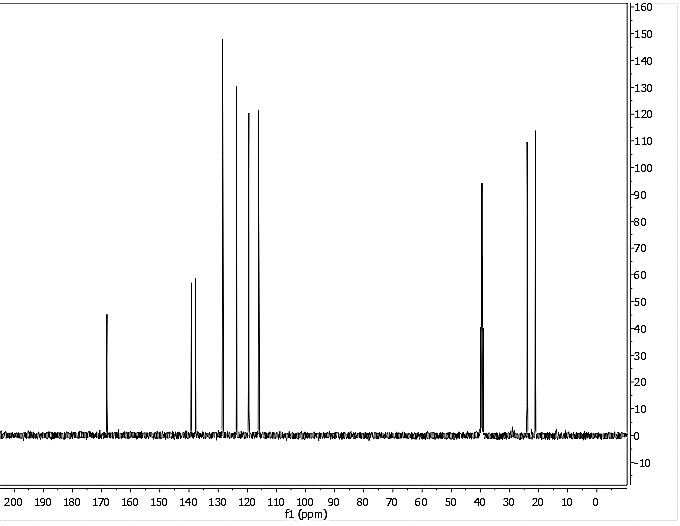


**Figure S48:** ^13^C-NMR spectrum (100 MHz) of *N*-(*m*-tolyl) acetamide in DMSO-*d_6_*


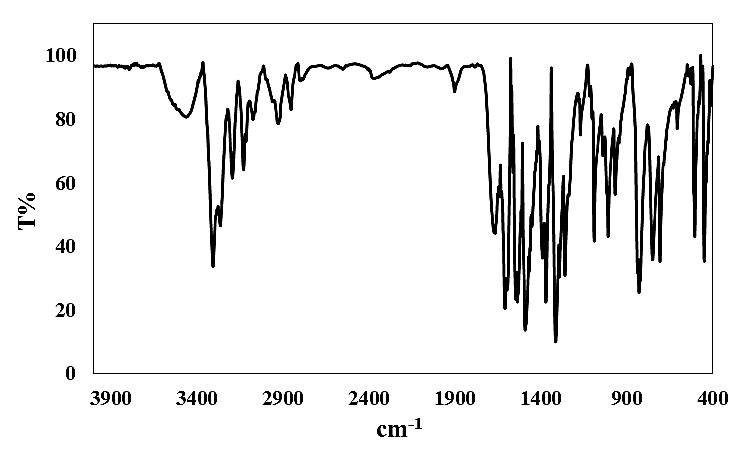


**Figure S49:** FT-IR spectrum of *N*-(4-chlorophenyl) acetamide in KBr

**
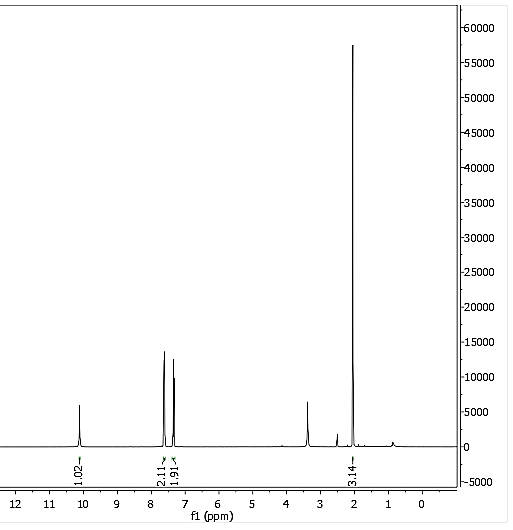
**

**Figure S50:** ^1^H-NMR spectrum (400MHz) of *N*-(4-chlorophenyl) acetamide in DMSO-*d_6_*


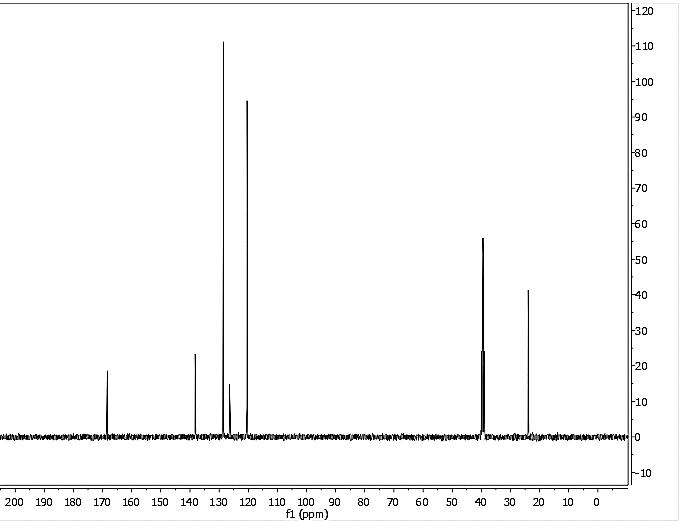


**Figure S51:** ^13^C-NMR spectrum (100 MHz) of *N*-(4-chlorophenyl) acetamide in DMSO-*d_6_*


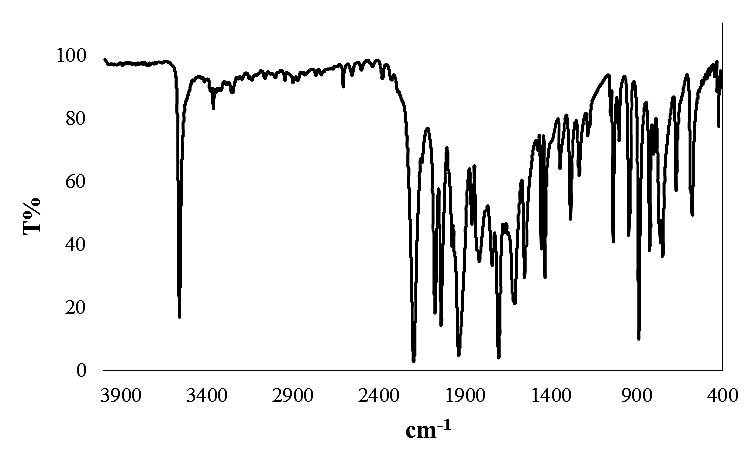


**Figure S52:** FT-IR spectrum of *N*-(2-nitrophenyl) acetamide in KBr


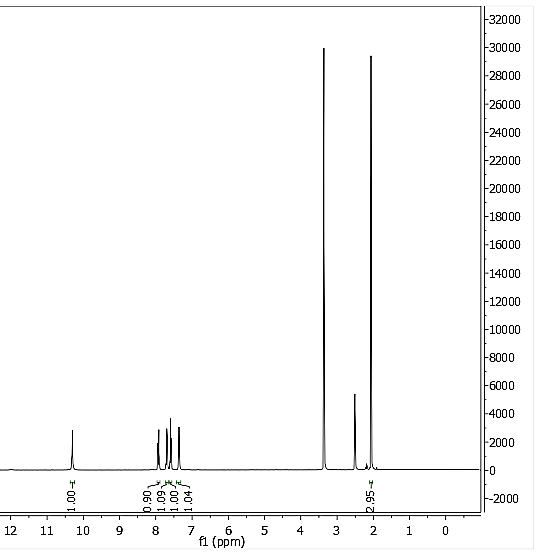


**Figure S53:** ^1^H-NMR spectrum (400MHz) of *N*-(2-nitrophenyl) acetamide in DMSO-*d_6_*


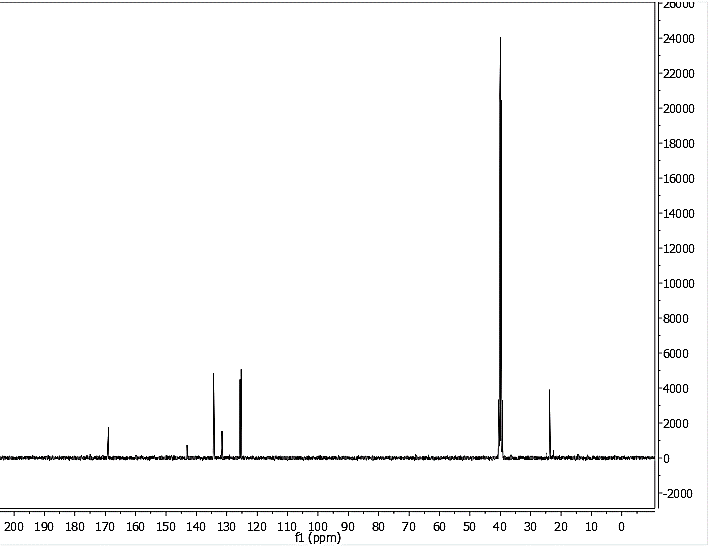


**Figure S54:** ^13^C-NMR spectrum (100 MHz) of *N*-(2-nitrophenyl) acetamide in DMSO-*d_6_*


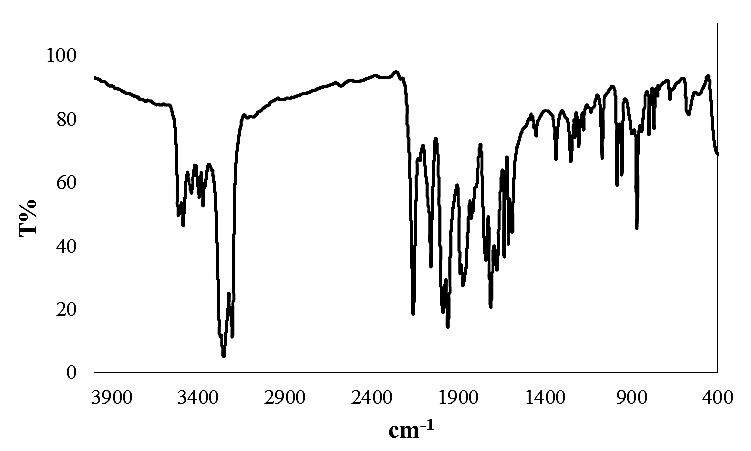


**Figure S55:** FT-IR spectrum of *N*-(3-nitrophenyl) acetamide in KBr


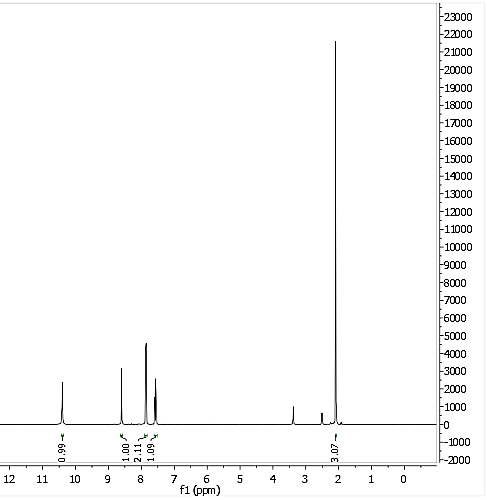


**Figure S56:** ^1^H-NMR spectrum (400MHz) of *N*-(3-nitrophenyl) acetamide in DMSO-*d_6_*


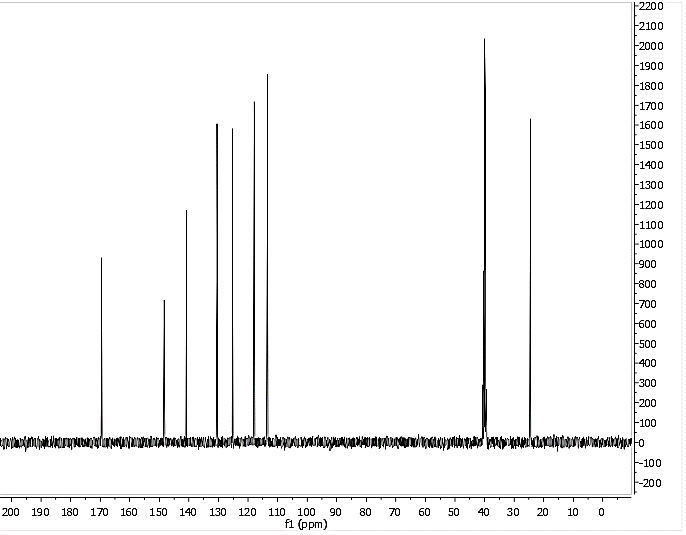


**Figure S57:** ^13^C-NMR spectrum (100 MHz) of *N*-(3-nitrophenyl) acetamide in DMSO-*d_6_*

**
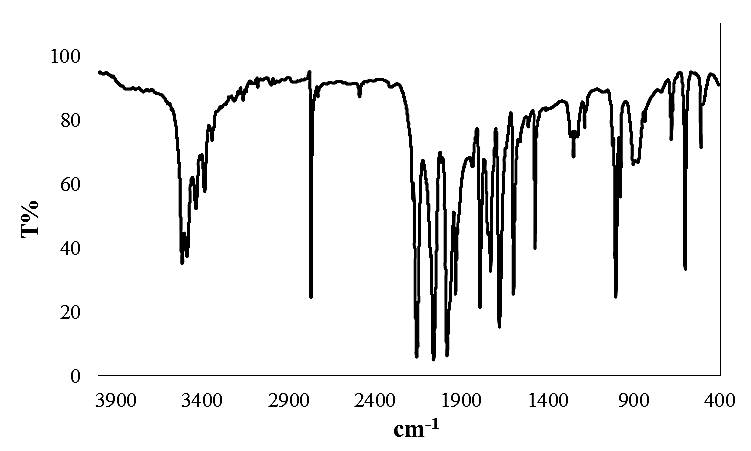
**

**Figure S58:** FT-IR spectrum of *N*-(4-cyanophenyl) acetamide in KBr


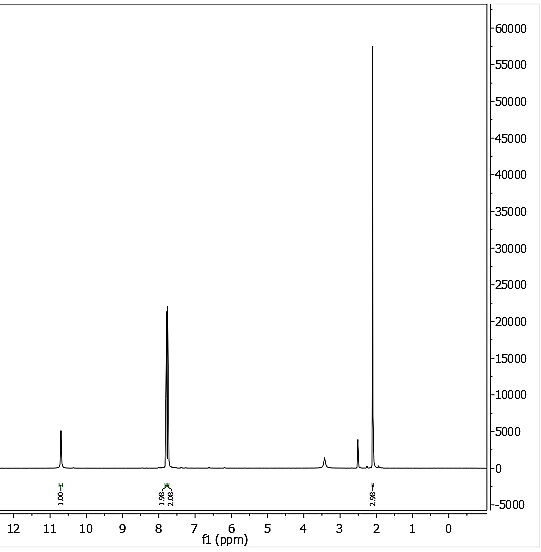


**Figure S59:** ^1^H-NMR spectrum (400MHz) of *N*-(4-cyanophenyl) acetamide in DMSO-*d_6_*


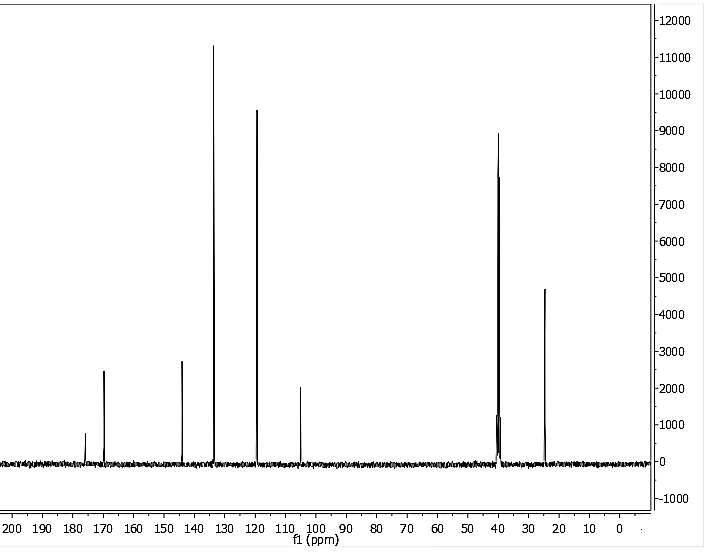


**Figure S60:** ^13^C-NMR spectrum (100 MHz) of *N*-(4-cyanophenyl)acetamide in DMSO-*d_6_*

**
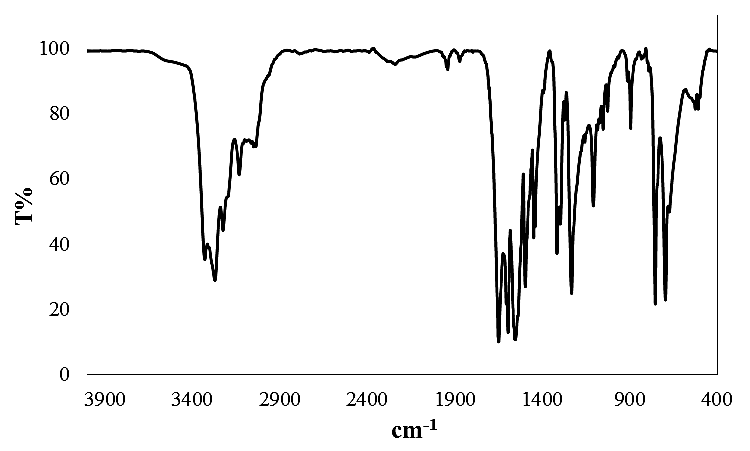
**

**Figure S61:** FT-IR spectrum of 1,3-Diphenyl urea in KBr


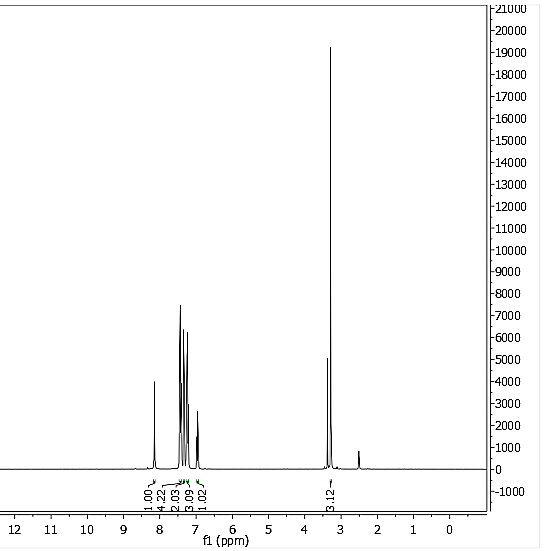


**Figure S62:** ^1^H-NMR spectrum (400MHz) of 1,3-Diphenyl urea in DMSO-*d_6_*


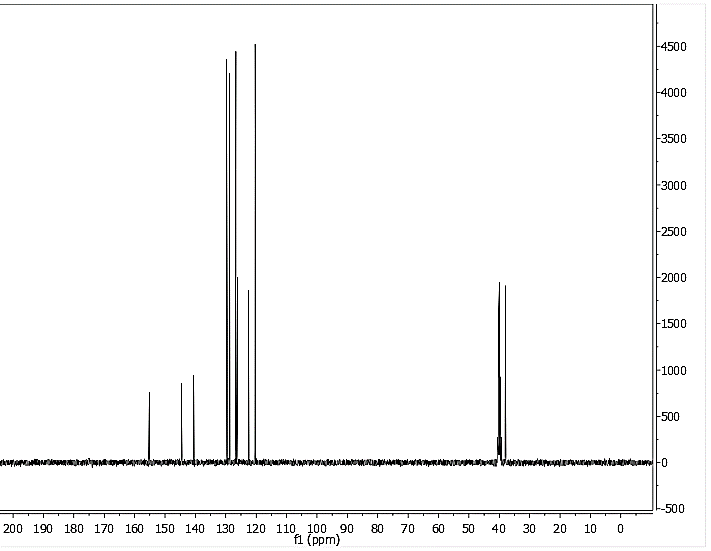


**Figure S63:** ^13^C-NMR spectrum (100 MHz) of 1,3-Diphenyl urea in DMSO-*d_6_*

**
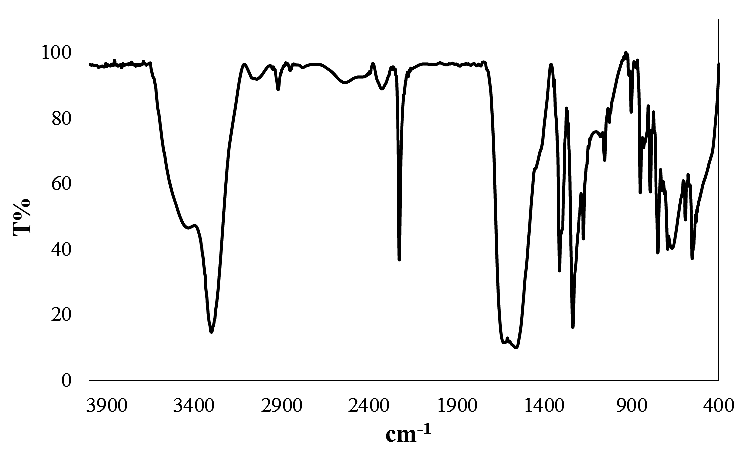
**

**Figure S64:** FT-IR spectrum of 1-(4-Cyanophenyl)-3-phenyl urea in KBr


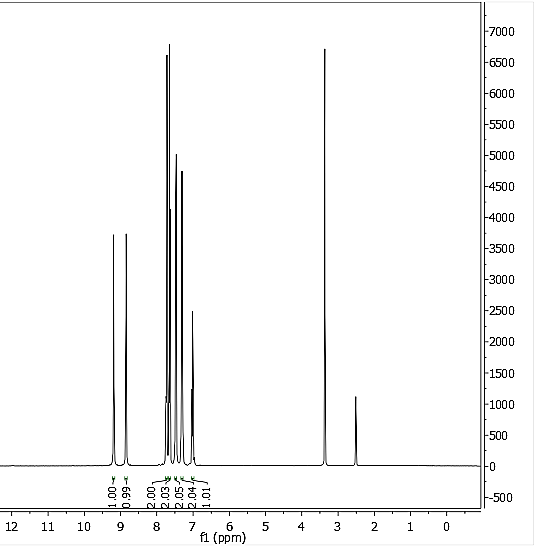


**Figure S65:** ^1^H-NMR spectrum (400MHz) of 1-(4-Cyanophenyl)-3-phenyl urea in DMSO-*d_6_*


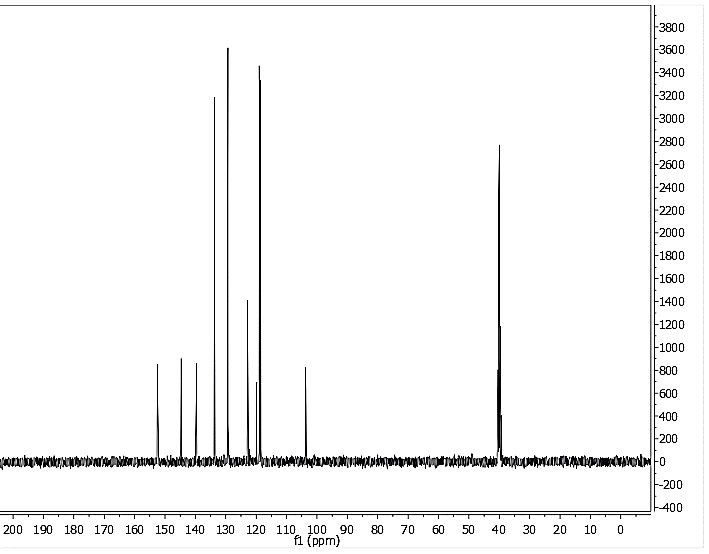


**Figure S66:** ^13^C-NMR spectrum (100 MHz) of 1-(4-Cyanophenyl)-3-phenyl urea in DMSO-*d_6_*

**
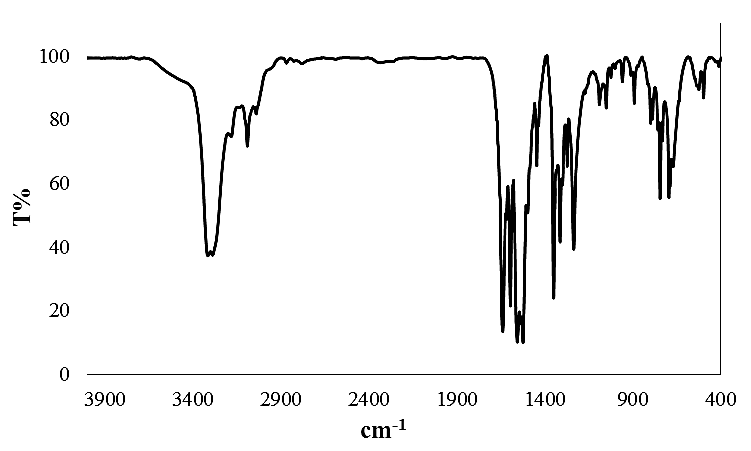
**

**Figure S67:** FT-IR spectrum of 1-(3-Nitrophenyl)-3-phenyl urea in KBr


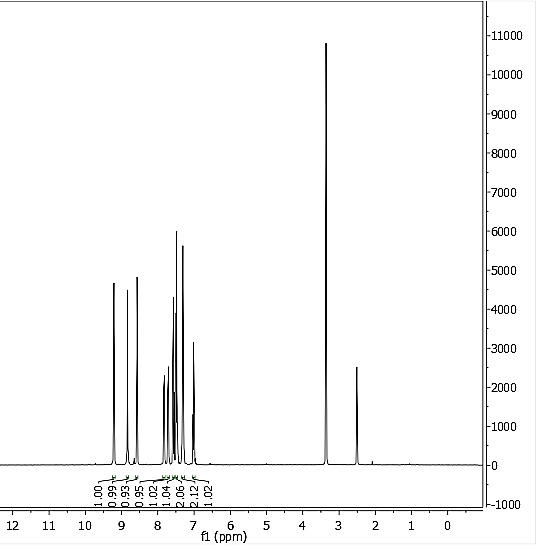


**Figure S68:** ^1^H-NMR spectrum (400MHz) of 1-(3-Nitrophenyl)-3-phenyl urea in DMSO-*d_6_*


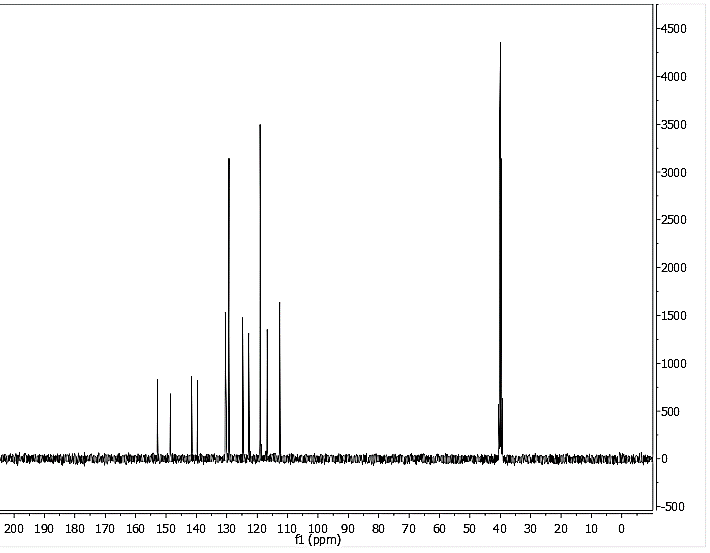


**Figure S69:** ^13^C-NMR spectrum (100 MHz) of 1-(3-Nitrophenyl)-3-phenyl urea in DMSO-*d_6_*


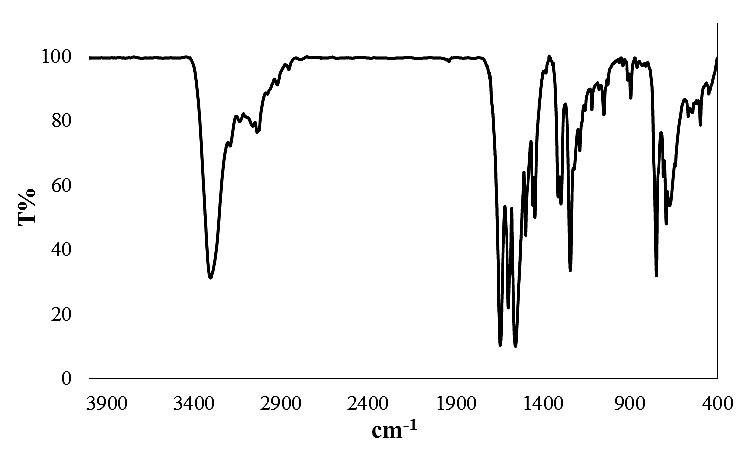


**Figure S70:** FT-IR spectrum of 1-Phenyl-3-(*o*-tolyl) urea in KBr


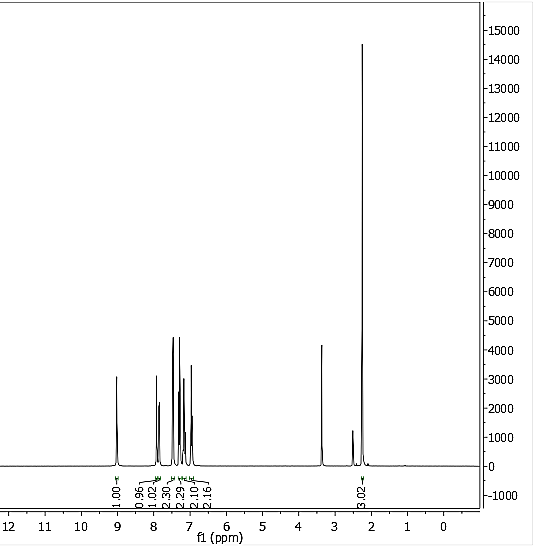


**Figure S71:** ^1^H-NMR spectrum (400MHz) of 1-Phenyl-3-(*o*-tolyl) urea in DMSO-*d_6_*


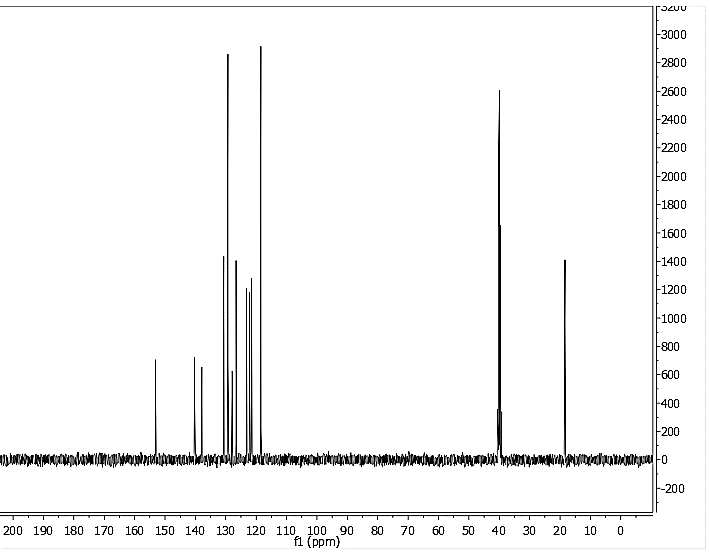


**Figure S72:** ^13^C-NMR spectrum (100 MHz) of 1-Phenyl-3-(*o*-tolyl) urea in DMSO-*d_6_*


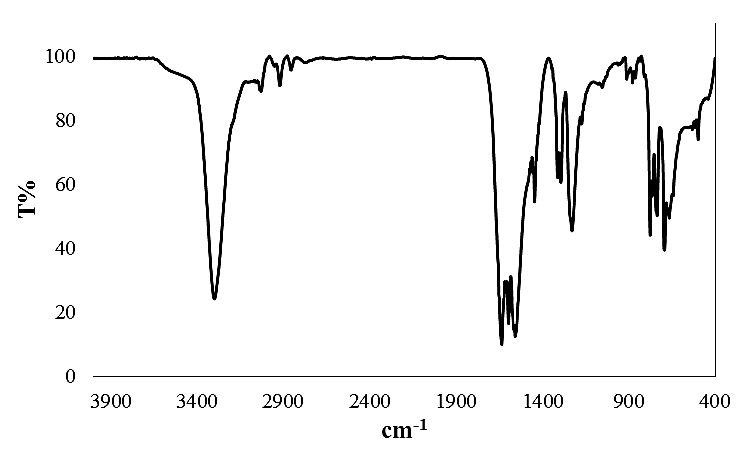


**Figure S73:** FT-IR spectrum of 1-Phenyl-3-(*m*-tolyl) urea in KBr


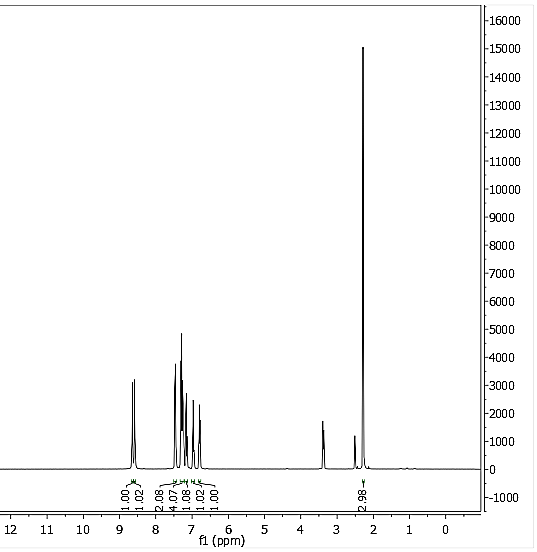


**Figure S74:** ^1^H-NMR spectrum (400MHz) of 1-Phenyl-3-(*m*-tolyl) urea in DMSO-*d_6_*


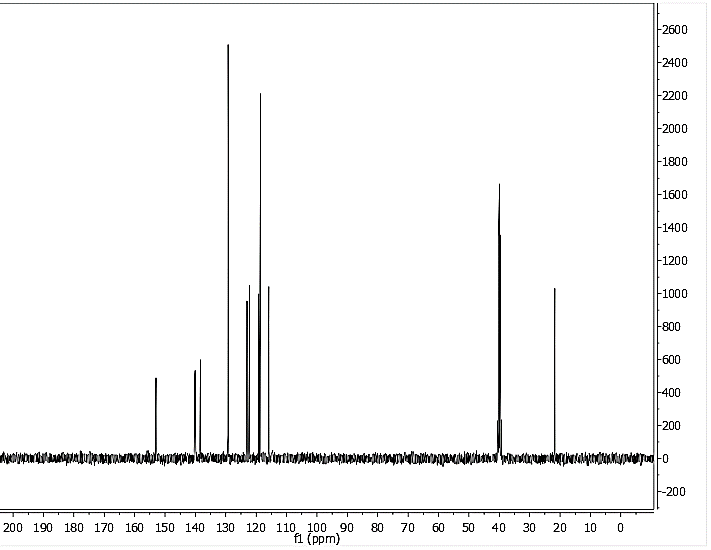


**Figure S75:** ^13^C-NMR spectrum (100 MHz) of 1-Phenyl-3-(*m*-tolyl) urea in DMSO-*d_6_*

**
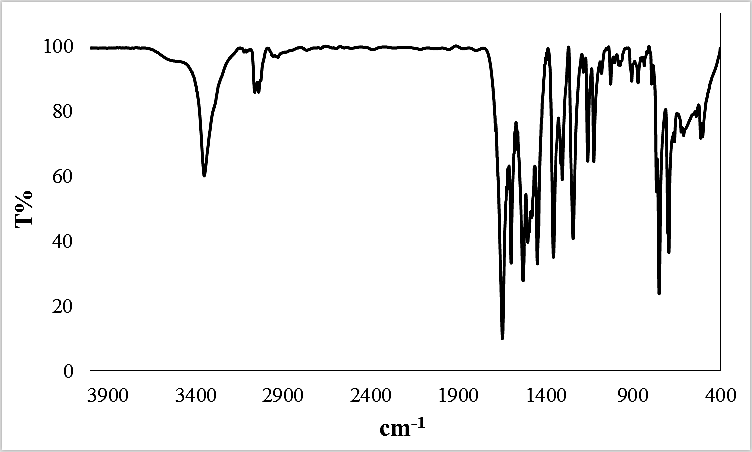
**

**Figure S76:** FT-IR spectrum of 1-Methyl-1,3-diphenyl urea in KBr


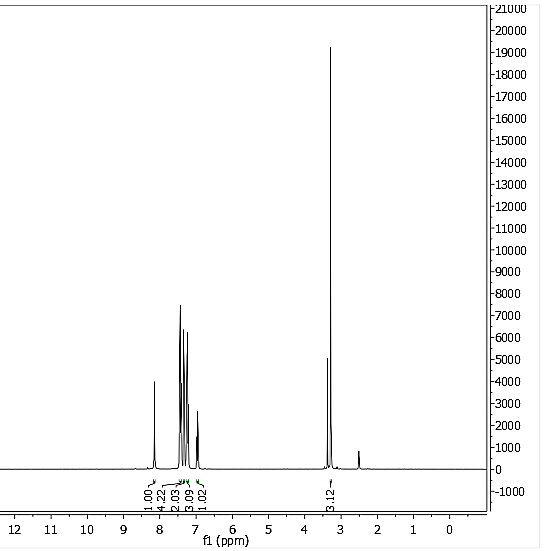


**Figure S77:** ^1^H-NMR spectrum (400MHz) of 1-Methyl-1,3-diphenyl urea in DMSO-*d_6_*


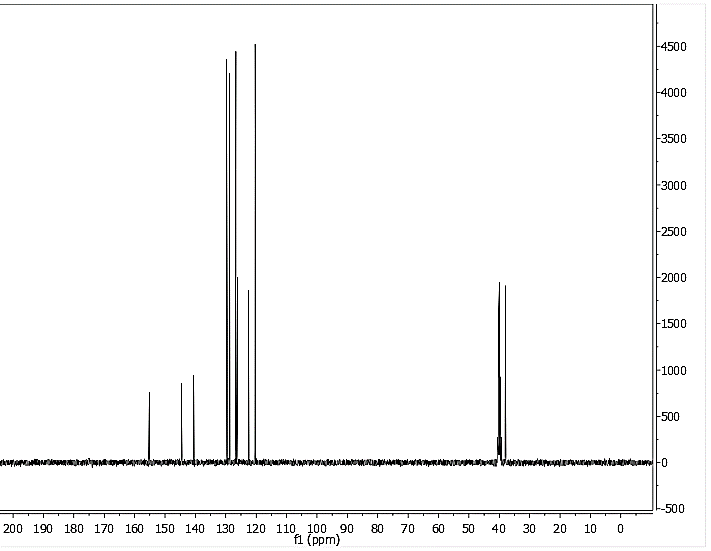


**Figure S78:** ^13^C-NMR spectrum (100 MHz) of 1-Methyl-1,3-diphenyl urea in DMSO-*d_6_*

**
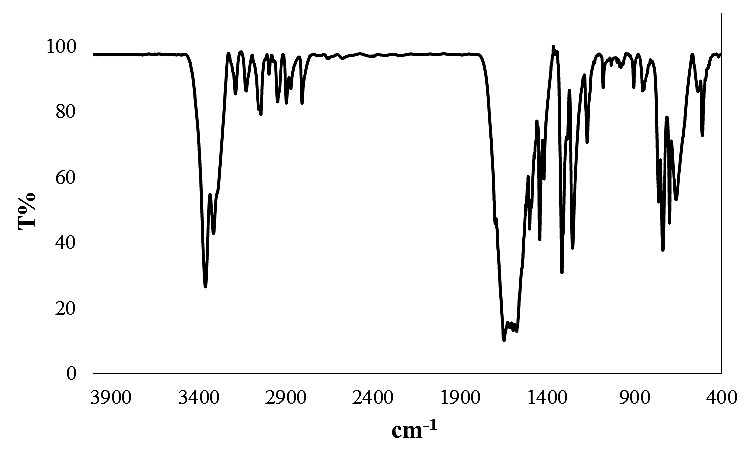
**

**Figure S79:** FT-IR spectrum of 1-Methyl-3-phenyl urea in KBr


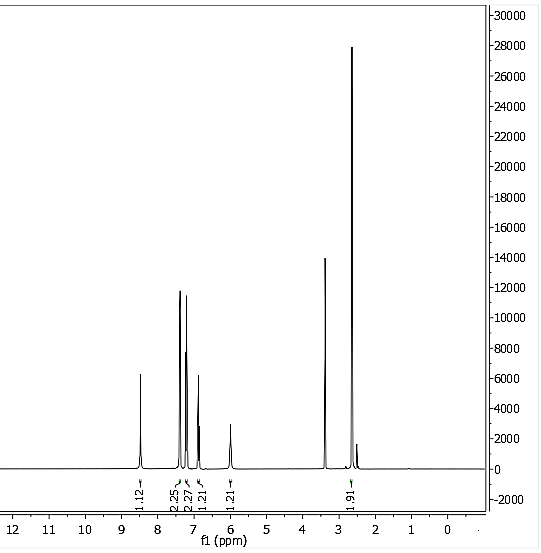


**Figure S80:** ^1^H-NMR spectrum (400MHz) of 1-Methyl-3-phenyl urea in DMSO-*d_6_*


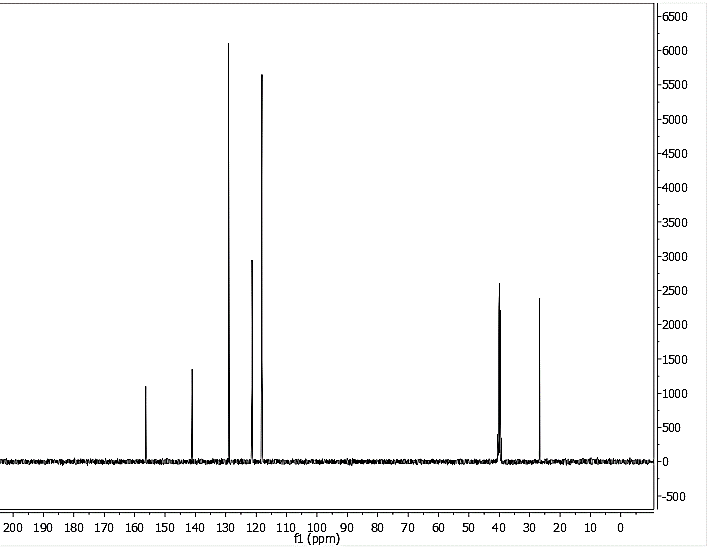


**Figure S81:** ^13^C-NMR spectrum (100 MHz) of 1-Methyl-3-phenyl urea in DMSO-*d_6_*

**
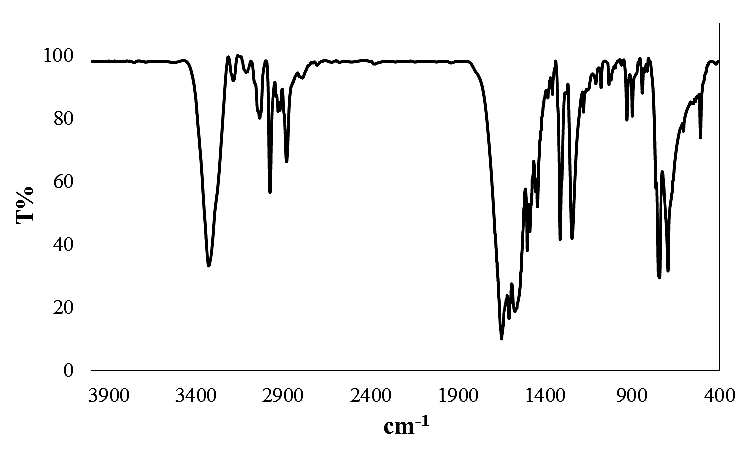
**

**Figure S82:** FT-IR spectrum of 1-Ethyl-3-phenyl urea in KBr


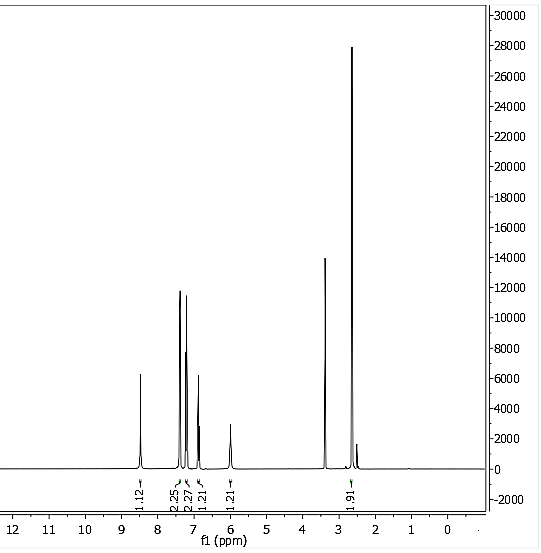


**Figure S83:** ^1^H-NMR spectrum (400MHz) of 1-Ethyl-3-phenyl urea in DMSO-*d_6_*


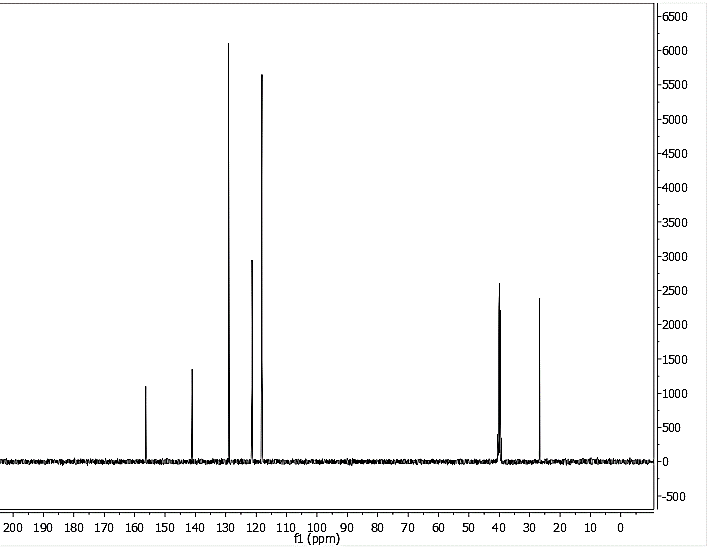


**Figure S84:** ^13^C-NMR spectrum (100 MHz) of 1-Ethyl-3-phenyl urea in DMSO-*d_6_*

**
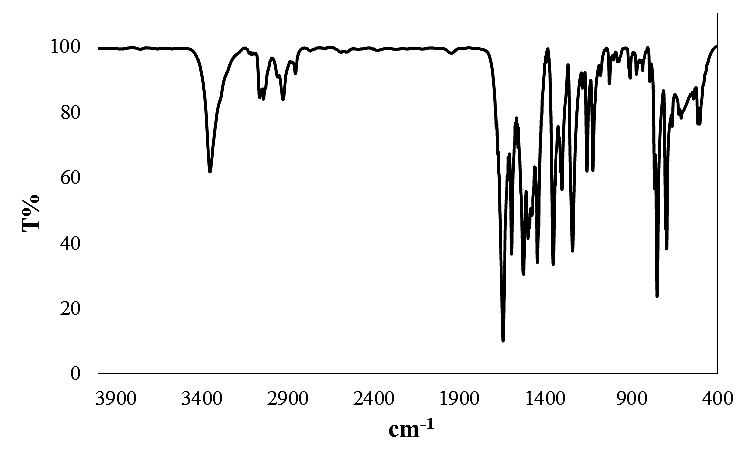
**

**Figure S85:** FT-IR spectrum of 1-Butyl-3-phenyl urea in KBr


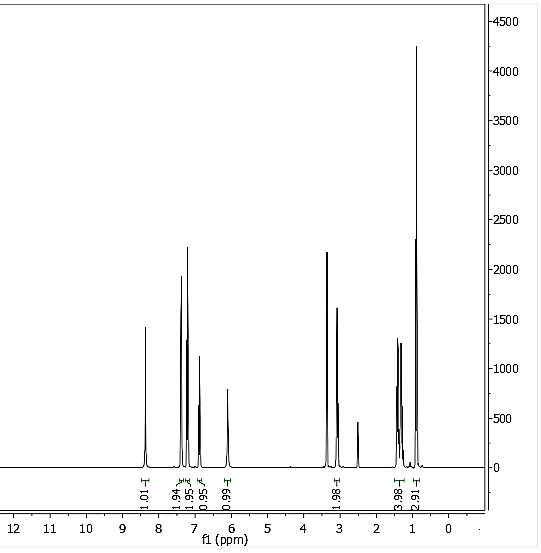


**Figure S86:** ^1^H-NMR spectrum (400MHz) of 1-Butyl-3-phenyl urea in DMSO-*d_6_*


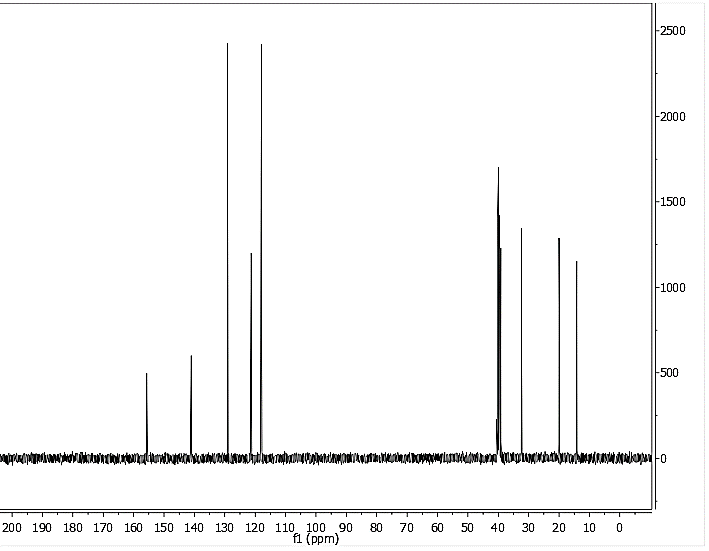


**Figure S87:** ^13^C-NMR spectrum (100 MHz) of 1-Butyl-3-phenyl urea in DMSO-*d_6_*

**
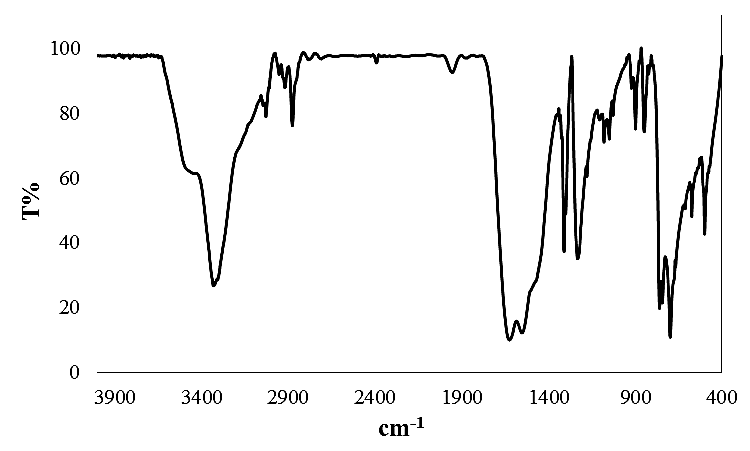
**

**Figure S88:** FT-IR spectrum of 1-Benzyl-3-phenyl urea in KBr


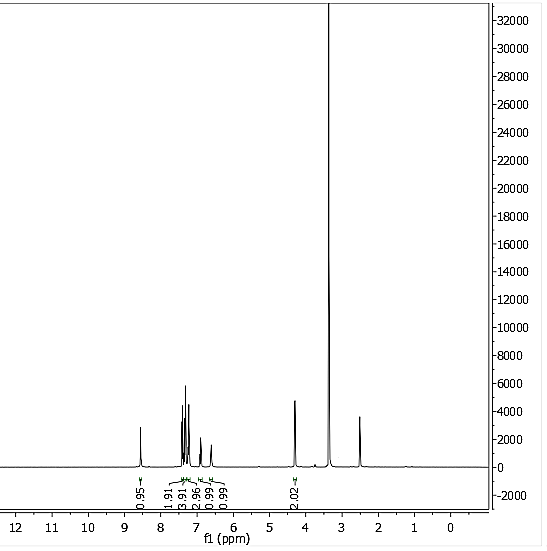


**Figure S89:** ^1^H-NMR spectrum (400MHz) of 1-Benzyl-3-phenyl urea in DMSO-*d_6_*


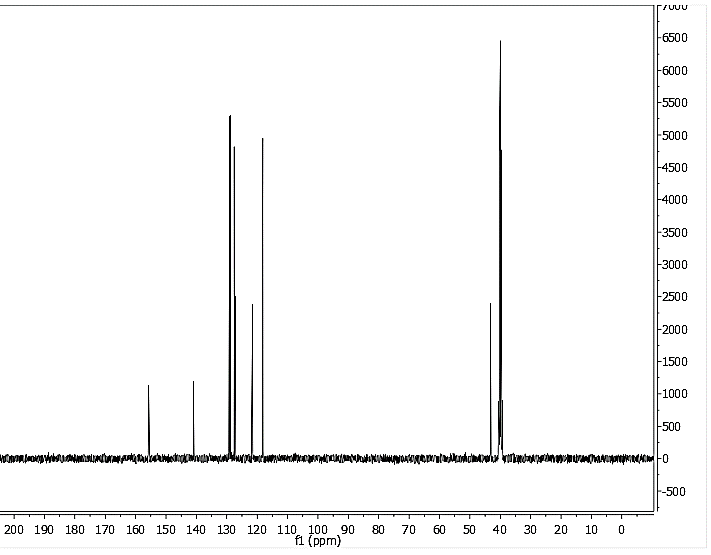


**Figure S90:** ^13^C-NMR spectrum (100 MHz) of 1-Benzyl-3-phenyl urea in DMSO-*d_6_*

**
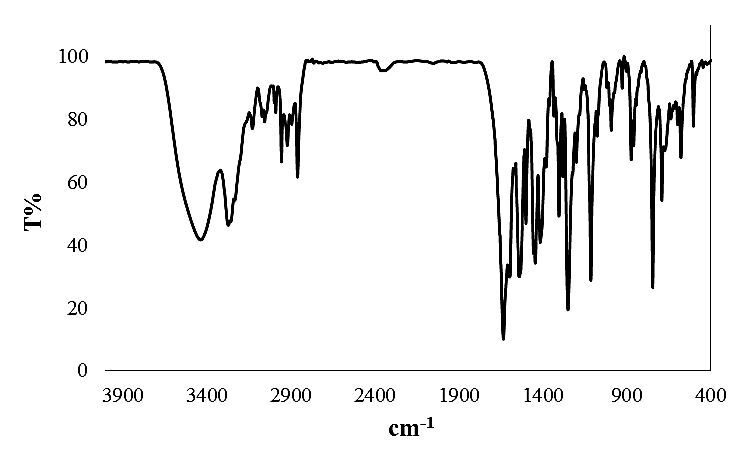
**

**Figure S91:** FT-IR spectrum of *N*-phenylmorpholine-4-carboxamide in KBr


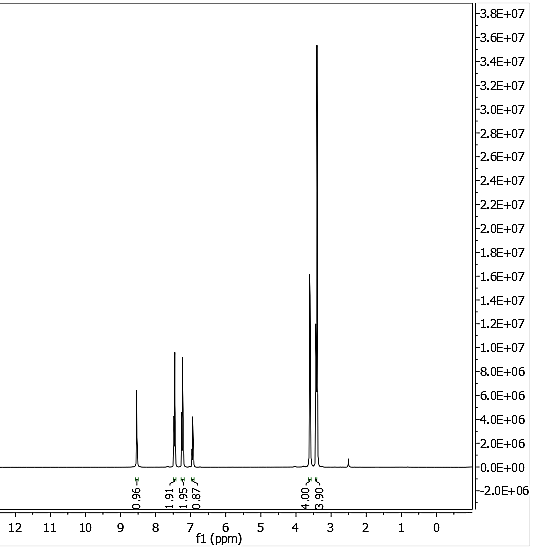


**Figure S92:** ^1^H-NMR spectrum (400MHz) of *N*-phenylmorpholine-4-carboxamide in DMSO-*d_6_*


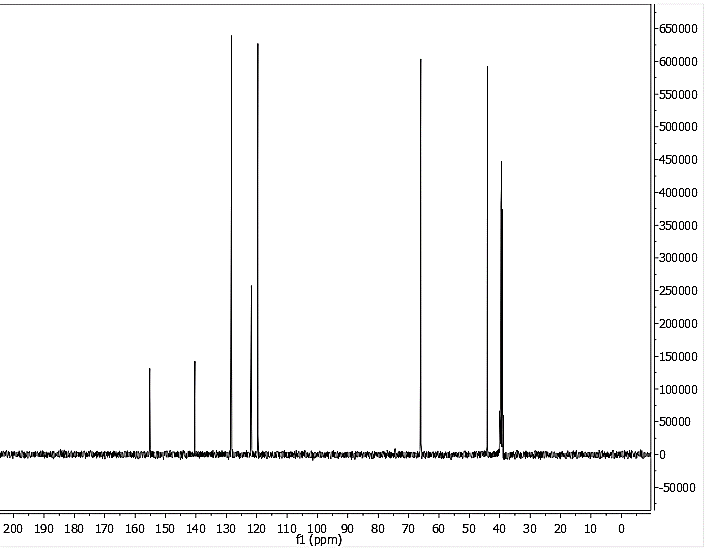


**Figure S93:** ^13^C-NMR spectrum (100 MHz) of *N*-phenylmorpholine-4-carboxamide in DMSO-*d_6_*


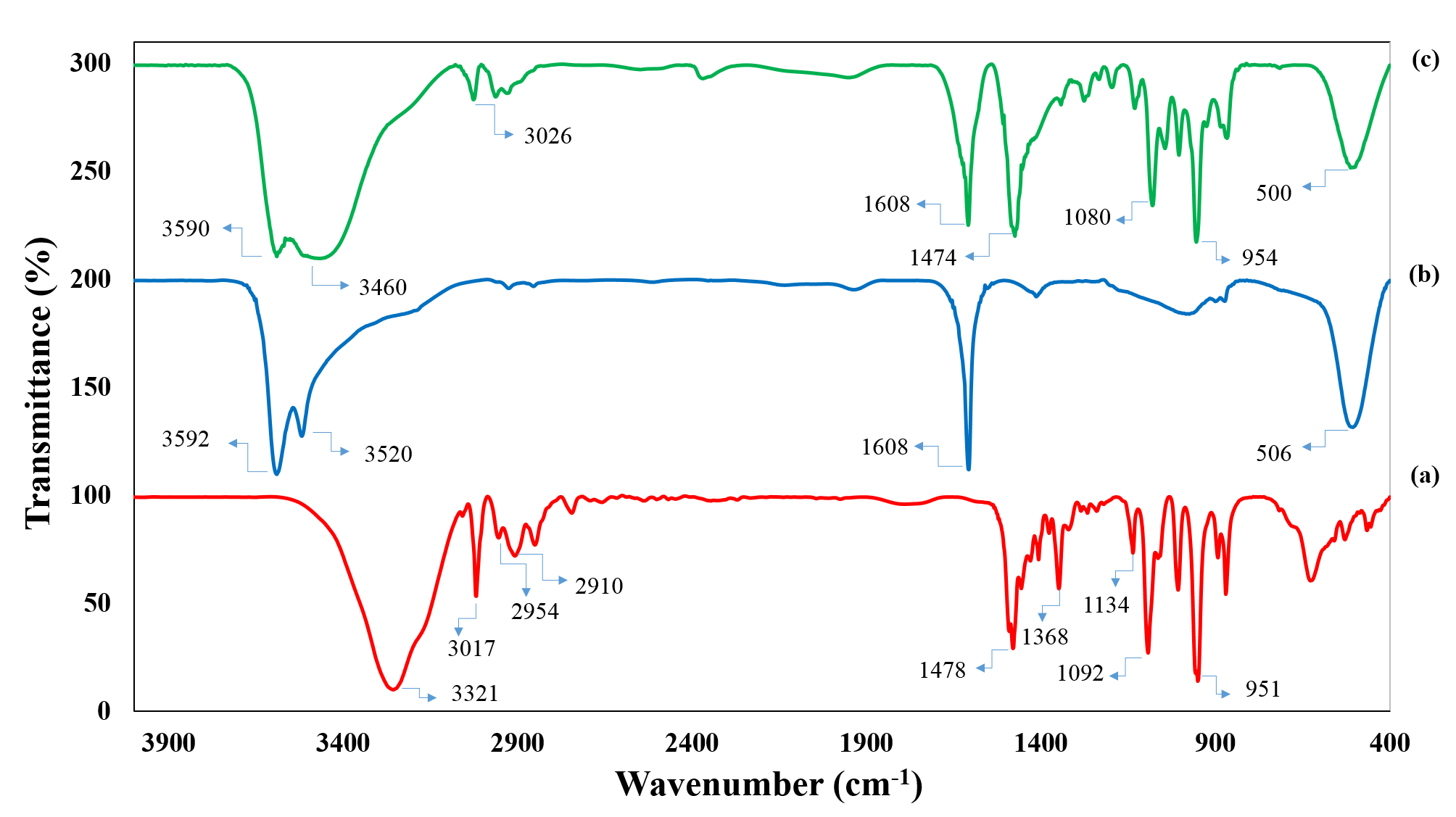


**Figure S94.** FT-IR spectra for a) ChCl, b) ZnCl_2_, c) DES ([ChCl][ZnCl_2_]_2_)

[1] L. Guo, Z. Chen, H. Zhu, M. Li, Y. Gu, Acid-catalyzed chemodivergent reactions of 2, 2-dimethoxyacetaldehyde and anilines, Chinese Chemical Letters, 32 (2021) 1419-1422.

[2] P.A. Koutentis, S.S. Michaelidou, The conversion of 2-cyano cyanothioformanilides into 3-aminoindole-2-carbonitriles using triphenylphosphine, Tetrahedron, 66 (2010) 6032-6039.

[3] F. Shirini, M. Mazloumi, M. Seddighi, Acidic ionic liquid immobilized on nanoporous Na+-montmorillonite as an efficient and reusable catalyst for the formylation of amines and alcohols, Research on Chemical Intermediates, 42 (2016) 1759-1776.

[4] E. Surmiak, C.G. Neochoritis, B. Musielak, A. Twarda-Clapa, K. Kurpiewska, G. Dubin, C. Camacho, T.A. Holak, A. Dömling, Rational design and synthesis of 1, 5-disubstituted tetrazoles as potent inhibitors of the MDM2-p53 interaction, European Journal of Medicinal Chemistry, 126 (2017) 384-407.

[5] S. Kazemi, A. Mobinikhaledi, M. Zendehdel, NaY zeolite functionalized by sulfamic acid/Cu (OAc) 2 as a new and reusable heterogeneous hybrid catalyst for efficient solvent-free formylation of amines, Chinese Chemical Letters, 28 (2017) 1767-1772.

[6] J. Yin, J. Zhang, C. Cai, G.-J. Deng, H. Gong, Catalyst-free transamidation of aromatic amines with formamide derivatives and tertiary amides with aliphatic amines, Organic letters, 21 (2018) 387-392.

[7] J.O. Rathi, G.S. Shankarling, Concentrated solar radiation aided energy efficient and chemoselective protocol for N-acylation and N-formylation reactions in aqueous medium, Solar Energy, 189 (2019) 471-479.

[8] M. Zendehdel, F. Tavakoli, Functionalizing HY zeolite with sulfonic acid, a micro-meso structure reusable catalyst for organic transformations, Journal of the Iranian Chemical Society, 19 (2022) 1095-1107.

[9] M. Jadidi Nejad, A. Heydari, Palladium supported on MRGO@ CoAl‐LDH catalyzed reductive carbonylation of nitroarenes and carbonylative Suzuki coupling reactions using formic acid as liquid CO and H2 source, Applied Organometallic Chemistry, 35 (2021) e6368.

[10] H. Huang, Y. Wu, W. Zhang, C. Feng, B.-Q. Wang, W.-F. Cai, P. Hu, K.-Q. Zhao, S.-K. Xiang, Copper-Catalyzed Regioselective CH Sulfonyloxylation of Electron-Rich Arenes with p-Toluenesulfonic Acid and Sulfonyloxylation of Aryl (mesityl) iodonium Sulfonates, The Journal of Organic Chemistry, 82 (2017) 3094-3101.

[11] F.O. Rodriguez del Rey, P.E. Floreancig, Synthesis of Nitrogen-Containing Heterocycles through Catalytic Dehydrative Cyclization Reactions, Organic Letters, 23 (2020) 150-154.

[12] M. Ran, J. He, B. Yan, W. Liu, Y. Li, Y. Fu, C.-J. Li, Q. Yao, Catalyst-free generation of acyl radicals induced by visible light in water to construct C–N bonds, Organic & Biomolecular Chemistry, 19 (2021) 1970-1975.

[13] X. Peng, Y. Liu, Q. Shen, D. Chen, X. Chen, Y. Fu, J. Wang, X. Zhang, H. Jiang, J. Li, BODIPY Photocatalyzed Beckmann Rearrangement and Hydrolysis of Oximes under Visible Light, The Journal of Organic Chemistry, 87 (2022) 11958-11967.

[14] G. Györke, A. Dancsó, B. Volk, L. Bezúr, D. Hunyadi, I. Szalóki, M. Milen, Direct Use of Copper-Containing Minerals in Goldberg Arylation of Amides, Catalysis Letters, (2022) 1-19.

[15] S. Strekalova, A. Kononov, V. Morozov, O. Babaeva, E. Gavrilova, Y. Budnikova, Electrochemical Approach to Amide Bond Formation, Adv. Synth. Catal., 365 (2023) 3375-3381.

[16] Y. Lu, A. Kasahara, T. Hyodo, K. Ohara, K. Yamaguchi, Y. Otani, T. Ohwada, Isolation and Reactions of Imidoyl Fluorides Generated from Oxime Using the Diethylaminosulfur Trifluoride/Tetrahydrofuran (DAST–THF) System, Org. Lett., (2023).

[17] Y. Zhang, Z. Zhang, Y. Hu, Y. Liu, H. Jin, B. Zhou, Nickel-catalyzed cyanation reaction of aryl/alkenyl halides with alkyl isocyanides, Org. Biomol. Chem., 20 (2022) 8049-8053.

[18] H.G. Schweim, N‐Acyl‐Harnstoffe und ihr Verhalten gegen Amine, Archiv der Pharmazie, 319 (1986) 814-825.

[19] I.D. Inaloo, S. Majnooni, A Fe3O4@ SiO2/Schiff Base/Pd Complex as an Efficient Heterogeneous and Recyclable Nanocatalyst for One‐Pot Domino Synthesis of Carbamates and Unsymmetrical Ureas, European Journal of Organic Chemistry, 2019 (2019) 6359-6368.

[20] C.M. Sanabria, M.T. do Casal, R.B. de Souza, L.C. de Aguiar, M.C. de Mattos, Highly regioselective iodination of N-phenylureas with iodine/trichloroisocyanuric acid, Synthesis, 49 (2017) 1648-1654.

[21] R. Ahmed, R. Gupta, Z. Akhter, M. Kumar, P.P. Singh, TCT-mediated click chemistry for the synthesis of nitrogen-containing functionalities: Conversion of carboxylic acids to carbamides, carbamates, carbamothioates, amides and amines, Org. Biomol. Chem., 20 (2022) 4942-4948.

[22] X. Zhu, M. Xu, J. Sun, D. Guo, Y. Zhang, S. Zhou, S. Wang, Hydroamination and Hydrophosphination of Isocyanates/Isothiocyanates under Catalyst‐Free Conditions, Eur. J. Org. Chem., 2021 (2021) 5213-5218.

[23] H. Valizadeh, L. Dinparast, Microwave-assisted synthesis of symmetrical and unsymmetrical N, N′-disubstituted thioureas and ureas over MgO in dry media, Monatsh. Chem., 143 (2012) 251-254.

[24] T.T. Bui, H.-K. Kim, Lanthanum (III) trifluoromethanesulfonate catalyzed direct synthesis of ureas from N-benzyloxycarbonyl-, N-allyloxycarbonyl-, and N-2, 2, 2-trichloroethoxycarbonyl-protected amines, Synlett, 31 (2020) 997-1002.

[25] L. Wang, H. Wang, Y. Wang, M. Shen, S. Li, Photocatalyzed synthesis of unsymmetrical ureas via the oxidative decarboxylation of oxamic acids with PANI-g-C_3_N_4_-TiO_2_ composite under visible light, Tetrahedron Lett., 61 (2020) 151962.

[26] Q. Spillier, S. Ravez, J. Unterlass, C. Corbet, C. Degavre, O. Feron, R. Frederick, Structure-activity relationships (sars) of?-ketothioamides as inhibitors of phosphoglycerate dehydrogenase (phgdh), (2020).

[27] E. Wiberg, N. Wiberg, Inorganic chemistry, Academic press2001.
